# Methodological assessment of systematic reviews of in-vitro dental studies

**DOI:** 10.1186/s12874-022-01575-z

**Published:** 2022-04-13

**Authors:** Christopher Hammel, Nikolaos Pandis, Dawid Pieper, Clovis Mariano Faggion

**Affiliations:** 1 Department of Periodontology and Operative Dentistry, Faculty of Dentistry, University Hospital Münster, Münster, Germany; 2 Department of Orthodontics and Dentofacial Orthopedics, Dental School/Medical Faculty, University of Bern, Bern, Switzerland; 3 Faculty of Health Sciences Brandenburg, Brandenburg Medical School, (Theodor Fontane), Institute for Health Services and Health Systems Research, Rüdersdorf, Germany; 4 Brandenburg Medical School, Center for Health Services Research, (Theodor Fontane), Rüdersdorf, Germany

**Keywords:** Systematic reviews, Methods, Methodological study, In-vitro, AMSTAR-2, Methodology

## Abstract

**Background:**

Systematic reviews of in-vitro studies, like any other study, can be of heterogeneous quality. The present study aimed to evaluate the methodological quality of systematic reviews of in-vitro dental studies.

**Methods:**

We searched for systematic reviews of in-vitro dental studies in PubMed, Web of Science, and Scopus databases published up to January 2022. We assessed the methodological quality of the systematic reviews using a modified “A MeaSurement Tool to Assess systematic Reviews” (AMSTAR-2) instrument. The 16 items, in the form of questions, were answered with yes, no, or py (partial yes). Univariable and multivariable linear regression models were used to examine the association between systematic review characteristics and AMSTAR-2 percent score. Overall confidence in the results of the systematic reviews was rated, based on weaknesses identified in critical and non-critical AMSTAR-2 items.

**Results:**

The search retrieved 908 potential documents, and after following the eligibility criteria, 185 systematic reviews were included. The most researched topics were ceramics and dental bonding. The overall rating for the confidence in the results was critically low in 126 (68%) systematic reviews. There was high variability in the response among the AMSTAR-2 items (0% to 75% positively answered). The univariable analyses indicated dental specialty (*p* = 0.03), number of authors (coef: 1.87, 95% CI: 0.26, 3.47, *p* = 0.02), and year of publication (coef: 2.64, 95% CI: 1.90, 3.38, *p* < 0.01) were significantly associated with the AMSTAR-2 percent score. Whereas, in the multivariable analysis only specialty (*p* = 0.01) and year of publication (coef: 2.60, 95% CI: 1.84, 3.35, *p* < 0.001) remained significant. Among specialties, endodontics achieved the highest AMSTAR-2 percent score.

**Conclusions:**

The methods of systematic reviews of in vitro dental studies were suboptimal. Year of publication and dental specialty were associated with AMSTAR-2 scores. The overall rating of the confidence in the results was low and critically low for most systematic reviews.

**Supplementary Information:**

The online version contains supplementary material available at 10.1186/s12874-022-01575-z.

## Introduction

In-vitro experiments are important to test new potentially promising therapies that might be incorporated in clinical practice. Usually, higher levels of evidence, in the form of large randomized controlled trials (RCTs), are needed to confirm the efficacy of therapies [1]. However, to reach this point of testing, basic evidence is often necessary for the initial assessment of the behavior and potential benefits of new therapies. A common sequence of testing for new therapies begins in an in-vitro environment and if the new treatment shows potential, it can be further tested in animals and finally in humans [2].

Systematic reviews have the ability to accumulate evidence from primary studies to address relevant research questions. In the presence of primary study homogeneity, meta-analyses can be used to calculate the pooled treatment effect [3]. As with any primary study, a systematic review should also be evaluated for its methodological rigor. Although, the quality of the primary study cannot be improved through a systematic review, a well-conducted and reported systematic review can provide information as to whether the primary studies can be trusted. Systematic reviews of in-vitro studies can map and possibly synthesize evidence about a new approach considered for clinical use as well as identify the heterogeneity of the treatment effects.

Among several quality assessment tools for systematic reviews [4], the most researched and most often used tool is the “A MeaSurement Tool to Assess systematic Reviews (AMSTAR)” [5]. AMSTAR was introduced and validated in 2007 [6], updated in 2017 (AMSTAR-2) [7], and has become the standard means to evaluate the methodological quality of systematic reviews.

To the best of our knowledge, there are no reports assessing the methodological quality of SR of in-vitro studies. Therefore, the primary aim of this research-on-research study was to assess, using the AMSTAR-2 checklist, the methodological quality of systematic reviews of in-vitro dental studies and determine the overall confidence in the results of the systematic reviews selected. As a secondary aim, we investigated the potential association between systematic review characteristics and methodological quality.

## Material and methods

This methodological study was planned to answer the following primary question: What is the methodological quality of systematic reviews of in-vitro experiments in dentistry?

### Search strategy

On 09 January 2020, we searched in the PubMed, Web of Science, and Scopus databases for systematic reviews of in-vitro studies published in dentistry from database inception up to January 2020. The search was updated on 06 January 2022 and involved the Scopus and Web of Science databases only, because PubMed eliminated one filter used in the original search, and therefore, the search could not be repeated in this database. In the PubMed database, the keywords “in vitro” OR in-vitro were used together with the filters “systematic reviews”, and “dental journals”. In the Scopus database, the same keywords were used together with the filters “review” and “dentistry”. In the Web of Science database, the same keywords were also used in combination with the filters “dentistry”, “oral surgery”, and “medicine”. We also searched for potential systematic reviews in the reference lists of systematic reviews retrieved from the electronic searches. The search was performed by two authors (CMF and CH) and it is reported in the supplementary file (Additional file 1).

### Eligibility criteria

We included systematic reviews of in-vitro and ex-vivo studies on interventions published in any dental specialty. A review was considered systematic when authors reported the aim of conducting a systematic review. Non-systematic reviews and other types of study design were excluded as well as systematic reviews published in dentistry involving humans or animals. Systematic reviews including data from mixed subjects, for example, in-vitro and animal or clinical research, were also excluded. Systematic reviews not published in English were excluded.

### Data selection

Two reviewers (CMF, CH) selected data from a sample of eligible studies and achieved good agreement (at least 80 percent), with the remainder selected by one reviewer (CH) [7]. At this stage, documents not meeting the eligibility criteria were excluded and reasons for exclusion were recorded. The remaining titles had their full-text evaluated by the author and those not meeting the eligibility criteria were also excluded and, again, the reasons for exclusion recorded.

### Data extraction

Two reviewers (CMF, CH) extracted data from a sample of eligible studies and achieved good agreement (at least 80 percent), with the remainder extracted by one reviewer (CH) [7]. The following data were extracted from the included systematic reviews: a) dental specialty; b) topic of research; c) country and continent of the first author; d) systematic review with or without meta-analysis; e) number of in-vitro and ex-vivo experiments included; f) name of the dental journal; g) journal impact factor (IF); h) number of citations; i) topic of the research group and j) number of authors k) publication year.

### Methodological assessment of systematic reviews

Given that a validated instrument specifically for systematic reviews of in-vitro studies does not exist and that key methodological issues are similar across study types we assessed the methodological quality of included systematic reviews using the AMSTAR-2 tool [7]. The 16 items in the form of questions were answered with *yes*, *partial yes (py),* or *no*. The answer *yes* means that the item was fully met by the systematic review, while *py* means that the systematic review only met the AMSTAR-2 recommendations for that specific item partially. To facilitate the statistical analysis, we assigned an ordinal score per item ranging from 0 to 2, with 0 = *no*, 1 = *py* and 2 = *yes*.

We further evaluated the AMSTAR-2 critical domains [7]. These seven domains (items 2, 4, 7, 9, 11, 13, and 15) correspond to the comprehensiveness of the literature search, eligibility criteria, Risk of Bias (RoB) analysis and interpretation, appropriateness of meta-analysis, and potential impact of publication bias [7]. Following the suggestions from the AMSTAR-2 developers [7], the overall confidence in the results of the review was rated in four levels: high, moderate, low, and critically low. These levels were based on weaknesses identified in critical and non-critical items. One critical flaw would mean low confidence in the results, and more than one critical flaw in a specific item would mean critically low confidence. Up to one non-critical flaw, without any critical flaw, would generate high confidence in the results. To rate the confidence as moderate, more than one non-critical weakness and no critical flaw should be presented. Additional file 2, supplementary file, reports the rationale in more detail.

To increase homogeneity in the assessment, two reviewers (CMF, CH) performed three rounds of assessment with three systematic reviews (*n* = 9) before a full assessment of the selected sample commenced. Subsequently, the same reviewers assessed data from a sample of eligible studies and achieved good agreement (at least 80 percent), with the remainder assessed by one reviewer (CH) [7]. We calculated the interrater reliability agreement between the two assessors using the kappa statistics.

The final data extraction form was checked at random by the second reviewer (CMF) and potential disagreements were further discussed for consensus. Thirteen items of 16 were applicable if the SRs were conducted without meta-analysis (three items are exclusively related to the conduct of meta-analysis). Because the AMSTAR-2 checklist was originally developed to evaluate systematic reviews of clinical studies, we adapted some of the sub-items or signaling questions of the AMSTAR-2 items to improve applicability to systematic reviews of in-vitro experiments (Additional file 3).

### Statistical analysis

Frequency distributions of specific study characteristics in the included reviews were examined and individual AMSTAR-2 ratings were tabulated and the percent quality score per specialty was calculated. The primary outcome was a percent quality score calculated using all applicable AMSTAR-2 items per systematic review and using the formula:

$$Percent=\left[sum/\left(2^\ast eligible\;items\right)\right]^\ast100$$

The sum was calculated by adding the scores (0/1/2) across items per study and by dividing by the maximum score of applicable items. The maximum score per study would be 32 if all AMSTAR-2 items were applicable. The not applicable items were 11, 12, and 15 when systematic reviews did not include a meta-analysis.

Data were further analyzed on an exploratory basis through univariable and multivariable linear regression; the multivariable analysis included the significant predictors from the univariable analyses. The following independent variables (characteristics) were assessed: the number of authors, dental specialty, the continent of the first author, IF, and year of publication.

A two-tailed *p*-value at 5% statistical significance was used and all analyses were performed with the STATA version 17.0 software (Stata Corporation, College Station, TX, USA).

## Results

**Fig. 1:**
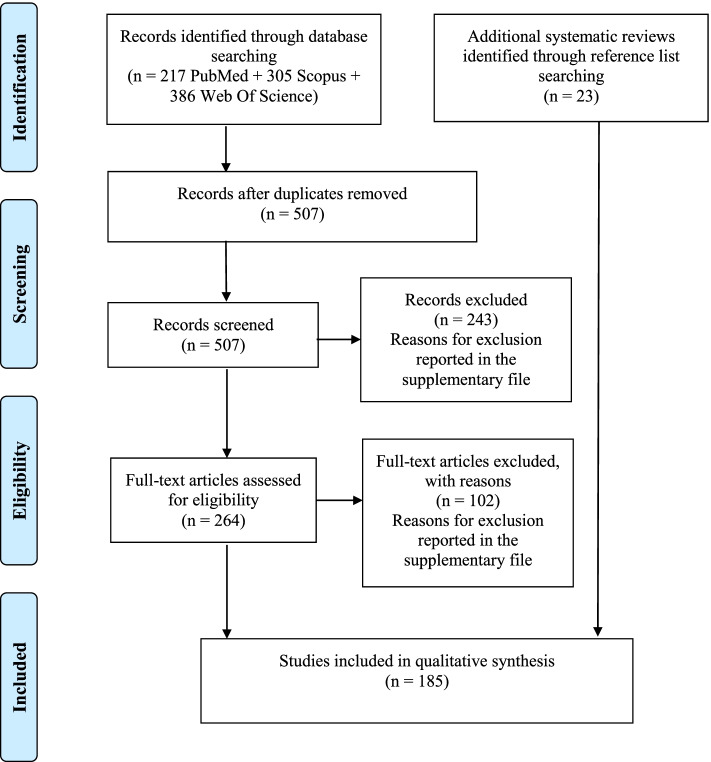
Flowchart of the search and selection processes

Out of the 908 initially identified articles, 185 qualified for inclusion in the present study (Additional file 4). The reasons for the exclusion of each study are reported in the supplementary file (Additional files 5 and 6). The flowchart of the literature search and selection is depicted in Fig. 1.

### Characteristics of systematic reviews

Systematic reviews were published in six different dental specialties with prosthodontics (*n* = 59, 31.9%), and restorative dentistry (*n* = 49, 26.5%) being the most prevalent. The most researched topics (46/185) were ceramics and dental bonding. First authors from Brazil were reported in 67 (36.2%) reviews and eighty-five (45.9%) systematic reviews included a meta-analysis. The full report of the characteristics of the systematic reviews is depicted in Table 1.

**Table 1:** Characteristics of 185 included articles

Article characteristics	Frequency (%)
**Dental specialty**	
Endodontics	43 (23.2)
Orthodontics	20 (10.8)
Orthognathic Surgery	1 (0.5)
Periodontology	13 (7)
Prosthodontics	59 (31.9)
Restorative Dentistry	49 (26.5)
**Topic of Research**	
Accuracy	5 (2.7)
Adhesives	12 (6.5)
Aligner-type appliances	1 (0.5)
Antibacterial activity	1 (0.5)
Antimicrobial Efficacy	1 (0.5)
Avulsed teeth	2 (1.1)
Bleaching	1 (0.5)
Bonding	23 (12.4)
Bone regeneration	1 (0.5)
Brackets	6 (3.2)
CAD/CAM	1 (0.5)
Carciogenesis	2 (1.1)
Caries detection	1 (0.5)
Cements	6 (3.2)
Ceramics	23 (12.4)
Composites	6 (3.2)
Cytotoxicity	1 (0.5)
Effect of nicotine	1 (0.5)
Effect of platelet-rich fibrin	1 (0.5)
Fatigue test	2 (1.1)
Fiber Posts	9 (4.9)
Fracture resistance	5 (2.7)
Hydrocolloid	1 (0.5)
Implants	9 (4.9)
Irrigation	7 (3.8)
Laser	2 (1.1)
Microorganisms	1 (0.5)
Molecular Pathways	1 (0.5)
Mouthwashes	1 (0.5)
Nickel-titanium instruments	4 (2.2)
Obturation	2 (1.1)
Orthodontic debonding	1 (0.5)
Periodontal ligament	4 (2.2)
Precision	1 (0.5)
Prostheses	10 (5.4)
Remineralization	3 (1.6)
Restorative strategies	4 (2.2)
Root canal filling	5 (2.7)
Root fracture	1 (0.5)
Sagittal split ramus osteotomy	1 (0.5)
Sealers	5 (2.7)
Sliding in Orthodontics	1 (0.5)
Sterilization	2 (1.1)
Titanium surfaces	6 (3.2)
Tooth reattachment	1 (0.5)
Workflow	1 (0.5)
**Topic of Research Group**	
Cells	22 (11.9)
Materials	105 (56.8)
Parameters	23 (12.4)
Techniques	35 (18.9)
**Country of first author**	
Australia	4 (2.2)
Austria	2 (1.1)
Belgium	1 (0.5)
Bosnia and Herzegovina	1 (0.5)
Brazil	67 (36.2)
Canada	7 (3.8)
China	6 (3.2)
Colombia	2 (1.1)
Egypt	1 (0.5)
Finland	1 (0.5)
France	1 (0.5)
Germany	4 (2.2)
Greece	3 (1.6)
Guayana	1 (0.5)
Hong Kong	1 (0.5)
India	11 (6)
Iran	6 (3.2)
Ireland	1 (0.5)
Israel	1 (0.5)
Italy	4 (2.2)
Jordan	1 (0.5)
Korea	3 (1.6)
Lithuania	1 (0.5)
Malaysia	4 (2.2)
Mexico	2 (1.1)
New Zealand	1 (0.5)
Pakistan	1 (0.5)
Poland	3 (1.6)
Portugal	3 (1.6)
Saudi Arabia	3 (1.6)
Serbia	1(0.5)
Spain	8 (4.3)
Sweden	1 (0.5)
Switzerland	8 (4.3)
The Netherlands	5 (2.7)
Turkey	4 (2.2)
UK	4 (2.2)
United Arab Emirates	1 (0.5)
Uruguay	1 (0.5)
USA	4 (2.2)
Venezuela	1 (0.5)
**Continent of first author**	
Africa	1 (0.5)
America	84 (45.4)
Asia	41 (22.2)
Europe	54 (29.2)
Oceania	5 (2.7)
**Systematic review with or without meta-analysis**	
Systematic review without meta-analysis	100 (54.1)
Systematic review with meta-analysis	85 (45.9)
**Number of experiments included**	
Median (IQR)	17 (19.5)
**Number of authors**	
Median (IQR)	5 (2)
**Impact factor (IF)**	
Median (IQR)	3.4 (1.7)
**Number of citations (Google)**	
Median (IQR)	16.5 (53)

Cells: SRs about bacteria, cell lines, microorganisms; Materials: SRs about dental materials; Parameters: SRs about parameters like bond strength, fracture resistance, fatigue test, accuracy, precision, etc.; Techniques: SRs about techniques like canal obturation, canal disinfection, irrigation, etc.

Percentages may not sum to 100 due to rounding of values

### Methodological quality

**Fig. 2:**
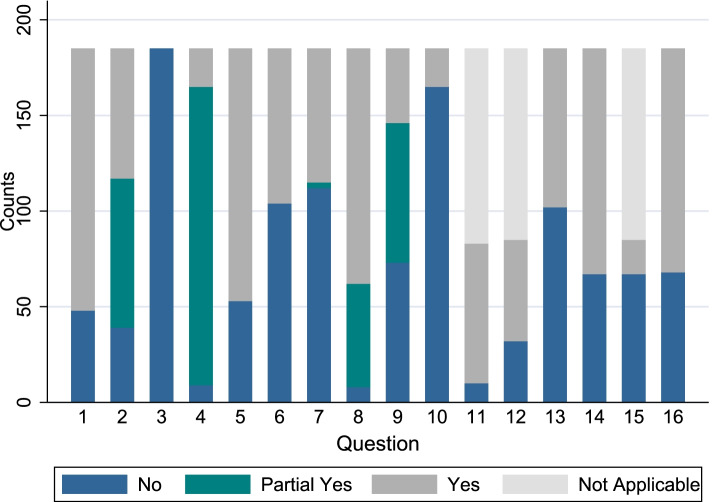
Probability of AMSTAR-2 response per question

The overall rating of the confidence in the results was as follows: high 0 (0%), moderate 16 (9%), low 43 (23%), and critically low 126 (68%). There was great variability in the scores among AMSTAR-2 items. Item 3 received a *no* score in all systematic reviews of this sample. In contrast, items 1 and 5 received a *yes* score on 137 (74.1%) and 132 (71.4%) systematic reviews, respectively. Item 4 received the greatest number of *py* scores (84.4%). For the items specifically related to meta-analysis (items 11,12 and 15), item 11 received the greatest number of yes scores (40.5%). In contrast, item 15 received the greatest number of *no* scores (36.2%). The interrater reliability between the two (CMF, CH) assessors was 0.92. The complete scores of all AMSTAR-2 items are reported in Table 2, Fig. 2, and in the supplementary file (Additional file 7).

**Table 2:** AMSTAR-2 scores across from the 185 systematic reviews of in-vitro dental studies

AMSTAR-2 item	No N(%)	Probably Yes N(%)	Yes N(%)	Not Applicable N(%)
1. Did the research questions and inclusion criteria for the review include the components of PICO?	48(25.9)	0(0)	137(74.1)	0(0)
2. Did the report of the review contain an explicit statement that the review methods were established prior to the conduct of the review?	39(21.1)	78(42.2)	68(36.8)	0(0)
3. Did the review authors explain their selection of the study designs for inclusion in the review?	185(100)	0(0)	0(0)	0(0)
4. Did the review authors use a comprehensive literature search strategy?	9(4.9)	156(84.4)	20(10.8)	0(0)
5. Did the review authors perform study selection in duplicate?	53(28.7)	0(0)	132(71.4)	0(0)
6. Did the review authors perform data extraction in duplicate?	104(56.2)	0(0)	81(43.8)	0(0)
7. Did the review authors provide a list of excluded studies and justify the exclusions?	112(60.5)	3(1.6)	70(37.8)	0(0)
8. Did the review authors describe the included studies in adequate detail?	8(4.3)	54(29.2)	123(66.5)	0(0)
9. Did the review authors use a satisfactory technique for assessing the risk of bias (RoB) in individual studies that were included in the review?	73(39.5)	73(39.5)	39(21.1)	0(0)
10. Did the review authors report on the sources of funding for the studies included in the review?	165(89.2)	0(0)	20(10.8)	0(0)
11. If meta-analysis was performed did the review authors use appropriate methods for statistical combination of results?	10(5.4)	0(0)	75(40.5)	100(54.1)
12. If meta-analysis was performed. did the review authors assess the potential impact of RoB in individual studies on the results of the meta-analysis or other evidence synthesis?	32(17.3)	0(0)	53(28.7)	100(54.1)
13. Did the review authors account for RoB in individual studies when interpreting/ discussing the results of the review?	102(55.1)	0(0)	83(44.9)	0(0)
14. Did the review authors provide a satisfactory explanation for. and discussion of. any heterogeneity observed in the results of the review?	67(36.2)	0(0)	118(63.8)	0(0)
15. If they performed quantitative synthesis did the review authors carry out an adequate investigation of publication bias (small study bias) and discuss its likely impact on the results of the review?	67(36.2)	0(0)	18(9.7)	100(54.1)
16. Did the review authors report any potential sources of conflict of interest. including any funding they received for conducting the review?	68(36.8)	0(0)	117(63.2)	0(0)

Percentages may not sum to 100 due to rounding of values

### Additional analyses

The percent score for orthodontics was 44.88 (standard deviation [SD] 22.76), for periodontology 47.92 (SD 25.69), for restorative dentistry 48.68 (SD 15.82), for endodontics 57.70 (SD 17.17), and prosthodontics 46.56 (SD 19.30). In the univariable analysis, there was evidence of association between the percent score and specialty, number of authors, and year of publication. In the multivariable analysis, only specialty (Likelihood ratio test *p* = 0.01) and year of publication remained significant (Table 3). Specifically, for each additional year, the AMSTAR-2 percent score increased on average by 2.6 units (95% CI: 1.84, 3.35).

**Table 3:** Univariable and multivariable estimates with corresponding 95% confidence intervals and *p*-values for the association among the AMSTAR-2 score and systematic review characteristics

**Variable**		**Univariable**		**Multivariable**	
		**Coeff. (95% CIs)**	***p-*** **value**	**Coeff. (95% CIs)**	***p-*** **value**
**Dental specialty** ^**a**^	Orthodontics	reference	-	reference	-
	Periodontology	3.05 (10.22,16.32)	0.65	2.04 (-9.79,13.86)	0.73
	Restorative	3.80 (-6.08,13.69)	0.45	-0.82 -9.79,8.14)	0.86
	Endodontics	12.82(2.74,22.90)	0.01`	8.97 (-0.15,18.08)	0.05
	Prosthodontics	1.68 (-7.96,11.31)	0.73	-1.48 (-10.12,7.17)	0.74
**Continent**	Americas	Reference			
	Asia & Other	-0.33 (-7.27,6.61)	0.93		
	Europe	-2.59 (-9.27,4.09)	0.45		
**Number of authors**	Per unit	1.87 (0.26,3.47)	0.02	0.48(-1.01, 1.98)	0.53
**Year**	Per unit	2.64(1.90, 3.38)	< 0.01	2.60(1.84, 3.35)	< 0.001
**Impact factor**	Per unit	2.03(-0.84, 4.89)	0.16		

*CI* confidence interval ^a^Likelihood ratio test *p* = 0.01

## Discussion

In-vitro experiments usually test new hypotheses or aim to provide insights into the behaviour of new materials, and systematic reviews compile the best evidence from individual studies to answer relevant research questions. Although systematic reviews are usually focused on answering clinical questions, they can also be applied to animal [8] and in-vitro [9] studies. Assessment of the systematic review quality is an important requirement to correctly evaluate and interpret the results of the included studies. To our knowledge, this is the first review to evaluate the methodological quality of systematic reviews of in-vitro experiments in dentistry and, we think, it will be important in mapping the area and in guiding the conduct of reviews on basic dental research. A smaller previous study was identified which, however, focused only on the reporting quality of in-vitro studies and included disciplines other than dentistry [9].

In our sample, great variability in the distribution of scores across the AMSTAR-2 checklist items was recorded. Item 3, pertaining to the rationale used for selecting the study design received score of no in all included systematic reviews. We can hypothesize that the poor results in this item are due to the lack of importance or lack of awareness of the relevant methodological principles. Another explanation could be the limited applicability of this checklist item to a systematic review not involving clinical studies.

However, some items presented a high prevalence of scores *yes* or *py*. This was the case of item 5 where more than 2/3 of the systematic reviews, during the study selection process, applied unbiased approaches such as independent study selection and in duplicate. The absence of a similar study does not allow comparisons with our findings, but in another overview on reporting quality of in-vitro systematic reviews [9], the reported study selection process was often well reported.

Item 4, related to the literature search, received a *py* in more than 84% of the selected systematic reviews. This *py* score means that systematic review authors searched for literature in at least two major databases and provided information on keywords and/or search strategies [7]. However, for this item to receive a score of *yes*, five additional criteria should be met [7]; a requirement often hard to fulfill even for clinical systematic reviews.

The majority (81,6%) of the in-vitro experiments in this sample belonged to the specialties of restorative, prosthetic dentistry, and endodontics, and more than half dealt with dental materials.

More than 50% of the included systematic reviews did not present a meta-analysis due to the lack of homogeneity across individual studies; a common finding in clinical [10, 11], animal [12], and in-vitro [13, 14] experiments. More than one-third of the systematic reviews of this sample did not provide a satisfactory explanation and/or any discussion on the observed heterogeneity in the results of the review, as suggested by item 14 from the AMSTAR-2 checklist. For example, authors should discuss whether the randomization (or lack of) procedures had any impact on the results, or whether differences in the technical procedures among in-vitro experiments had any impact on the treatment effects and heterogeneity of the results.

The present data suggest that the methodological quality of systematic reviews of in-vitro dental studies reviews is suboptimal but with improvements over time. These findings might be explained by the greater awareness of the methodological aspects of research in more recent years, for example through the EQUATOR Network, [15], Cochrane [16], and the Campbell Collaboration [17].

In terms of the confidence in the results, the majority (68%) of systematic reviews was rated as “critically low”. This means that these reviews had at least two critical flaws in the AMSTAR-2 critical domains. Our results are in an agreement with a study that assessed 58 systematic reviews about cognitive behavioral therapy in psychiatric disorders and found that 72% of the systematic reviews were of critically low overall quality [18]. In the present sample, some systematic reviews were rated as low or moderate, but these ratings may be overoptimistic as we did not distinguish between *y* and *py* scores when determining the confidence. The rating py means that the item was only partially met, and it could be argued that merging py with y is problematic. Furthermore, we did not consider the number of non-critical flaws to rating down from moderate to low confidence. The AMSTAR-2 criteria recommend moving the overall appraisal down from moderate to low confidence when multiple non-critical weaknesses are present.

The critical domain which received large numbers of negative answers was that related to the discussion and interpretation of the potential effect of RoB on the findings of the review. A possible explanation for this poor performance is the scarce number of methodological tools to evaluate in-vitro experiments [19]. A second explanation is possibly the lack of awareness on the importance of evaluating the methodological quality of in-vitro research.

Regression analysis indicated an association between AMSTAR-2 scores, publication year, and dental specialty. More recent systematic reviews received higher AMSTAR-2 scores possibly due to methodological developments and awareness about the importance of adherence to the methodological guidance. The association between specialty and AMSTAR-2 scores is nevertheless difficult to explain. AMSTAR-2 percent scores varied across specialties overall with endodontics achieving the largest score. Endodontics had on average an 8.97% higher AMSTAR-2 score, compared to orthodontics with a range from -0.15% to 18.08%, a borderline significant finding. Periodontology had higher AMSTAR-2 scores compared to orthodontics. In this study, no association was found between IF and AMSTAR-2 percent scores; this finding does not corroborate with clinical systematic reviews published in high-impact factor clinical journals [20].

The present study has some limitations. Only systematic reviews published in English were included, and therefore some publication bias might be expected. However, we feel that the language limitation is unlikely to have any impact on the representativeness of the sample of systematic reviews included given that the great majority of PubMed indexed articles are published in English [21]. Furthermore, the original AMSTAR-2 tool was not designed to evaluate in-vitro experiments, and although most original items are still applicable, adaptations in some of the checklist sub-items were necessary. For example, in item 2, we excluded the need for a published protocol for in-vitro experiments since a database for in-vitro studies, like for clinical trials [22], does not seem to exist. Thus, it would be unfair to rate systematic reviews of in-vitro experiments using the same criterion used to evaluate systematic reviews of clinical studies. One can argue that the AMSTAR-2 checklist cannot be applied to systematic reviews of non-clinical studies. However, the core methodology of systematic reviews is similar to all levels of evidence. For example, a systematic review of animal or in-vitro experiments is also sensitive to publication bias or to the statistical approach used to conduct the meta-analysis.

This study has also some strengths. This is the first study to address the methodological quality of systematic review of in-vitro studies, includes a relatively large number of representative studies, and provides information on the association between methodological rigor and review characteristics.

Registries such as the PROSPERO database for systematic reviews of clinical studies in health and social care [23] and the Systematic Review Facility (SyRF) for in-vivo pre-clinical studies [24] exclude in-vitro studies. Registries for protocols of systematic reviews of in-vitro studies could promote unnecessary duplication, improvements in methodology, and reduce research waste [25]. Some improvements might also be necessary for the AMSTAR-2 methodology in reaching consensus in non-critical and critical domains. Some evidence suggests that there is variability among authors in the way the overall rating is derived when applying AMSTAR-2 [26]. In our assessment, we strictly followed the instructions of the AMSTAR-2 guideline to derive the overall rating. It appears that AMSTAR-2 is too rigid, but we feel that it can be further optimized to better distinguish among the different quality levels of the appraised systematic reviews.

We suggest that authors use the AMSTAR-2 checklist as a reference for planning and conducting systematic reviews of in-vitro studies. Although AMSTAR-2 was originally developed for assessing systematic reviews of clinical research, many of its items can also be applied to in-vitro systematic reviews. Further research is needed to fully validate this approach and optimize this checklist specifically for in-vitro studies.

In conclusion, the present study identified domains of systematic reviews of in-vitro dental studies that could be improved regarding their methodological quality. Year of publication of the systematic review and specialty were significant predictors of methodological quality. The overall rating of the confidence in the results was low and critically low for most systematic reviews.

## Supplementary Information

**Additional file 1. **

## Data Availability

The methodological quality assessment of the systematic reviews is reported in the supplementary file.
