# Supplementary material for: Methodological assessment of systematic reviews of in-vitro dental studies
Source: BMC Med Res Methodol. 2022 Apr 13;22:110. doi: 10.1186/s12874-022-01575-z (PMC9006561; doi:10.1186/s12874-022-01575-z)
Supplement: Supplementary file 1 — Additional file 1. [file 12874_2022_1575_MOESM1_ESM.pdf]

**Supplementary file**

**Additional file 1**. Search strategy in PubMed, Scopus and Web of Science

**PubMed**
#3 Add Search (“in vitro” OR in-vitro) Sort by: PublicationDate Filters: Systematic Reviews; Dental journals 217 02:43:48
#2 Add Search (“in vitro” OR in-vitro) Sort by: PublicationDate Filters: Systematic Reviews 1830 02:43:00
#1 Add Search (“in vitro” OR in-vitro) Sort by: PublicationDate 1625315 02:42:44

**Scopus**

4 ( TITLE-ABS-KEY ( "in vitro" OR in-vitro ) ) AND ( TITLE ( "systematic review" ) ) AND ( LIMIT-TO ( SUBJAREA , "DENT" ) )
211 document results

3 ( TITLE-ABS-KEY ( "in vitro" OR in-vitro ) ) AND ( TITLE ( "systematic review" ) )
1,473 document results

2 TITLE ( "systematic review" )
123,035 document results

1 TITLE-ABS-KEY ( "in vitro" OR in-vitro )
2,197,383 document results

**Web of Science**

# 4 265
#2 AND #1
Refined by: RESEARCH AREAS: ( DENTISTRY ORAL SURGERY MEDICINE )
Indexes=SCI-EXPANDED, SSCI, A&HCI, ESCI Timespan=All years

# 3 1,845
#2 AND #1
Indexes=SCI-EXPANDED, SSCI, A&HCI, ESCI Timespan=All years
Edit

# 2 124,610
TITLE: ("systematic review")
Indexes=SCI-EXPANDED, SSCI, A&HCI, ESCI Timespan=All years
Edit

# 1 1,387,765
TOPIC: (“in vitro” OR in-vitro)
Indexes=SCI-EXPANDED, SSCI, A&HCI, ESCI Timespan=All years

Updated search strategy on 06 January 2022

**Scopus**

5 ( TITLE-ABS-KEY ( "in vitro" OR in-vitro ) ) AND ( TITLE ( "systematic review" ) ) AND ( LIMIT-TO ( SUBJAREA , "dent" ) ) AND ( LIMIT-TO ( PUBYEAR , 2022 ) OR LIMIT-TO ( PUBYEAR , 2021 ) )
94 results

4 ( TITLE-ABS-KEY ( "in vitro" OR in-vitro ) ) AND ( TITLE ( "systematic review" ) ) AND ( LIMIT-TO ( SUBJAREA , "dent" ) )
349 results

3 ( TITLE-ABS-KEY ( "in vitro" OR in-vitro ) ) AND ( TITLE ( "systematic review" ) )
2,478 results

2 TITLE ( "systematic review" )
186,970 results

1 TITLE-ABS-KEY ( "in vitro" OR in-vitro )
2,384,265 results

**Web of Science**

6 #2 AND #1 and Dentistry Oral Surgery Medicine (Research Areas) and 2021 or 2020 (Publication Years)
121

5 #2 AND #1 and Dentistry Oral Surgery Medicine (Research Areas)
386

4 #2 AND #1
2,985

3 #2 AND #1

2,985
2 "systematic review" (Title)
190,047

1 “in vitro” OR in-vitro (All Fields)
1,575,951

**Additional file 2**. Criteria used to rate overall confidence in the results of the review (Shea et al. 2017)

| Confidence | Rationale |
|---|---|
| High | • *No or one non-critical weakness*: the systematic review provides an accurate and comprehensive summary of the results of the available studies that address the question of interest |
| Moderate | • *More than one non-critical weakness**: the systematic review has more than one weakness but no critical flaws. It may provide an accurate summary of the results of the available studies that were included in the review |
| Low | • *One critical flaw with or without non-critical weaknesses*: the review has a critical flaw and may not provide an accurate and comprehensive summary of the available studies that address the question of interest |
| Critical low | • *More than one critical flaw with or without non-critical weaknesses*: the review has more than one critical flaw and should not be relied on to provide an accurate and comprehensive summary of the available studies |

*Multiple non-critical weaknesses may diminish confidence in the review and it may be appropriate to move the overall appraisal down from moderate to low confidence.

**Additional file 3**. Rationale used in the assessment of in-vitro systematic reviews with AMSTAR-2

1. With reference to Item 1 we deleted “Timeframe for follow-up“.
2. In Item 2 we deleted “justification for any deviations from the protocol“.
3. In Item 4 we deleted “searched trial/study registries“ and “included/consulted content experts in the field“. For “Partial Yes” at least three sub-items should be reported.
4. In Item 8 we deleted the whole section “For yes, should also have ALL the following”. For “Partial Yes” at least two sub-items should be reported.
5. Regarding Item 9 the second sub-item was changed to: “lack of blinding of assessors when assessing outcomes”. Hence “patients and” was deleted.
6. Concerning Item 14 we deleted the first part of the second sub-item. Hence the sub-item was changed from “OR if heterogeneity was present the authors performed an investigation of sources of any heterogeneity in the results and discussed the impact of this on the results of the review” to “OR discussed the impact of this on the results of the review.”

**Additional file 4**. List of included systematic reviews of in-vitro dental studies

1. Abduo J, Lyons K, Bennani V, Waddell N, Swain M. Fit of screw-retained fixed implant frameworks fabricated by different methods: a systematic review. Int J Prosthodont. 2011;24:207–20.
2. Adnan S, Lone MM, Khan FR, Hussain SM, Nagi SE. Which is the most recommended medium for the storage and transport of avulsed teeth? A systematic review. Dent Traumatol. 2018;34:59–70. doi:10.1111/edt.12382.
3. Ahn S-Y, Kim H-C, Kim E. Kinematic Effects of Nickel-Titanium Instruments with Reciprocating or Continuous Rotation Motion: A Systematic Review of In Vitro Studies. J Endod. 2016;42:1009–17. doi:10.1016/j.joen.2016.04.002.
4. Almeida CM de, da Rosa WLO, Meereis CTW, Almeida SM de, Ribeiro JS, da Silva AF, Lund RG. Efficacy of antimicrobial agents incorporated in orthodontic bonding systems: a systematic review and meta-analysis. J Orthod. 2018;45:79–93. doi:10.1080/14653125.2018.1443872.
5. Almoudi MM, Hussein AS, Abu Hassan MI, Mohamad Zain N. A systematic review on antibacterial activity of zinc against Streptococcus mutans. Saudi Dent J. 2018;30:283–91. doi:10.1016/j.sdentj.2018.06.003.
6. AlShwaimi E, Bogari D, Ajaj R, Al-Shahrani S, Almas K, Majeed A. In Vitro Antimicrobial Effectiveness of Root Canal Sealers against Enterococcus faecalis: A Systematic Review. J Endod. 2016;42:1588–97. doi:10.1016/j.joen.2016.08.001.
7. Altmann ASP, Collares FM, Leitune VCB, Samuel SMW. The effect of antimicrobial agents on bond strength of orthodontic adhesives: a meta-analysis of in vitro studies. Orthodontics & craniofacial research. 2016;19:1–9. doi:10.1111/ocr.12100.
8. Altmann ASP, Leitune VCB, Collares FM. Influence of Eugenol-based Sealers on Push-out Bond Strength of Fiber Post Luted with Resin Cement: Systematic Review and Meta-analysis. J Endod. 2015;41:1418–23. doi:10.1016/j.joen.2015.05.014.
9. Andrade Lima Chaves C de, Machado AL, Vergani CE, Souza RF de, Giampaolo ET. Cytotoxicity of denture base and hard chairside reline materials: A systematic review. J Prosthet Dent. 2012;107:114–27. doi:10.1016/S0022-3913(12)60037-7.
10. Archambault A, Lacoursiere R, Badawi H, Major PW, Carey J, Flores-Mir C. Torque expression in stainless steel orthodontic brackets. A systematic review. Angle Orthod. 2010;80:201–10. doi:10.2319/080508-352.1.
11. Aurélio IL, Marchionatti AME, Montagner AF, May LG, Soares FZM. Does air particle abrasion affect the flexural strength and phase transformation of Y-TZP? A systematic review and meta-analysis. Dent Mater. 2016;32:827–45. doi:10.1016/j.dental.2016.03.021.
12. Bächle M, Kohal RJ. A systematic review of the influence of different titanium surfaces on proliferation, differentiation and protein synthesis of osteoblast-like MG63 cells. Clin Oral Implants Res. 2004;15:683–92. doi:10.1111/j.1600-0501.2004.01054.x.
13. Baumgartner S, Koletsi D, Verna C, Eliades T. The Effect of Enamel Sandblasting on Enhancing Bond Strength of Orthodontic Brackets: A Systematic Review and Meta-analysis. J Adhes Dent. 2017;19:463–73. doi:10.3290/j.jad.a39279.
14. Behring J, Junker R, Walboomers XF, Chessnut B, Jansen JA. Toward guided tissue and bone regeneration: morphology, attachment, proliferation, and migration of cells cultured on collagen barrier membranes. A systematic review. Odontology. 2008;96:1–11. doi:10.1007/s10266-008-0087-y.

15. Bernades KdO, Hilgert LA, Ribeiro APD, Garcia FCP, Pereira PNR. The influence of hemostatic agents on dentin and enamel surfaces and dental bonding: a systematic review. J Am Dent Assoc. 2014;145:1120–8. doi:10.14219/jada.2014.84.
16. Bohrer TC, Fontana PE, Lenzi TL, Soares FZM, Rocha RdO. Can Endodontic Irrigating Solutions Influence the Bond Strength of Adhesives to Coronal Dental Substrates? A Systematic Review and Meta-Analysis of In Vitro Studies. J Adhes Dent. 2018;20:481–94. doi:10.3290/j.jad.a41633.
17. Caldas IP, Alves GG, Barbosa IB, Scelza P, Noronha F de, Scelza MZ. In vitro cytotoxicity of dental adhesives: A systematic review. Dent Mater. 2019;35:195–205. doi:10.1016/j.dental.2018.11.028.
18. Carvalho MFF de, Leijôto-Lannes ACN, Rodrigues MCN de, Nogueira LC, Ferraz NKL, Moreira AN, et al. Viability of Bovine Teeth as a Substrate in Bond Strength Tests: A Systematic Review and Meta-analysis. J Adhes Dent. 2018;20:471–9. doi:10.3290/j.jad.a41636.
19. Collares FM, Portella FF, Rodrigues SB, Celeste RK, Leitune VCB, Samuel SMW. The influence of methodological variables on the push-out resistance to dislodgement of root filling materials: a meta-regression analysis. Int Endod J. 2016;49:836–49. doi:10.1111/iej.12539.
20. Collares FM, Rodrigues SB, Leitune VC, Celeste RK, Borba de Araújo F, Samuel SM. Chlorhexidine application in adhesive procedures: a meta-regression analysis. J Adhes Dent. 2013;15:11–8. doi:10.3290/j.jad.a28732.
21. Coray R, Zeltner M, Özcan M. Fracture strength of implant abutments after fatigue testing: A systematic review and a meta-analysis. J Mech Behav Biomed Mater. 2016;62:333–46. doi:10.1016/j.jmbbm.2016.05.011.
22. Corrêa G, Pradebon Brondani L, Sarkis-Onofre R, Dalmolin Bergoli C. Restorative strategies for weakened roots: Systematic review and Meta-analysis of in vitro studies. BDS. 2019;22:Process. doi:10.14295/bds.2019.v22i1.1672.
23. Cuevas-Suárez CE, Da Rosa WLdO, Lund RG, Da Silva AF, Piva E. Bonding Performance of Universal Adhesives: An Updated Systematic Review and Meta-Analysis. J Adhes Dent. 2019;21:7–26. doi:10.3290/j.jad.a41975.
24. Cury S-E-N, Aliaga-Del Castillo A, Pinzan A, Sakoda K-L, Bellini-Pereira S-A, Janson G. Orthodontic brackets friction changes after clinical use: A systematic review. J Clin Exp Dent. 2019;11:e482-e490. doi:10.4317/jced.55676.
25. Davoudi A, Rismanchian M. Effects of modifying implant screw access channels on the amount of extruded excess cement and retention of cement-retained implant-supported dental prostheses: A systematic review. J Prosthet Dent. 2019;121:52–8. doi:10.1016/j.prosdent.2018.03.002.
26. Dumbryte I, Vebriene J, Linkeviciene L, Malinauskas M. Enamel microcracks in the form of tooth damage during orthodontic debonding: a systematic review and meta-analysis of in vitro studies. Eur J Orthod. 2018;40:636–48. doi:10.1093/ejo/cjx102.
27. Ehsani S, Mandich M-A, El-Bialy TH, Flores-Mir C. Frictional resistance in self-ligating orthodontic brackets and conventionally ligated brackets. A systematic review. Angle Orthod. 2009;79:592–601. doi:10.2319/060208-288.1.
28. Ekambaram M, Yiu CKY, Matinlinna JP. Bonding of resin adhesives to caries-affected dentin – A systematic review. International Journal of Adhesion and Adhesives. 2015;61:23–34. doi:10.1016/j.ijadhadh.2015.04.011.

29. Ferreira F, Adeodato C, Barbosa I, Aboud L, Scelza P, Zaccaro Scelza M. Movement kinematics and cyclic fatigue of NiTi rotary instruments: a systematic review. Int Endod J. 2017;50:143–52. doi:10.1111/iej.12613.
30. Finnema KJ, Ozcan M, Post WJ, Ren Y, Dijkstra PU. In-vitro orthodontic bond strength testing: a systematic review and meta-analysis. Am J Orthod Dentofacial Orthop. 2010;137:615-622.e3. doi:10.1016/j.ajodo.2009.12.021.
31. García-Sanz V, Paredes-Gallardo V, Mendoza-Yero O, Carbonell-Leal M, Albaladejo A, Montiel-Company JM, Bellot-Arcís C. The effects of lasers on bond strength to ceramic materials: A systematic review and meta-analysis. PLoS One. 2018;13:e0190736. doi:10.1371/journal.pone.0190736.
32. Gorman CM, Ray NJ, Burke FM. The effect of endodontic access on all-ceramic crowns: A systematic review of in vitro studies. J Dent. 2016;53:22–9. doi:10.1016/j.jdent.2016.08.005.
33. Goujat A, Abouelleil H, Colon P, Jeannin C, Pradelle N, Seux D, Grosgogeat B. Marginal and internal fit of CAD-CAM inlay/onlay restorations: A systematic review of in vitro studies. J Prosthet Dent. 2019;121:590-597.e3. doi:10.1016/j.prosdent.2018.06.006.
34. Grewal Bach GK, Torrealba Y, Lagravère MO. Orthodontic bonding to porcelain: a systematic review. Angle Orthod. 2014;84:555–60. doi:10.2319/083013-636.1.
35. Holliday RS, Campbell J, Preshaw PM. Effect of nicotine on human gingival, periodontal ligament and oral epithelial cells. A systematic review of the literature. J Dent. 2019;86:81–8. doi:10.1016/j.jdent.2019.05.030.
36. Iliadi A, Koletsi D, Eliades T. Forces and moments generated by aligner-type appliances for orthodontic tooth movement: A systematic review and meta-analysis. Orthodontics & craniofacial research. 2019;22:248–58. doi:10.1111/ocr.12333.
37. Isolan CP, Sarkis-Onofre R, Moraes RR. Bonding to sound and caries-affected dentin: Systematic review and meta-analysis. Dental Materials. 2015;31:e55. doi:10.1016/j.dental.2015.08.122.
38. Kaczor K, Gerula-Szymańska A, Smektała T, Safranow K, Lewusz K, Nowicka A. Effects of different etching modes on the nanoleakage of universal adhesives: A systematic review and meta-analysis. Journal of esthetic and restorative dentistry : official publication of the American Academy of Esthetic Dentistry … [et al.]. 2018;30:287–98. doi:10.1111/jerd.12375.
39. Kaizer MR, Oliveira-Ogliari A de, Cenci MS, Opdam NJM, Moraes RR. Do nanofill or submicron composites show improved smoothness and gloss? A systematic review of in vitro studies. Dent Mater. 2014;30:e41-78. doi:10.1016/j.dental.2014.01.001.
40. Kaufmann M, Lenherr P, Walter C, Thurnheer T, Attin T, Wiedemeier DB, Schmidlin PR. Comparing the Antimicrobial In Vitro Efficacy of Amoxicillin/Metronidazole against Azithromycin-A Systematic Review. Dent J (Basel) 2018. doi:10.3390/dj6040059.
41. Kim J-H, Kim KR, Kim S. Critical appraisal of implant impression accuracies: A systematic review. J Prosthet Dent. 2015;114:185-92.e1. doi:10.1016/j.prosdent.2015.02.005.
42. Kuik K, Ruiter MHT de, Lange J de, Hoekema A. Fixation methods in sagittal split ramus osteotomy: a systematic review on in vitro biomechanical assessments. Int J Oral Maxillofac Surg. 2019;48:56–70. doi:10.1016/j.ijom.2018.06.013.
43. Laaksonen M, Sorsa T, Salo T. Emdogain in carcinogenesis: a systematic review of in vitro studies. J Oral Sci. 2010;52:1–11. doi:10.2334/josnusd.52.1.
44. Lee H, So JS, Hochstedler JL, Ercoli C. The accuracy of implant impressions: A systematic review. J Prosthet Dent. 2008;100:285–91. doi:10.1016/S0022-3913(08)60208-5.

45. Lenzi TL, Gimenez T, Tedesco TK, Mendes FM, Rocha RdO, Raggio DP. Adhesive systems for restoring primary teeth: a systematic review and meta-analysis of in vitro studies. Int J Paediatr Dent. 2016;26:364–75. doi:10.1111/ipd.12210.
46. Lo Russo L, Caradonna G, Biancardino M, Lillo A de, Troiano G, Guida L. Digital versus conventional workflow for the fabrication of multiunit fixed prostheses: A systematic review and meta-analysis of vertical marginal fit in controlled in vitro studies. J Prosthet Dent. 2019;122:435–40. doi:10.1016/j.prosdent.2018.12.001.
47. Lombardo G, Pagano S, Cianetti S, Capobianco B, Orso M, Negri P, et al. Sub-ablative laser irradiation to prevent acid demineralisation of dental enamel. A systematic review of literature reporting in vitro studies. Eur J Paediatr Dent. 2019;20:295–301. doi:10.23804/ejpd.2019.20.04.07.
48. Louropoulou A, Slot DE, van der Weijden F. Influence of mechanical instruments on the biocompatibility of titanium dental implants surfaces: a systematic review. Clin Oral Implants Res. 2015;26:841–50. doi:10.1111/clr.12365.
49. Marchionatti AME, Aurélio IL, May LG. Does veneering technique affect the flexural strength or load-to-failure of bilayer Y-TZP? A systematic review and meta-analysis. J Prosthet Dent. 2018;119:916–24. doi:10.1016/j.prosdent.2017.11.013.
50. Martins FV, Vasques WF, Fonseca EM. Evaluation of the efficiency of fluoride-releasing adhesives for preventing secondary caries in-vitro: a systematic review and meta-analysis. Eur Arch Paediatr Dent. 2019;20:1–8. doi:10.1007/s40368-018-0388-y.
51. Martins FV, Vasques WF, Fonseca EM. How the Variations of the Thickness in Ceramic Restorations of Lithium Disilicate and the Use of Different Photopolymerizers Influence the Degree of Conversion of the Resin Cements: A Systematic Review and Meta-Analysis. J Prosthodont. 2019;28:e395-e403. doi:10.1111/jopr.12920.
52. Masarwa N, Mohamed A, Abou-Rabii I, Abu Zaghlan R, Steier L. Longevity of Self-etch Dentin Bonding Adhesives Compared to Etch-and-rinse Dentin Bonding Adhesives: A Systematic Review. J Evid Based Dent Pract. 2016;16:96–106. doi:10.1016/j.jebdp.2016.03.003.
53. Mello CC de, Bitencourt SB, dos Santos DM, Pesqueira AA, Pellizzer EP, Goiato MC. The Effect of Surface Treatment on Shear Bond Strength between Y-TZP and Veneer Ceramic: A Systematic Review and Meta-Analysis. J Prosthodont. 2018;27:624–35. doi:10.1111/jopr.12727.
54. Mello CC, Lemos CAA, Luna Gomes JM de, Verri FR, Pellizzer EP. CAD/CAM vs Conventional Technique for Fabrication of Implant-Supported Frameworks: A Systematic Review and Meta-analysis of In Vitro Studies. Int J Prosthodont. 2019;32:182–92. doi:10.11607/ijp.5616.
55. Menezes-Silva R, Cabral RN, Pascotto RC, Borges AFS, Martins CC, Navarro MFdL, et al. Mechanical and optical properties of conventional restorative glass-ionomer cements - a systematic review. J Appl Oral Sci. 2019;27:e2018357. doi:10.1590/1678-7757-2018-0357.
56. Moharrami M, Perrotti V, Iaculli F, Love RM, Quaranta A. Effects of air abrasive decontamination on titanium surfaces: A systematic review of in vitro studies. Clinical implant dentistry and related research. 2019;21:398–421. doi:10.1111/cid.12747.
57. Moraes AP, Sarkis-Onofre R, Moraes RR, Cenci MS, Soares CJ, Pereira-Cenci T. Can Silanization Increase the Retention of Glass-fiber posts? A Systematic Review and Meta-analysis of In Vitro Studies. Operative dentistry. 2015;40:567–80. doi:10.2341/14-330-O.
58. Moreira AHJ, Rodrigues NF, Pinho ACM, Fonseca JC, Vilaça JL. Accuracy Comparison of Implant Impression Techniques: A Systematic Review. Clinical implant dentistry and related research. 2015;17 Suppl 2:e751-64. doi:10.1111/cid.12310.

59. Możyńska J, Metlerski M, Lipski M, Nowicka A. Tooth Discoloration Induced by Different Calcium Silicate-based Cements: A Systematic Review of In Vitro Studies. J Endod. 2017;43:1593–601. doi:10.1016/j.joen.2017.04.002.
60. Nagendrababu V, Jayaraman J, Suresh A, Kalyanasundaram S, Neelakantan P. Effectiveness of ultrasonically activated irrigation on root canal disinfection: a systematic review of in vitro studies. Clin Oral Investig. 2018;22:655–70. doi:10.1007/s00784-018-2345-x.
61. Nassar U, Aziz T, Flores-Mir C. Dimensional stability of irreversible hydrocolloid impression materials as a function of pouring time: A systematic review. J Prosthet Dent. 2011;106:126–33. doi:10.1016/S0022-3913(11)60108-X.
62. Nawafleh N, Hatamleh M, Elshiyab S, Mack F. Lithium Disilicate Restorations Fatigue Testing Parameters: A Systematic Review. J Prosthodont. 2016;25:116–26. doi:10.1111/jopr.12376.
63. Neelakantan P, Ahmed HMA, Wong MCM, Matinlinna JP, Cheung GSP. Effect of root canal irrigation protocols on the dislocation resistance of mineral trioxide aggregate-based materials: A systematic review of laboratory studies. Int Endod J. 2018;51:847–61. doi:10.1111/iej.12898.
64. Ntrouka VI, Slot DE, Louropoulou A, van der Weijden F. The effect of chemotherapeutic agents on contaminated titanium surfaces: a systematic review. Clin Oral Implants Res. 2011;22:681–90. doi:10.1111/j.1600-0501.2010.02037.x.
65. Oliveira LV, Maia TS, Zancopé K, Menezes MdS, Soares CJ, Moura CCG. Can intra-radicular cleaning protocols increase the retention of fiberglass posts? A systematic review. Braz Oral Res. 2018;32:e16. doi:10.1590/1807-3107bor-2018.vol32.0016.
66. Oliveira LB, Massignan C, Oenning AC, Rovaris K, Bolan M, Porporatti AL, Luca Canto G de. Validity of micro-CT for in vitro caries detection: a systematic review and meta-analysis. Dentomaxillofac Radiol. 2020;49:20190347. doi:10.1259/dmfr.20190347.
67. Osmanovic A, Halilovic S, Kurtovic-Kozaric A, Hadziabdic N. Evaluation of periodontal ligament cell viability in different storage media based on human PDL cell culture experiments-A systematic review. Dent Traumatol. 2018;34:384–93. doi:10.1111/edt.12437.
68. Özcan M, Bernasconi M. Adhesion to zirconia used for dental restorations: a systematic review and meta-analysis. J Adhes Dent. 2015;17:7–26. doi:10.3290/j.jad.a33525.
69. Özcan M, Höhn J, Moura DD, Souza R. Influence of testing parameters on the load-bearing capacity of prosthetic materials used for fixed dental prosthesis: A systematic review and meta-analysis. BDS. 2018;21:470. doi:10.14295/bds.2018.v21i4.1652.
70. Özcan M, Jonasch M. Effect of Cyclic Fatigue Tests on Aging and Their Translational Implications for Survival of All-Ceramic Tooth-Borne Single Crowns and Fixed Dental Prostheses. J Prosthodont. 2018;27:364–75. doi:10.1111/jopr.12566.
71. Papia E, Larsson C, Du Toit M, Vult von Steyern P. Bonding between oxide ceramics and adhesive cement systems: a systematic review. J Biomed Mater Res B Appl Biomater. 2014;102:395–413. doi:10.1002/jbm.b.33013.
72. Pardal-Peláez B, Montero J. Preload loss of abutment screws after dynamic fatigue in single implant-supported restorations. A systematic review. J Clin Exp Dent. 2017;9:e1355-e1361. doi:10.4317/jced.54374.
73. Parikh M, Kishan KV, Solanki NP, Parikh M, Savaliya K, Bindu VH, Devika TD. Efficacy of Removal of Calcium Hydroxide Medicament from Root Canals by Endoactivator and Endovac Irrigation Techniques: A Systematic Review of In vitro Studies. Contemp Clin Dent. 2019;10:135–42. doi:10.4103/ccd.ccd_335_18.

74. Passos SP, Nychka JA, Major P, Linke B, Flores-Mir C. In vitro fracture toughness of commercial Y-TZP ceramics: a systematic review. J Prosthodont. 2015;24:1–11. doi:10.1111/jopr.12179.
75. Passos SP, Torrealba Y, Major P, Linke B, Flores-Mir C, Nychka JA. In vitro wear behavior of zirconia opposing enamel: a systematic review. J Prosthodont. 2014;23:593–601. doi:10.1111/jopr.12167.
76. Perroni AP, Kaizer MR, Della Bona A, Moraes RR, Boscato N. Influence of light-cured luting agents and associated factors on the color of ceramic laminate veneers: A systematic review of in vitro studies. Dent Mater. 2018;34:1610–24. doi:10.1016/j.dental.2018.08.298.
77. Pinho M, Manso M, Martin C, Souza J, Almeida R, Ferreira A. Adhesion strength of orthodontic brackets to acrylic surfaces. A systematic review on in vitro studies. j.rpemd 2017. doi:10.24873/j.rpemd.2017.07.020.
78. Pires CW, Soldera EB, Bonzanini LIL, Lenzi TL, Soares FZM, Montagner AF, Rocha RdO. Is Adhesive Bond Strength Similar in Primary and Permanent Teeth? A Systematic Review and Meta-analysis. J Adhes Dent. 2018;20:87–97. doi:10.3290/j.jad.a40296.
79. Reis AF, Vestphal M, Amaral RCd, Rodrigues JA, Roulet J-F, Roscoe MG. Efficiency of polymerization of bulk-fill composite resins: a systematic review. Braz Oral Res. 2017;31:e59. doi:10.1590/1807-3107BOR-2017.vol31.0059.
80. Resende KKM, Faria GP, Longo DL, Martins LJO, Costa CRR. In vitro evaluation of plants as storage media for avulsed teeth: A systematic review. Dent Traumatol. 2020;36:3–18. doi:10.1111/edt.12501.
81. Rodrigues CdS, Aurélio IL, Kaizer MdR, Zhang Y, May LG. Do thermal treatments affect the mechanical behavior of porcelain-veneered zirconia? A systematic review and meta-analysis. Dent Mater. 2019;35:807–17. doi:10.1016/j.dental.2019.02.016.
82. Rosa WLdOd, Piva E, Silva AFd. Bond strength of universal adhesives: A systematic review and meta-analysis. J Dent. 2015;43:765–76. doi:10.1016/j.jdent.2015.04.003.
83. Samiei M, Shirazi S., Azar F.P., Fathifar Z., Ghojazadeh M., Alipour M. The Effect of Different Mixing Methods on the Properties of Calcium-enriched Mixture Cement: A Systematic Review of in Vitro Studies. 1. 2019;14:240–6. doi:10.22037/iej.v14i4.25126.
84. Sarkis-Onofre R, Skupien JA, Cenci MS, Moraes RR, Pereira-Cenci T. The role of resin cement on bond strength of glass-fiber posts luted into root canals: a systematic review and meta-analysis of in vitro studies. Operative dentistry. 2014;39:E31-44. doi:10.2341/13-070-LIT.
85. Shahmiri R, Standard OC, Hart JN, Sorrell CC. Optical properties of zirconia ceramics for esthetic dental restorations: A systematic review. J Prosthet Dent. 2018;119:36–46. doi:10.1016/j.prosdent.2017.07.009.
86. Shahravan A, Haghdoost A-A, Adl A, Rahimi H, Shadifar F. Effect of smear layer on sealing ability of canal obturation: a systematic review and meta-analysis. J Endod. 2007;33:96–105. doi:10.1016/j.joen.2006.10.007.
87. Silva EJNL, Canabarro A, Andrade MRTC, Cavalcante DM, Stetten O von, Fidalgo TKdS, De-Deus G. Dislodgment Resistance of Bioceramic and Epoxy Sealers: A Systematic Review and Meta-analysis. J Evid Based Dent Pract. 2019;19:221–35. doi:10.1016/j.jebdp.2019.04.004.
88. Silva EJNL, Rover G, Belladonna FG, De-Deus G, da Silveira Teixeira C, da Silva Fidalgo TK. Impact of contracted endodontic cavities on fracture resistance of endodontically treated teeth: a systematic review of in vitro studies. Clin Oral Investig. 2018;22:109–18. doi:10.1007/s00784-017-2268-y.

89. Skupien JA, Sarkis-Onofre R, Cenci MS, Moraes RR de, Pereira-Cenci T. A systematic review of factors associated with the retention of glass fiber posts. Braz Oral Res 2015. doi:10.1590/1807-3107BOR-2015.vol29.0074.
90. Soares FZM, Follak A, da Rosa LS, Montagner AF, Lenzi TL, Rocha RO. Bovine tooth is a substitute for human tooth on bond strength studies: A systematic review and meta-analysis of in vitro studies. Dent Mater. 2016;32:1385–93. doi:10.1016/j.dental.2016.09.019.
91. Solanki NP, Venkappa KK, Shah NC. Biocompatibility and sealing ability of mineral trioxide aggregate and biodentine as root-end filling material: A systematic review. J Conserv Dent. 2018;21:10–5. doi:10.4103/JCD.JCD_45_17.
92. Sousa APBR de, França K, Lucas Rezende LVM de, do Nascimento Poubel DL, Almeida JCF, Toledo IP de, Garcia FCP. In vitro tooth reattachment techniques: A systematic review. Dent Traumatol. 2018;34:297–310. doi:10.1111/edt.12414.
93. Strauss F-J, Nasirzade J, Kargarpoor Z, Stähli A, Gruber R. Effect of platelet-rich fibrin on cell proliferation, migration, differentiation, inflammation, and osteoclastogenesis: a systematic review of in vitro studies. Clin Oral Investig. 2020;24:569–84. doi:10.1007/s00784-019-03156-9.
94. Tageldin H, Llano-Pérula MC de, Thevissen P, Celis J-P, Willems G. Resistance to Sliding in Orthodontics: A Systematic Review. Jacobs Journal of Dentistry and Research. 2016:1–32.
95. Taha AA, Patel MP, Hill RG, Fleming PS. The effect of bioactive glasses on enamel remineralization: A systematic review. J Dent. 2017;67:9–17. doi:10.1016/j.jdent.2017.09.007.
96. Tavares SJdO, Sarmento EB, Guimarães LdS, Antunes LAA, Antunes LS, Gomes CC. The influence of kinematics of engine-driven nickel-titanium instruments on root canal shape assessed by micro-computed tomography: a systematic review. Acta Odontol Scand. 2019;77:347–58. doi:10.1080/00016357.2019.1570331.
97. Theodosopoulou JN, Niederman R. A systematic review of in vitro retrograde obturation materials. J Endod. 2005;31:341–9. doi:10.1097/01.don.0000145034.10218.3f.
98. Ting M, Whitaker EJ, Albandar JM. Systematic review of the in vitro effects of statins on oral and perioral microorganisms. Eur J Oral Sci. 2016;124:4–10. doi:10.1111/eos.12239.
99. Tsesis I, Blazer T, Ben-Izhack G, Taschieri S, Del Fabbro M, Corbella S, Rosen E. The Precision of Electronic Apex Locators in Working Length Determination: A Systematic Review and Meta-analysis of the Literature. J Endod. 2015;41:1818–23. doi:10.1016/j.joen.2015.08.012.
100. Tzanakakis E-GC, Tzoutzas IG, Koidis PT. Is there a potential for durable adhesion to zirconia restorations? A systematic review. J Prosthet Dent. 2016;115:9–19. doi:10.1016/j.prosdent.2015.09.008.
101. Uzunoglu-Özyürek E, Küçükkaya Eren S, Karahan S. Effect of root canal sealers on the fracture resistance of endodontically treated teeth: a systematic review of in vitro studies. Clin Oral Investig. 2018;22:2475–85. doi:10.1007/s00784-018-2540-9.
102. Valente LL, Sarkis-Onofre R, Gonçalves AP, Fernández E, Loomans B, Moraes RR. Repair bond strength of dental composites: systematic review and meta-analysis. International Journal of Adhesion and Adhesives. 2016;69:15–26. doi:10.1016/j.ijadhadh.2016.03.020.
103. Virdee SS, Seymour DW, Farnell D, Bhamra G, Bhakta S. Efficacy of irrigant activation techniques in removing intracanal smear layer and debris from mature permanent teeth: a systematic review and meta-analysis. Int Endod J. 2018;51:605–21. doi:10.1111/iej.12877.

104. Western JS, Dicksit DD. A systematic review of randomized controlled trials on sterilization methods of extracted human teeth. J Conserv Dent. 2016;19:343–6. doi:10.4103/0972-0707.186457.
105. Yassen GH, Platt JA. The effect of nonsetting calcium hydroxide on root fracture and mechanical properties of radicular dentine: a systematic review. Int Endod J. 2013;46:112–8. doi:10.1111/j.1365-2591.2012.02121.x.
106. Yaylali IE, Kececi AD, Ureyen Kaya B. Ultrasonically Activated Irrigation to Remove Calcium Hydroxide from Apical Third of Human Root Canal System: A Systematic Review of In Vitro Studies. J Endod. 2015;41:1589–99. doi:10.1016/j.joen.2015.06.006.
107. Yu H, Chen Y-H, Cheng H, Sawase T. Finish-line designs for ceramic crowns: A systematic review and meta-analysis. J Prosthet Dent. 2019;122:22-30.e5. doi:10.1016/j.prosdent.2018.10.002.
108. Yu H, Özcan M, Yoshida K, Cheng H, Sawase T. Bonding to industrial indirect composite blocks: A systematic review and meta-analysis. Dent Mater. 2020;36:119–34. doi:10.1016/j.dental.2019.11.002.
109. Ahmed WM, Shariati B, Gazzaz AZ, Sayed ME, Carvalho RM. Fit of tooth-supported zirconia single crowns-A systematic review of the literature. Clin Exp Dent Res. 2020;6:700–16. doi:10.1002/cre2.323.
110. Alammar A, Att W. Bonding durability between zirconia and different types of tooth or implant abutments-a systematic review. Part I: outcomes of in vitro studies. Int J Prosthodont. 2021;34:650–69. doi:10.11607/ijp.6870.
111. Alhajj MN, Qi CH, Sayed ME, Johari Y, Ariffin Z. Fracture Resistance of Titanium and Fiber Dental Posts: A Systematic Review and Meta-Analysis. J Prosthodont 2021. doi:10.1111/jopr.13428.
112. Aljomard YRM, Altunok EÇ, Kara HB. Enamel wear against monolithic zirconia restorations: A meta-analysis and systematic review of in vitro studies. Journal of esthetic and restorative dentistry : official publication of the American Academy of Esthetic Dentistry … [et al.] 2021. doi:10.1111/jerd.12823.
113. Almeida Bastos N, Bitencourt SB, Carneiro RF, Ferrairo BM, Strelhow SSF, dos Santos DM, Bombonatti JFS. Marginal and internal adaptation of lithium disilicate partial restorations: A systematic review and meta-analysis. J Indian Prosthodont Soc. 2020;20:338–44. doi:10.4103/jips.jips_112_20.
114. Al-Thobity AM, Gad MM. Effect of silicon dioxide nanoparticles on the flexural strength of heat-polymerized acrylic denture base material: A systematic review and meta-analysis. Saudi Dent J. 2021;33:775–83. doi:10.1016/j.sdentj.2021.08.008.
115. Anita P, Kailasam V. Effect of sandblasting on the shear bond strength of recycled metal brackets: A systematic review and meta-analysis of in-vitro studies. Int Orthod. 2021;19:377–88. doi:10.1016/j.ortho.2021.05.007.
116. Astudillo-Rubio D, Delgado-Gaete A, Bellot-Arcís C, Montiel-Company JM, Pascual-Moscardó A, Almerich-Silla JM. Mechanical properties of provisional dental materials: A systematic review and meta-analysis. PLoS One. 2018;13:e0193162. doi:10.1371/journal.pone.0193162.
117. Baldion PA, Betancourt DE, Gutierrez DM, Beltran EO, Lafaurie GI, Chambrone L. Influence of endodontic irrigants on bond strength between glass-fibre posts and dentin: A systematic review of in vitro studies. International Journal of Adhesion and Adhesives. 2020;102:102685. doi:10.1016/j.ijadhadh.2020.102685.
118. Bangera MK, Kotian R, Madhyastha P. Effects of silver nanoparticle-based antimicrobial formulations on the properties of denture polymer: A systematic review and meta-analysis of in vitro studies. J Prosthet Dent 2021. doi:10.1016/j.prosdent.2021.05.011.

119. Bangera MK, Kotian R, N R. Effect of titanium dioxide nanoparticle reinforcement on flexural strength of denture base resin: A systematic review and meta-analysis. Jpn Dent Sci Rev. 2020;56:68–76. doi:10.1016/j.jdsr.2020.01.001.
120. Bitencourt SB, Ferreira LC, Mazza LC, dos Santos DM, Pesqueira AA, Theodoro LH. Effect of laser irradiation on bond strength between zirconia and resin cement or veneer ceramic: A systematic review and meta-analysis. J Indian Prosthodont Soc. 2021;21:125–37. doi:10.4103/jips.jips_590_20.
121. Bohrer TC, Fontana PE, Rocha RO, Kaizer OB. Post-Space Treatment Influences the Bond Strength In Endodontically Treated Teeth: A Systematic Review and Meta-Analysis of In Vitro Studies. Operative dentistry. 2021;46:E132-E157. doi:10.2341/19-277-LIT.
122. Bonzanini LIL, Cavalheiro CP, Scherer MM, Pedrotti D, Bottezini PA, Da Rosa RA, et al. Reciprocating and Rotatory NiTi Instruments Used for Root Canal Preparation of Primary Teeth: A Systematic Review and Meta-Analysis. Pesqui. Bras. Odontopediatria Clín. Integr. 2021. doi:10.1590/pboci.2021.124.
123. Carneiro Pereira AL, Medeiros VR, da Fonte Porto Carreiro A. Influence of implant position on the accuracy of intraoral scanning in fully edentulous arches: A systematic review. J Prosthet Dent. 2021;126:749–55. doi:10.1016/j.prosdent.2020.09.008.
124. Cavalcante-Leão BL, Araujo C-M de, Basso I-B, Schroder A-G-D, Guariza-Filho O, Ravazzi G-C, et al. Is there scientific evidence of the mouthwashes effectiveness in reducing viral load in Covid-19? A systematic review. J Clin Exp Dent. 2021;13:e179-e189. doi:10.4317/jced.57406.
125. Corvino E, Pesce P, Mura R, Marcano E, Canullo L. Influence of Modified Titanium Abutment Surface on Peri-implant Soft Tissue Behavior: A Systematic Review of In Vitro Studies. Int J Oral Maxillofac Implants. 2020;35:503–19. doi:10.11607/jomi.8110.
126. Costa RTF, Pellizzer EP, Vasconcelos BCdE, Gomes JML, Lemos CAA, Moraes SLD de. Surface roughness of acrylic resins used for denture base after chemical disinfection: A systematic review and meta-analysis. Gerodontology. 2021;38:242–51. doi:10.1111/ger.12529.
127. da Costa RMB, Venante HS, Pordeus MD, Chappuis-Chocano AP, Neppelenbroek KH, Santiago Júnior JF, Porto VC. Does microwave disinfection affect the dimensional stability of denture base acrylic resins? A systematic review. Gerodontology 2021. doi:10.1111/ger.12597.
128. David C, Cardoso de Cardoso G, Isolan CP, Piva E, Moraes RR, Cuevas-Suarez CE. Bond strength of self-adhesive flowable composite resins to dental tissues: A systematic review and meta-analysis of in vitro studies. J Prosthet Dent 2021. doi:10.1016/j.prosdent.2021.02.020.
129. Eswaramurthy P, Aras M, DSouza KM, Nagarsekar A, Gaunkar RB. Contemporary Sterilization Protocols of Healing Abutments for Reusability: A Systematic Review. JDR Clin Trans Res. 2021:23800844211045897. doi:10.1177/23800844211045897.
130. Fathy SM, Al-Zordk W, E Grawish M, V Swain M. Flexural strength and translucency characterization of aesthetic monolithic zirconia and relevance to clinical indications: A systematic review. Dent Mater. 2021;37:711–30. doi:10.1016/j.dental.2021.01.022.
131. Fernández-Barrera MÁ, Da Silva AF, Pontigo-Loyola AP, Zamarripa-Calderón JE, Piva E, Cuevas-Suárez CE. The Effect of Deproteinizing Agents on Bond Strength of Resin-based Materials to Enamel: A Systematic Review and Meta-Analysis of In Vitro Studies. J Adhes Dent. 2021;23:287–96. doi:10.3290/j.jad.b1649893.

132. Ferreira I, Braga AC, Pina-Vaz I. Effect of Gutta-percha Solvents on the Bond Strength of Sealers to Intraradicular Dentin: A Systematic Review. Iranian Endodontic Journal. 2021;16:17–25. doi:10.22037/iej.v16i1.29297.
133. Fröhlich TT, Gindri LD, Soares FZM, Oliveira Rocha R de. Does the etching strategy influence the bonding of universal adhesive systems to primary teeth? A systematic review and meta-analysis of in vitro studies. Eur Arch Paediatr Dent. 2021;22:1015–22. doi:10.1007/s40368-021-00639-w.
134. Fröhlich TT, Rocha RdO, Botton G. Does previous application of silver diammine fluoride influence the bond strength of glass ionomer cement and adhesive systems to dentin? Systematic review and meta-analysis. Int J Paediatr Dent. 2020;30:85–95. doi:10.1111/ipd.12571.
135. Gerula-Szymańska A, Kaczor K, Lewusz-Butkiewicz K, Nowicka A. Marginal integrity of flowable and packable bulk fill materials used for class II restorations -A systematic review and meta-analysis of in vitro studies. Dent Mater J. 2020;39:335–44. doi:10.4012/dmj.2018-180.
136. Golshah A, Bagheri N, Moslem Imani M, Safari-Faramani R. Effects of different types of laser etching versus phosphoric acid etching on shear bond strength of metal brackets to human enamel: A systematic review and meta-analysis of in vitro studies. Int Orthod. 2020;18:673–83. doi:10.1016/j.ortho.2020.10.001.
137. Iaculli F, Rengo C, Lodato V, Patini R, Spagnuolo G, Rengo S. Fracture resistance of endodontically-treated maxillary premolars restored with different type of posts and direct composite reconstructions: A systematic review and meta-analysis of in vitro studies. Dent Mater. 2021;37:e455-e484. doi:10.1016/j.dental.2021.06.007.
138. Jacob SE, Zubair SM, Thomas MS, Jathanna V, Shenoy R. Effect of surface treatment on the dislocation resistance of prefabricated esthetic fiber posts bonded with self-adhesive resin cement: A systematic review and meta-analysis. J Conserv Dent. 2021;24:113–23. doi:10.4103/jcd.jcd_656_20.
139. Janjic M, Docheva D, Trickovic Janjic O, Wichelhaus A, Baumert U. In Vitro Weight-Loaded Cell Models for Understanding Mechanodependent Molecular Pathways Involved in Orthodontic Tooth Movement: A Systematic Review. Stem Cells Int. 2018;2018:3208285. doi:10.1155/2018/3208285.
140. Jurema ALB, Filgueiras AT, Santos KA, Bresciani E, Caneppele TMF. Effect of intraradicular fiber post on the fracture resistance of endodontically treated and restored anterior teeth: A systematic review and meta-analysis. J Prosthet Dent 2021. doi:10.1016/j.prosdent.2020.12.013.
141. Kreve S, Cândido Dos Reis A. Influence of the electrostatic condition of the titanium surface on bacterial adhesion: A systematic review. J Prosthet Dent. 2021;125:416–20. doi:10.1016/j.prosdent.2020.02.003.
142. Kwon SR, Cortez E, Wang M, Jagwani M, Oyoyo U, Li Y. Systematic review of in vitro studies evaluating tooth bleaching efficacy. Am J Dent. 2020;33:17–24.
143. Li J, Hua F, Xu P, Huang C, Yang H. Effects of Desensitizers on Adhesive-Dentin Bond Strength: A Systematic Review and Meta-analysis. J Adhes Dent. 2021;23:7–19. doi:10.3290/j.jad.b916811.
144. Li M, Zhang C, Yang Y. Effects of mechanical forces on osteogenesis and osteoclastogenesis in human periodontal ligament fibroblasts: A systematic review of in vitro studies. Bone Joint Res. 2019;8:19–31. doi:10.1302/2046-3758.81.BJR-2018-0060.R1.

145. Lima VP, Soares K, Caldeira VS, Faria-E-Silva AL, Loomans B, Moraes RR. Airborne-particle Abrasion and Dentin Bonding: Systematic Review and Meta-analysis. Operative dentistry. 2021;46:E21-E33. doi:10.2341/19-216-L.
146. Lin Z, Ling L-QR, Ng M, Matlub L, Mehta K, Linus RA, et al. The effect of anticoagulants on oral squamous cell carcinoma: A systematic review. J Oral Pathol Med. 2021;50:118–21. doi:10.1111/jop.13125.
147. Mai HY, Lee WK, Kwon T-G, Lee D-H. Reliability of digital measurement methods on the marginal fit of fixed prostheses: A systematic review and meta-analysis of in vitro studies. J Prosthet Dent. 2020;124:350.e1-350.e11. doi:10.1016/j.prosdent.2020.04.011.
148. Mendes LT, Loomans BAC, Opdam NJM, Da Silva CL, Casagrande L, Lenzi TL. Silane Coupling Agents are Beneficial for Resin Composite Repair: A Systematic Review and Meta-Analysis of In Vitro Studies. J Adhes Dent. 2020;22:443–53. doi:10.3290/j.jad.a45175.
149. Mishra L, Khan AS, Velo MMdAC, Panda S, Zavattini A, Rizzante FAP, et al. Effects of Surface Treatments of Glass Fiber-Reinforced Post on Bond Strength to Root Dentine: A Systematic Review. Materials (Basel) 2020. doi:10.3390/ma13081967.
150. Mohamed RN, Basha S, Al-Thomali Y, Saleh Alshamrani A, Salem Alzahrani F, Tawfik Enan E. Self-assembling peptide P11-4 in remineralization of enamel caries - a systematic review of in-vitro studies. Acta Odontol Scand. 2021;79:139–46. doi:10.1080/00016357.2020.1825799.
151. Moura DMD, Veríssimo AH, Leite Vila-Nova TE, Calderon PS, Özcan M, Assunção Souza RO. Which surface treatment promotes higher bond strength for the repair of resin nanoceramics and polymer-infiltrated ceramics? A systematic review and meta-analysis. J Prosthet Dent 2021. doi:10.1016/j.prosdent.2020.06.009.
152. Mustafa HA, Soares AP, Paris S, Elhennawy K, Zaslansky P. The forgotten merits of GIC restorations: a systematic review. Clin Oral Investig. 2020;24:2189–201. doi:10.1007/s00784-020-03334-0.
153. Nimbeni SB, Nimbeni BS, Divakar DD. Role of Chitosan in Remineralization of Enamel and Dentin: A Systematic Review. Int J Clin Pediatr Dent. 2021;14:562–8. doi:10.5005/jp-journals-10005-1971.
154. Nogueira IdO, Oliveira PFG de, Magno MB, Ferreira DMTP, Maia LC, Rabello TB. Does the application of an adhesive layer improve the bond strength of etched and silanized glass-ceramics to resin-based materials? A systematic review and meta-analysis. J Prosthet Dent. 2021;125:56–64. doi:10.1016/j.prosdent.2019.12.005.
155. Oliveira E de, Zancanaro de Figueiredo E, Spohr AM, Lima Grossi M. Properties of Acrylic Resin For CAD/CAM: A Systematic Review and Meta-Analysis of In Vitro Studies. J Prosthodont. 2021;30:656–64. doi:10.1111/jopr.13394.
156. Oliveira Limírio JPJ de, Gomes JMdL, Alves Rezende MCR, Lemos CAA, Del Rosa CDRD, Pellizzer EP. Mechanical properties of polymethyl methacrylate as a denture base: Conventional versus CAD-CAM resin - A systematic review and meta-analysis of in vitro studies. J Prosthet Dent 2021. doi:10.1016/j.prosdent.2021.03.018.
157. Özcan M, Koc-Dundar B. Composite–composite adhesion in dentistry: a systematic review and meta-analysis. Journal of Adhesion Science and Technology. 2014;28:2209–29. doi:10.1080/01694243.2014.954659.
158. Parize H, Dias Corpa Tardelli J, Bohner L, Sesma N, Muglia VA, Cândido Dos Reis A. Digital versus conventional workflow for the fabrication of physical casts for fixed prosthodontics: A systematic review of accuracy. J Prosthet Dent 2021. doi:10.1016/j.prosdent.2020.12.008.

159. Parolia A, Nikolopoulou D, Lim BSH, Kanagasingam S. Comparison of antibacterial effectiveness between Sealapex and AH-plus sealer against Enterococcus faecalis: a systematic review of in vitro studies. Giornale Italiano di Endodonzia 2020. doi:10.32067/GIE.2020.34.02.05.
160. Paul S, Hanisch O, Nesic D. Human gingival fibroblast proliferation on materials used for dental implant abutments: a systematic review. Int J Prosthodont. 2021;34:811–28. doi:10.11607/ijp.7388.
161. Pereira GKR, Fraga S, Montagner AF, Soares FZM, Kleverlaan CJ, Valandro LF. The effect of grinding on the mechanical behavior of Y-TZP ceramics: A systematic review and meta-analyses. J Mech Behav Biomed Mater. 2016;63:417–42. doi:10.1016/j.jmbbm.2016.06.028.
162. Pires Lopes LC, Terada RSS, Tsuzuki FM, Giannini M, Hirata R. Heating and preheating of dental restorative materials-a systematic review. Clin Oral Investig. 2020;24:4225–35. doi:10.1007/s00784-020-03637-2.
163. Portela NN, Rech JP, Marchionatti AME, Barasuol JC. Techniques to address fractured instruments in the middle or apical third of the root canal in human permanent teeth: a systematic review of the in vitro studies. Clin Oral Investig. 2022;26:131–9. doi:10.1007/s00784-021-04235-6.
164. Pourhajibagher M, Sodagar A, Bahador A. An in vitro evaluation of the effects of nanoparticles on shear bond strength and antimicrobial properties of orthodontic adhesives: A systematic review and meta-analysis study. Int Orthod. 2020;18:203–13. doi:10.1016/j.ortho.2020.01.011.
165. Purger LO, Tavares SJ, Martinez RL, Caldas I, Antunes LA, Scelza MZ. Comparing Techniques for Removing Fiber Endodontic Posts: A Systematic Review. J Contemp Dent Pract. 2021;22:587–95.
166. Reis-Prado AHD, Abreu LG, Tavares WLF, Da Peixoto IFC, Viana ACD, Oliveira EMC de, et al. Comparison between immediate and delayed post space preparations: a systematic review and meta-analysis. Clin Oral Investig. 2021;25:417–40. doi:10.1007/s00784-020-03690-x.
167. Revilla-León M, Gómez-Polo M, Park SH, Barmak BA, Özcan M. Adhesion of veneering porcelain to cobalt-chromium dental alloys processed with casting, milling, and additive manufacturing methods: A systematic review and meta-analysis. J Prosthet Dent 2021. doi:10.1016/j.prosdent.2021.01.001.
168. Rodríguez-Barragué J, Vola-Gelmini J, Skuras-Siedemburg M, Rivera-Gonzaga JA, Cuevas-Suarez CE. Natural antioxidants to restore immediate bond strength to bleached enamel: Systematic review and meta-analysis of in vitro studies. Journal of esthetic and restorative dentistry : official publication of the American Academy of Esthetic Dentistry … [et al.]. 2021;33:702–12. doi:10.1111/jerd.12743.
169. Saeed M, Al-Obadi M, Salim A, Alsawaf AY, Hadi K. Impact of Access Cavity Design on Fracture Resistance of Endodontically Treated Molars: A Systematic Review. Clin Cosmet Investig Dent. 2021;13:1–10. doi:10.2147/CCIDE.S287995.
170. Sanz JL, Forner L, Almudéver A, Guerrero-Gironés J, Llena C. Viability and Stimulation of Human Stem Cells from the Apical Papilla (hSCAPs) Induced by Silicate-Based Materials for Their Potential Use in Regenerative Endodontics: A Systematic Review. Materials (Basel) 2020. doi:10.3390/ma13040974.
171. Sanz JL, Forner L, Llena C, Guerrero-Gironés J, Melo M, Rengo S, et al. Cytocompatibility and Bioactive Properties of Hydraulic Calcium Silicate-Based Cements

(HCSCs) on Stem Cells from Human Exfoliated Deciduous Teeth (SHEDs): A Systematic Review of In Vitro Studies. J Clin Med 2020. doi:10.3390/jcm9123872.
172. Sanz JL, Guerrero-Gironés J, Pecci-Lloret MP, Pecci-Lloret MR, Melo M. Biological interactions between calcium silicate-based endodontic biomaterials and periodontal ligament stem cells: A systematic review of in vitro studies. Int Endod J. 2021;54:2025–43. doi:10.1111/iej.13600.
173. Sanz JL, Rodríguez-Lozano FJ, Lopez-Gines C, Monleon D, Llena C, Forner L. Dental stem cell signaling pathway activation in response to hydraulic calcium silicate-based endodontic cements: A systematic review of in vitro studies. Dent Mater. 2021;37:e256-e268. doi:10.1016/j.dental.2021.01.025.
174. Savian TG, Oling J, Soares F, Rocha RO. Vital Bleaching Influences the Bond Strength of Adhesive Systems to Enamel and Dentin: A Systematic Review and Meta-Analysis of In Vitro Studies. Operative dentistry. 2021;46:E80-E97. doi:10.2341/20-035-LIT.
175. Silva EJNL, Cardoso ML, Rodrigues JP, De-Deus G, Da Fidalgo TKS. Solubility of bioceramic- and epoxy resin-based root canal sealers: A systematic review and meta-analysis. Australian endodontic journal : the journal of the Australian Society of Endodontology Inc. 2021;47:690–702. doi:10.1111/aej.12487.
176. Spitz A, Christovam IO, Marañón-Vásquez GA, Masterson DF, Adesse D, Maia LC, Bolognese AM. Global gene expression profile of periodontal ligament cells submitted to mechanical loading: A systematic review. Arch Oral Biol. 2020;118:104884. doi:10.1016/j.archoralbio.2020.104884.
177. Stasic JN, Pficer JK, Milicic B, Puač N, Miletic V. Effects of non-thermal atmospheric plasma on dentin wetting and adhesive bonding efficiency: Systematic review and meta-analysis. J Dent. 2021;112:103765. doi:10.1016/j.jdent.2021.103765.
178. Tan NCP, Khan A, Antunes E, Miller CM, Sharma D. The effects of physical decontamination methods on zirconia implant surfaces: a systematic review. J Periodontal Implant Sci. 2021;51:298–315. doi:10.5051/jpis.2005080254.
179. Teja KV, Ramesh S, Battineni G, Vasundhara KA, Jose J, Janani K. The effect of various in-vitro and ex-vivo parameters on irrigant flow and apical pressure using manual syringe needle irrigation: Systematic review. Saudi Dent J 2021. doi:10.1016/j.sdentj.2021.12.001.
180. Thakur J, Parlani S, Shivakumar S, Jajoo K. Accuracy of marginal fit of an implant-supported framework fabricated by 3D printing versus subtractive manufacturing technique: A systematic review and meta-analysis. J Prosthet Dent 2021. doi:10.1016/j.prosdent.2021.05.010.
181. Troconis CCM, Molina Pérez S. Bond strength of self-adhesive flowable resin composites to tooth structure. Braz. J. Oral Sci. 2021;20:e213641. doi:10.20396/bjos.v20i00.8663641.
182. Uzunoglu-Özyürek E, Küçükkaya Eren S, Karahan S. Contribution of XP-Endo files to the root canal filling removal: A systematic review and meta-analysis of in vitro studies. Australian endodontic journal : the journal of the Australian Society of Endodontology Inc. 2021;47:703–14. doi:10.1111/aej.12503.
183. Wang C, Shi Y-F, Xie P-J, Wu J-H. Accuracy of digital complete dentures: A systematic review of in vitro studies. J Prosthet Dent. 2021;125:249–56. doi:10.1016/j.prosdent.2020.01.004.
184. Wehner C, Lettner S, Moritz A, Andrukhov O, Rausch-Fan X. Effect of bisphosphonate treatment of titanium surfaces on alkaline phosphatase activity in osteoblasts:

a systematic review and meta-analysis. BMC Oral Health. 2020;20:125. doi:10.1186/s12903-020-01089-4.
185. Willis J, Li S, Crean SJ, Barrak FN. Is titanium alloy Ti-6Al-4 V cytotoxic to gingival fibroblasts-A systematic review. Clin Exp Dent Res. 2021;7:1037–44. doi:10.1002/cre2.444.

**Additional file 5**. List of excluded articles after assessment of titles and abstracts

1. Abduo J, Judge RB. Implications of implant framework misfit: a systematic review of biomechanical sequelae. Int J Oral Maxillofac Implants. 2014;29:608–21. doi:10.11607/jomi.3418.

Reason: SR including different types of subjects

2. Abduo J, Lyons K, Swain M. Fit of zirconia fixed partial denture: a systematic review. J Oral Rehabil. 2010;37:866–76. doi:10.1111/j.1365-2842.2010.02113.x.

Reason: SR including different types of subjects

3. Ahmed T, Rahman NA, Alam MK. Assessment of in vivo bond strength studies of the orthodontic bracket-adhesive system: A systematic review. Eur J Dent. 2018;12:602–9. doi:10.4103/ejd.ejd_22_18.

Reason: SR including different types of subjects

4. Akram Z, Rahim ZHA, Taiyeb-Ali TB, Shahdan MSA, Baharuddin NA, Vaithilingam RD, Safii SH. Resistin as potential biomarker for chronic periodontitis: A systematic review and meta-analysis. Arch Oral Biol. 2017;73:311–20. doi:10.1016/j.archoralbio.2016.08.016.

Reason: primary in vitro studies were excluded

5. Al-Amleh B, Lyons K, Swain M. Clinical trials in zirconia: a systematic review. J Oral Rehabil. 2010;37:641–52. doi:10.1111/j.1365-2842.2010.02094.x.

Reason: SR including different types of subjects

6. Alasiri RA, Algarni HA, Alasiri RA. Ocular hazards of curing light units used in dental practice - A systematic review. Saudi Dent J. 2019;31:173–80. doi:10.1016/j.sdentj.2019.02.031.

Reason: SR including different types of subjects

7. Alikhasi M, Alsharbaty MHM, Moharrami M. Digital Implant Impression Technique Accuracy: A Systematic Review. Implant dentistry. 2017;26:929–35. doi:10.1097/ID.0000000000000683.

Reason: SR including different types of subjects

8. Almehmadi AH, Alghamdi F. Biomarkers of alveolar bone resorption in gingival crevicular fluid: A systematic review. Arch Oral Biol. 2018;93:12–21. doi:10.1016/j.archoralbio.2018.05.004.

Reason: SR including different types of subjects

9. Altaii M, Richards L, Rossi-Fedele G. Histological assessment of regenerative endodontic treatment in animal studies with different scaffolds: A systematic review. Dent Traumatol. 2017;33:235–44. doi:10.1111/edt.12338.

Reason: SR including different types of subjects

10. Alzahrani AA. Association between human herpes virus and aggressive periodontitis: A systematic review. The Saudi Journal for Dental Research. 2017;8:97–104. doi:10.1016/j.sjdr.2016.06.004.

Reason: primary in vitro studies were excluded

11. Ammari MM, Soviero VM, da Silva Fidalgo TK, Lenzi M, Ferreira DMTP, Mattos CT, et al. Is non-cavitated proximal lesion sealing an effective method for caries control in primary and permanent teeth? A systematic review and meta-analysis. J Dent. 2014;42:1217–27. doi:10.1016/j.jdent.2014.07.015.

Reason: SR including different types of subjects

12. Ancira-González L, Esparza-Villalpando V, Garrocho-Rangel A, Pozos-Guillén A. White Spot Lesion Remineralisation Agents in Primary Teeth: A Systematic Review. Oral Health Prev Dent. 2018;16:391–400. doi:10.3290/j.ohpd.a41404.

Reason: SR including different types of subjects

13. Anusavice KJ, Kakar K, Ferree N. Which mechanical and physical testing methods are relevant for predicting the clinical performance of ceramic-based dental prostheses? Clin Oral Implants Res. 2007;18 Suppl 3:218–31. doi:10.1111/j.1600-0501.2007.01460.x.

Reason: SR including different types of subjects

14. Aragón MLC, Pontes LF, Bichara LM, Flores-Mir C, Normando D. Validity and reliability of intraoral scanners compared to conventional gypsum models measurements: a systematic review. Eur J Orthod. 2016;38:429–34. doi:10.1093/ejo/cjw033.

Reason: SR including different types of subjects

15. Avila-Ortiz G, Bartold PM, Giannobile W, Katagiri W, Nares S, Rios H, et al. Biologics and Cell Therapy Tissue Engineering Approaches for the Management of the Edentulous Maxilla: A Systematic Review. Int J Oral Maxillofac Implants. 2016;31 Suppl:s121-64. doi:10.11607/jomi.16suppl.g4.

Reason: SR including different types of subjects

16. Azarpazhooh A, Limeback H. Clinical efficacy of casein derivatives: a systematic review of the literature. Journal of the American Dental Association (1939). 2008;139:915-24; quiz 994-5. doi:10.14219/jada.archive.2008.0278.

Reason: SR including different types of subjects

17. Azarpazhooh A, Limeback H. The application of ozone in dentistry: a systematic review of literature. J Dent. 2008;36:104–16. doi:10.1016/j.jdent.2007.11.008.

Reason: SR including different types of subjects

18. Azarpazhooh A, Main PA. Efficacy of dental prophylaxis (rubber cup) for the prevention of caries and gingivitis: a systematic review of literature. Br Dent J. 2009;207:E14; discussion 328-9. doi:10.1038/sj.bdj.2009.899.

Reason: SR including different types of subjects

19. Azarpazhooh A, Main PA. Summary of: Efficacy of dental prophylaxis (rubber cup) for the prevention of caries and gingivitis: a systematic review of literature. Br Dent J. 2009;207:328–9. doi:10.1038/sj.bdj.2009.878.

Reason: Other types of study design

20. Azuma MM, Samuel RO, Gomes-Filho JE, Dezan-Junior E, Cintra LTA. The role of IL-6 on apical periodontitis: a systematic review. Int Endod J. 2014;47:615–21. doi:10.1111/iej.12196.

Reason: SR including different types of subjects

21. Baig MR. Accuracy of impressions of multiple implants in the edentulous arch: a systematic review. Int J Oral Maxillofac Implants. 2014;29:869–80. doi:10.11607/jomi.3233.

Reason: SR including different types of subjects

22. Benetti F, Lemos CAA, Oliveira Gallinari M de, Terayama AM, Briso ALF, Castilho Jacinto R de, et al. Influence of different types of light on the response of the pulp tissue in dental bleaching: a systematic review. Clin Oral Investig. 2018;22:1825–37. doi:10.1007/s00784-017-2278-9.

Reason: SR including different types of subjects

23. Bertl K, Parllaku A, Pandis N, Buhlin K, Klinge B, Stavropoulos A. The effect of local and systemic statin use as an adjunct to non-surgical and surgical periodontal therapy-A systematic review and meta-analysis. J Dent. 2017;67:18–28. doi:10.1016/j.jdent.2017.08.011.

Reason: SR including different types of subjects

24. Bertolini MM, Del Bel Cury AA, Pizzoloto L, Acapa IRH, Shibli JA, Bordin D. Does traumatic occlusal forces lead to peri-implant bone loss? A systematic review. Braz Oral Res. 2019;33:e069. doi:10.1590/1807-3107bor-2019.vol33.0069.

Reason: SR including different types of subjects

25. Bessa-Nogueira RV, Vasconcelos BCE, Niederman R. The methodological quality of systematic reviews comparing temporomandibular joint disorder surgical and non-surgical treatment. BMC Oral Health. 2008;8:27. doi:10.1186/1472-6831-8-27.

Reason: primary in vitro studies were excluded

26. Betancourt P, Cantin M, Fuentes R. In vitro and in vivo frecuency of MB2 canal in maxillary first molars: a systematic review. Avances en odontoestomatologia. 2014;30:11–22.

Reason: SR including different types of subjects

27. Betti BF, Everts V, Ket JCF, Tabeian H, Bakker AD, Langenbach GE, Lobbezoo F. Effect of mechanical loading on the metabolic activity of cells in the temporomandibular joint: a systematic review. Clin Oral Investig. 2018;22:57–67. doi:10.1007/s00784-017-2189-9.

Reason: SR including different types of subjects

28. Bhaskar V, Chan H-L, MacEachern M, Kripfgans OD. Updates on ultrasound research in implant dentistry: a systematic review of potential clinical indications. Dentomaxillofac Radiol. 2018;47:20180076. doi:10.1259/dmfr.20180076.

Reason: SR including different types of subjects

29. Bishti S, Strub JR, Att W. Effect of the implant-abutment interface on peri-implant tissues: a systematic review. Acta Odontol Scand. 2014;72:13–25. doi:10.3109/00016357.2013.799712.

Reason: SR including different types of subjects

30. Blázquez-Hinarejos M, Ayuso-Montero R, Jané-Salas E, López-López J. Influence of surface modified dental implant abutments on connective tissue attachment: A systematic review. Arch Oral Biol. 2017;80:185–92. doi:10.1016/j.archoralbio.2017.04.020.

Reason: SR including different types of subjects

31. Bohner LOL, Neto PT, Ahmed AS, Mori M, Laganá DC, Sesma N. CEREC Chairside System to Register and Design the Occlusion in Restorative Dentistry: A Systematic Literature Review. J Esthet Restor Dent. 2016;28:208–20. doi:10.1111/jerd.12226.

Reason: SR including different types of subjects

32. Borges GÁ, Rêgo DF, Assad DX, Coletta RD, Luca Canto G de, Guerra ENS. In vivo and in vitro effects of curcumin on head and neck carcinoma: a systematic review. Journal of oral pathology & medicine : official publication of the International Association of Oral Pathologists and the American Academy of Oral Pathology. 2017;46:3–20. doi:10.1111/jop.12455.

Reason: SR including different types of subjects

33. Boruziniat A, Gharaee S, Sarraf Shirazi A, Majidinia S, Vatanpour M. Evaluation of the efficacy of flowable composite as lining material on microleakage of composite resin restorations: A systematic review and meta-analysis. Quintessence Int. 2016;47:93–101. doi:10.3290/j.qi.a35260.

Reason: SR including different types of subjects

34. Bousnaki M, Chatziparaskeva M, Bakopoulou A, Pissiotis A, Koidis P. Variables affecting the fit of zirconia fixed partial dentures: A systematic review. J Prosthet Dent. 2020;123:686-692.e8. doi:10.1016/j.prosdent.2019.06.019.

Reason: SR including different types of subjects

35. Bover-Ramos F, Viña-Almunia J, Cervera-Ballester J, Peñarrocha-Diago M, García-Mira B. Accuracy of Implant Placement with Computer-Guided Surgery: A Systematic Review and Meta-Analysis Comparing Cadaver, Clinical, and In Vitro Studies. Int J Oral Maxillofac Implants. 2018;33:101–15. doi:10.11607/jomi.5556.

Reason: SR including different types of subjects

36. Brouwer F, Askar H, Paris S, Schwendicke F. Detecting Secondary Caries Lesions: A Systematic Review and Meta-analysis. J Dent Res. 2016;95:143–51. doi:10.1177/0022034515611041.

Reason: SR including different types of subjects

37. Bühler J, Amato M, Weiger R, Walter C. A systematic review on the patient perception of periodontal treatment using air polishing devices. International journal of dental hygiene. 2016;14:4–14. doi:10.1111/idh.12119.

Reason: SR including different types of subjects

38. Buijs GJ, Stegenga B, Bos RRM. Efficacy and safety of biodegradable osteofixation devices in oral and maxillofacial surgery: a systematic review. J Dent Res. 2006;85:980–9. doi:10.1177/154405910608501102.

Reason: SR including different types of subjects

39. Cairo F, Pagliaro U, Nieri M. Treatment of gingival recession with coronally advanced flap procedures: a systematic review. J Clin Periodontol. 2008;35:136–62. doi:10.1111/j.1600-051X.2008.01267.x.

Reason: SR including different types of subjects

40. Căpută PE, Retsas A, Kuijk L, Chávez de Paz LE, Boutsioukis C. Ultrasonic Irrigant Activation during Root Canal Treatment: A Systematic Review. J Endod. 2019;45:31-44.e13. doi:10.1016/j.joen.2018.09.010.

Reason: SR including different types of subjects

41. Caricasulo R, Malchiodi L, Ghensi P, Fantozzi G, Cucchi A. The influence of implant-abutment connection to peri-implant bone loss: A systematic review and meta-analysis. Clin Implant Dent Relat Res. 2018;20:653–64. doi:10.1111/cid.12620.

Reason: SR including different types of subjects

42. Carvalho PHA, Moura LB, Trento GS, Holzinger D, Gabrielli MAC, Gabrielli MFR, Pereira Filho VA. Surgically assisted rapid maxillary expansion: a systematic review of complications. Int J Oral Maxillofac Surg. 2020;49:325–32. doi:10.1016/j.ijom.2019.08.011.

Reason: SR including different types of subjects

43. Celentano A, Tran A, Testa C, Thayanantha K, Tan-Orders W, Tan S, et al. The protective effects of Kava (Piper Methysticum) constituents in cancers: A systematic review. Journal of oral pathology & medicine : official publication of the International Association of Oral Pathologists and the American Academy of Oral Pathology. 2019;48:510–29. doi:10.1111/jop.12900.

Reason: SR including different types of subjects

44. Chang E, Lam E, Shah P, Azarpazhooh A. Cone-beam Computed Tomography for Detecting Vertical Root Fractures in Endodontically Treated Teeth: A Systematic Review. J Endod. 2016;42:177–85. doi:10.1016/j.joen.2015.10.005.

Reason: SR including different types of subjects

45. Chibinski AC, Wambier LM, Feltrin J, Loguercio AD, Wambier DS, Reis A. Silver Diamine Fluoride Has Efficacy in Controlling Caries Progression in Primary Teeth: A Systematic Review and Meta-Analysis. Caries Res. 2017;51:527–41. doi:10.1159/000478668.

Reason: SR including different types of subjects

46. Cidreira Boaro LC, Pereira Lopes D, Souza ASC de, Lie Nakano E, Ayala Perez MD, Pfeifer CS, Gonçalves F. Clinical performance and chemical-physical properties of bulk fill composites resin -a systematic review and meta-analysis. Dent Mater. 2019;35:e249-e264. doi:10.1016/j.dental.2019.07.007.

Reason: SR including different types of subjects

47. Condò R, Cioffi A, Riccio A, Totino M, Condò SG, Cerroni L. Sealants in dentistry: a systematic review of the literature. Oral Implantol (Rome). 2013;6:67–74.

Reason: primary in vitro studies were excluded

48. Contrepois M, Soenen A, Bartala M, Laviole O. Marginal adaptation of ceramic crowns: a systematic review. J Prosthet Dent. 2013;110:447-454.e10. doi:10.1016/j.prosdent.2013.08.003.

Reason: SR including different types of subjects

49. Corica A, Caprioglio A. Meta-analysis of the prevalence of tooth wear in primary dentition. Eur J Paediatr Dent. 2014;15:385–8.

Reason: SR including different types of subjects

50. da Veiga AMA, Cunha AC, Ferreira DMTP, da Silva Fidalgo TK, Chianca TK, Reis KR, Maia LC. Longevity of direct and indirect resin composite restorations in permanent posterior teeth: A systematic review and meta-analysis. J Dent. 2016;54:1–12. doi:10.1016/j.jdent.2016.08.003.

Reason: SR including different types of subjects

51. Daltoé FP, Mendonça PP, Mantesso A, Deboni MCZ. Can SHED or DPSCs be used to repair/regenerate non-dental tissues? A systematic review of in vivo studies. Braz Oral Res 2014. doi:10.1590/1807-3107bor-2014.vol28.0037.

Reason: SR including different types of subjects

52. Del Fabbro M, Afrashtehfar KI, Corbella S, El-Kabbaney A, Perondi I, Taschieri S. In Vivo and In Vitro Effectiveness of Rotary Nickel-Titanium vs Manual Stainless Steel Instruments for Root Canal Therapy: Systematic Review and Meta-analysis. J Evid Based Dent Pract. 2018;18:59–69. doi:10.1016/j.jebdp.2017.08.001.

Reason: SR including different types of subjects

53. Deo SD, Shetty SK, Kulloli A, Chavan R, Dholakia P, Ligade S, Dharmarajan G. Efficacy of free gingival graft in the treatment of Miller Class I and Class II localized gingival recessions: A systematic review. J Indian Soc Periodontol. 2019;23:93–9. doi:10.4103/jisp.jisp_102_18.

Reason: SR including different types of subjects

54. Dietschi D, Duc O, Krejci I, Sadan A. Biomechanical considerations for the restoration of endodontically treated teeth: a systematic review of the literature, Part II (Evaluation of fatigue behavior, interfaces, and in vivo studies). Quintessence Int. 2008;39:117–29.

Reason: SR including different types of subjects

55. do Amaral GS, Negrini T, Maltz M, Arthur RA. Restorative materials containing antimicrobial agents: is there evidence for their antimicrobial and anticaries effects? A systematic review. Aust Dent J. 2016;61:6–15. doi:10.1111/adj.12338.

Reason: SR including different types of subjects

56. Draenert ME, Jakob M, Kunzelmann K-H, Hickel R. The prevalence of tooth hypersensitivity following periodontal therapy with special reference to root scaling. A systematic review of the literature. Am J Dent. 2013;26:21–7.

Reason: SR including different types of subjects

57. Dutra D, Pereira G, Kantorski KZ, Valandro LF, Zanatta FB. Does Finishing and Polishing of Restorative Materials Affect Bacterial Adhesion and Biofilm Formation? A Systematic Review. Oper Dent. 2018;43:E37-E52. doi:10.2341/17-073-L.

Reason: SR including different types of subjects

58. Elshiyab SH, Nawafleh N, George R. Survival and testing parameters of zirconia-based crowns under cyclic loading in an aqueous environment: A systematic review. J Investig Clin Dent 2017. doi:10.1111/jicd.12261.

Reason: SR including different types of subjects

59. Emara R, Elhennawy K, Schwendicke F. Effects of calcium silicate cements on dental pulp cells: A systematic review. J Dent. 2018;77:18–36. doi:10.1016/j.jdent.2018.08.003.

Reason: SR including different types of subjects

60. Eramo S, Natali A, Pinna R, Milia E. Dental pulp regeneration via cell homing. Int Endod J. 2018;51:405–19. doi:10.1111/iej.12868.

Reason: SR including different types of subjects

61. Fleming PS, Eliades T, Katsaros C, Pandis N. Curing lights for orthodontic bonding: a systematic review and meta-analysis. Am J Orthod Dentofacial Orthop. 2013;143:S92-103. doi:10.1016/j.ajodo.2012.07.018.

Reason: SR including different types of subjects

62. Flügge T, van der Meer WJ, Gonzalez BG, Vach K, Wismeijer D, Wang P. The accuracy of different dental impression techniques for implant-supported dental prostheses: A systematic review and meta-analysis. Clin Oral Implants Res. 2018;29 Suppl 16:374–92. doi:10.1111/clr.13273.

Reason: SR including different types of subjects

63. Fonseca J-M, Troconis C-C, Palmier N-R, Gomes-Silva W, Paglioni M-D, Araújo A-L, et al. The impact of head and neck radiotherapy on the dentine-enamel junction: a systematic review. Med Oral Patol Oral Cir Bucal. 2020;25:e96-e105. doi:10.4317/medoral.23212.

Reason: SR including different types of subjects

64. Fontanari LA, Pimentel Lopes De Oliveira GJ, Durigan Basso TL, Marcantonio Junior E, Perez Orrico SR, Cezar Sampaio JE. The influence of different implant surfaces on osseointegration in diabetes: a systematic review of the literature. Minerva Stomatol. 2014;63:127–33.

Reason: primary in vitro studies were excluded

65. Gadkari N, Bawane S, Chopra R, Bhate K, Kulkarni D. Comparative evaluation of 2-point vs 3-point fixation in the treatment of zygomaticomaxillary complex fractures - A

systematic review. Journal of cranio-maxillo-facial surgery : official publication of the European Association for Cranio-Maxillo-Facial Surgery. 2019;47:1542–50. doi:10.1016/j.jcms.2019.07.009.

Reason: SR including different types of subjects

66. Ghanem A, Abduljabbar T, Akram Z, Vohra F, Kellesarian SV, Javed F. A systematic review and meta-analysis of pre-clinical studies assessing the effect of nicotine on osseointegration. Int J Oral Maxillofac Surg. 2017;46:496–502. doi:10.1016/j.ijom.2016.12.003.

Reason: SR including different types of subjects

67. Giannobile WV, Somerman MJ. Growth and amelogenin-like factors in periodontal wound healing. A systematic review. Ann Periodontol. 2003;8:193–204. doi:10.1902/annals.2003.8.1.193.

Reason: primary in vitro studies were excluded

68. Gibson G, Jurasic MM, Wehler CJ, Jones JA. Supplemental fluoride use for moderate and high caries risk adults: a systematic review. J Public Health Dent. 2011;71:171–84.

Reason: primary in vitro studies were excluded

69. Goiato MC, Pellizzer EP, da Silva EVF, Da Bonatto LR, dos Santos DM. Is the internal connection more efficient than external connection in mechanical, biological, and esthetical point of views? A systematic review. Oral Maxillofac Surg. 2015;19:229–42. doi:10.1007/s10006-015-0494-5.

Reason: SR including different types of subjects

70. Gomez J, Tellez M, Pretty IA, Ellwood RP, Ismail AI. Non-cavitated carious lesions detection methods: a systematic review. Community Dent Oral Epidemiol. 2013;41:54–66. doi:10.1111/cdoe.12021.

Reason: SR including different types of subjects

71. Govare N, Contrepois M. Endocrowns: A systematic review. J Prosthet Dent. 2020;123:411-418.e9. doi:10.1016/j.prosdent.2019.04.009.

Reason: SR including different types of subjects

72. Gracis S, Michalakis K, Vigolo P, Vult von Steyern P, Zwahlen M, Sailer I. Internal vs. external connections for abutments/reconstructions: a systematic review. Clin Oral Implants Res. 2012;23 Suppl 6:202–16. doi:10.1111/j.1600-0501.2012.02556.x.

Reason: SR including different types of subjects

73. Graziani F, Gennai S, Cei S, Ducci F, Discepoli N, Carmignani A, Tonetti M. Does enamel matrix derivative application provide additional clinical benefits in residual periodontal pockets associated with suprabony defects? A systematic review and meta-analysis of randomized clinical trials. J Clin Periodontol. 2014;41:377–86. doi:10.1111/jcpe.12218.

Reason: SR including different types of subjects

74. Grisa A, Veitz-Keenan A. Is osteoporosis a risk factor for implant survival or failure? Evidence-based dentistry. 2018;19:51–2. doi:10.1038/sj.ebd.6401307.

Reason: SR including different types of subjects

75. Hamama HHH, Yiu CKY, Burrow MF, King NM. Systematic Review and Meta-Analysis of Randomized Clinical Trials on Chemomechanical Caries Removal. Oper Dent. 2015;40:E167-78. doi:10.2341/14-021-LIT.

Reason: SR including different types of subjects

76. Hasanzade M, Shirani M, Afrashtehfar KI, Naseri P, Alikhasi M. In Vivo and In Vitro Comparison of Internal and Marginal Fit of Digital and Conventional Impressions for Full-Coverage Fixed Restorations: A Systematic Review and Meta-analysis. J Evid Based Dent Pract. 2019;19:236–54. doi:10.1016/j.jebdp.2019.04.003.

Reason: SR including different types of subjects

77. Heydecke G, Peters MC. The restoration of endodontically treated, single-rooted teeth with cast or direct posts and cores: a systematic review. J Prosthet Dent. 2002;87:380–6. doi:10.1067/mpr.2002.123848.

Reason: SR including different types of subjects

78. Hmaidouch R, Weigl P. Tooth wear against ceramic crowns in posterior region: a systematic literature review. Int J Oral Sci. 2013;5:183–90. doi:10.1038/ijos.2013.73.

Reason: SR including different types of subjects

79. Höchli D, Hersberger-Zurfluh M, Papageorgiou SN, Eliades T. Interventions for orthodontically induced white spot lesions: a systematic review and meta-analysis. Eur J Orthod. 2017;39:122–33. doi:10.1093/ejo/cjw065.

Reason: SR including different types of subjects

80. Hosseinpour S, Ghazizadeh Ahsaie M, Rezai Rad M, Baghani MT, Motamedian SR, Khojasteh A. Application of selected scaffolds for bone tissue engineering: a systematic review. Oral Maxillofac Surg. 2017;21:109–29. doi:10.1007/s10006-017-0608-3.

Reason: SR including different types of subjects

81. Hoxha A, Gillam DG, Bushby AJ, Agha A, Patel MP. Layered Double Hydroxide Fluoride Release in Dental Applications: A Systematic Review. Dent J (Basel) 2019. doi:10.3390/dj7030087.

Reason: SR including different types of subjects

82. Isfeld D, Lagravere M, Leon-Salazar V, Flores-Mir C. Novel methodologies and technologies to assess mid-palatal suture maturation: a systematic review. Head Face Med. 2017;13:13. doi:10.1186/s13005-017-0144-2.

Reason: SR including different types of subjects

83. Jacobs R, Vranckx M, Vanderstuyft T, Quirynen M, Salmon B. CBCT vs other imaging modalities to assess peri-implant bone and diagnose complications: a systematic review. Eur J Oral Implantol. 2018;11 Suppl 1:77–92.

Reason: SR including different types of subjects

84. Jakobsen C, Sørensen JA, Kassem M, Thygesen TH. Mesenchymal stem cells in oral reconstructive surgery: a systematic review of the literature. J Oral Rehabil. 2013;40:693–706. doi:10.1111/joor.12079.

Reason: SR including different types of subjects

85. Javed F, Al Amri MD, Kellesarian SV, Al-Askar M, Al-Kheraif AA, Romanos GE. Laminin coatings on implant surfaces promote osseointegration: Fact or fiction? Arch Oral Biol. 2016;68:153–61. doi:10.1016/j.archoralbio.2016.05.005.

Reason: SR including different types of subjects

86. Jayaraman J, Nagendrababu V, Pulikkotil SJ, Veettil SK, Dhar V. Effectiveness of formocresol and ferric sulfate as pulpotomy material in primary molars: a systematic review and meta-analysis with trial sequential analysis of randomized clinical trials. Quintessence Int. 2020;51:38–48. doi:10.3290/j.qi.a43617.

Reason: SR including different types of subjects

87. Jokstad A, Ganeles J. Systematic review of clinical and patient-reported outcomes following oral rehabilitation on dental implants with a tapered compared to a non-tapered implant design. Clin Oral Implants Res. 2018;29 Suppl 16:41–54. doi:10.1111/clr.13128.

Reason: SR including different types of subjects

88. Jung RE, Schneider D, Ganeles J, Wismeijer D, Zwahlen M, Hämmerle CHF, Tahmaseb A. Computer technology applications in surgical implant dentistry: a systematic review. Int J Oral Maxillofac Implants. 2009;24 Suppl:92–109.

Reason: SR including different types of subjects

89. Katsoulis J, Takeichi T, Sol Gaviria A, Peter L, Katsoulis K. Misfit of implant prostheses and its impact on clinical outcomes. Definition, assessment and a systematic review of the literature. Eur J Oral Implantol. 2017;10 Suppl 1:121–38.

Reason: SR including different types of subjects

90. Kellesarian SV, Al Amri MD, Al-Kheraif AA, Ghanem A, Malmstrom H, Javed F. Efficacy of Local and Systemic Statin Delivery on the Osseointegration of Implants: A Systematic Review. Int J Oral Maxillofac Implants. 2017;32:497–506. doi:10.11607/jomi.4955.

Reason: SR including different types of subjects

91. Kelly JR. Developing meaningful systematic review of CAD/CAM reconstructions and fiber-reinforced composites. Clin Oral Implants Res. 2007;18 Suppl 3:205–17. doi:10.1111/j.1600-0501.2007.01443.x.

Reason: SR including different types of subjects

92. Khojasteh A, Kheiri L, Motamedian SR, Nadjmi N. Regenerative medicine in the treatment of alveolar cleft defect: A systematic review of the literature. Journal of cranio-maxillo-facial surgery : official publication of the European Association for Cranio-Maxillo-Facial Surgery. 2015;43:1608–13. doi:10.1016/j.jcms.2015.06.041.

Reason: SR including different types of subjects

93. Kloukos D, Pandis N, Eliades T. Bisphenol-A and residual monomer leaching from orthodontic adhesive resins and polycarbonate brackets: a systematic review. Am J Orthod Dentofacial Orthop. 2013;143:S104-12.e1-2. doi:10.1016/j.ajodo.2012.11.015.

Reason: SR including different types of subjects

94. Konstantinidi E, Psimma Z, Chávez de Paz LE, Boutsioukis C. Apical negative pressure irrigation versus syringe irrigation: a systematic review of cleaning and disinfection of the root canal system. Int Endod J. 2017;50:1034–54. doi:10.1111/iej.12725.

Reason: SR including different types of subjects

95. Laaksonen M, Sorsa T, Salo T. Letter to the Editor concerning "Emdogain(®) in carcinogenesis: a systematic review of in vitro studies" by Laaksonen M, Sorsa T, Salo T (2010) J Oral Sci 52, 1-11. J Oral Sci. 2010;52:665–6. doi:10.2334/josnusd.52.665.

Reason: Other types of study design

96. Lafuente Ibáñez de Mendoza I, Maritxalar Mendia X, La García de Fuente AM, Quindós Andrés G, Aguirre Urizar JM. Role of Porphyromonas gingivalis in oral squamous cell carcinoma development: A systematic review. J Periodontal Res. 2020;55:13–22. doi:10.1111/jre.12691.

Reason: SR including different types of subjects

97. Lam OLT, McGrath C, Bandara HMHN, Li LSW, Samaranayake LP. Oral health promotion interventions on oral reservoirs of staphylococcus aureus: a systematic review. Oral Dis. 2012;18:244–54. doi:10.1111/j.1601-0825.2011.01874.x.

Reason: SR including different types of subjects

98. Larsson C, Wennerberg A. The clinical success of zirconia-based crowns: a systematic review. Int J Prosthodont. 2014;27:33–43. doi:10.11607/ijp.3647.

Reason: SR including different types of subjects

99. Lemos CAA, Souza Batista VE de, Almeida DAdF, Santiago Júnior JF, Verri FR, Pellizzer EP. Evaluation of cement-retained versus screw-retained implant-supported restorations for marginal bone loss: A systematic review and meta-analysis. J Prosthet Dent. 2016;115:419–27. doi:10.1016/j.prosdent.2015.08.026.

Reason: SR including different types of subjects

100. Lemos CAA, Verri FR, Bonfante EA, Santiago Júnior JF, Pellizzer EP. Comparison of external and internal implant-abutment connections for implant supported prostheses. A systematic review and meta-analysis. J Dent. 2018;70:14–22. doi:10.1016/j.jdent.2017.12.001.

Reason: SR including different types of subjects

101. Li J, Xie X, Wang Y, Yin W, Antoun JS, Farella M, Mei L. Long-term remineralizing effect of casein phosphopeptide-amorphous calcium phosphate (CPP-ACP) on early caries lesions in vivo: a systematic review. J Dent. 2014;42:769–77. doi:10.1016/j.jdent.2014.03.015.

Reason: SR including different types of subjects

102. Lin Z, Rios HF, Cochran DL. Emerging regenerative approaches for periodontal reconstruction: a systematic review from the AAP Regeneration Workshop. J Periodontol. 2015;86:S134-52. doi:10.1902/jop.2015.130689.

Reason: SR including different types of subjects

103. Linkevicius T, Apse P. Biologic width around implants. An evidence-based review. Stomatologija. 2008;10:27–35.

Reason: SR including different types of subjects

104. Linkevicius T, Apse P. Influence of abutment material on stability of peri-implant tissues: a systematic review. Int J Oral Maxillofac Implants. 2008;23:449–56.

Reason: primary in vitro studies were excluded

105. Lozano-Carrascal N, Salomó-Coll O, Hernández-Alfaro F, Gehrke S-A, Gargallo-Albiol J, Calvo-Guirado J-L. Do topical applications of bisphosphonates improve bone formation in oral implantology? A systematic review. Med Oral Patol Oral Cir Bucal. 2017;22:e512-e519. doi:10.4317/medoral.21887.

Reason: SR including different types of subjects

106. Lu C, Liu M. Letter to "Evaluation of the efficiency of fluoride-releasing adhesives for preventing secondary caries in vitro: a systematic review and meta-analysis". Eur Arch Paediatr Dent. 2019;20:623. doi:10.1007/s40368-019-00460-6.

Reason: Other types of study design

107. Luo JD, Miller C, Jirjis T, Nasir M, Sharma D. The effect of non-steroidal anti-inflammatory drugs on the osteogenic activity in osseointegration: a systematic review. Int J Implant Dent. 2018;4:30. doi:10.1186/s40729-018-0141-7.

Reason: SR including different types of subjects

108. Lynch E. Comment on "The application of ozone in dentistry: A systematic review of the literature". J Dent. 2009;37:406-10; author reply 411-2. doi:10.1016/j.jdent.2008.11.002.

Reason: Other types of study design

109. Ma X, Lin X, Zhong T, Xie F. Evaluation of the efficacy of casein phosphopeptide-amorphous calcium phosphate on remineralization of white spot lesions in vitro and clinical research: a systematic review and meta-analysis. BMC Oral Health. 2019;19:295. doi:10.1186/s12903-019-0977-0.

Reason: SR including different types of subjects

110. Madrid Troconis CC, Santos-Silva AR, Brandão TB, Lopes MA, Goes MF de. Impact of head and neck radiotherapy on the mechanical behavior of composite resins and adhesive systems: A systematic review. Dent Mater. 2017;33:1229–43. doi:10.1016/j.dental.2017.07.014.

Reason: SR including different types of subjects

111. Makrygiannakis MA, Kaklamanos EG, Athanasiou AE. Effects of systemic medication on root resorption associated with orthodontic tooth movement: a systematic review of animal studies. Eur J Orthod. 2019;41:346–59. doi:10.1093/ejo/cjy048.

Reason: SR including different types of subjects

112. Marceliano-Alves MFV, Lima JT, Alves FRF. Final irrigation protocols in Endodontics: Systematic review. Dental Press Endod. 2018;8:24–33. doi:10.14436/2358-2545.8.3.024-033.oar.

Reason: SR including different types of subjects

113. Maroulakos M, Kamperos G, Tayebi L, Halazonetis D, Ren Y. Applications of 3D printing on craniofacial bone repair: A systematic review. J Dent. 2019;80:1–14. doi:10.1016/j.jdent.2018.11.004.

Reason: SR including different types of subjects

114. Martins FV, Vasques WF, Fonseca EM. The Letter to the Editor regarding the paper 'Evaluation of the efficiency of fluoride-releasing adhesives for preventing secondary caries in-vitro: a systematic review and meta-analysis'. Eur Arch Paediatr Dent. 2019;20:625. doi:10.1007/s40368-019-00461-5.

Reason: SR including different types of subjects

115. Marzouk T, Sathyanarayana S, Kim AS, Seminario AL, McKinney CM. A Systematic Review of Exposure to Bisphenol A from Dental Treatment. JDR Clin Trans Res. 2019;4:106–15. doi:10.1177/2380084418816079.

Reason: SR including different types of subjects

116. Meza-Mauricio J, Soto-Peñaloza D, Peñarrocha-Oltra D, Montiel-Company JM, Peruzzo DC. Locally applied statins as adjuvants to non-surgical periodontal treatment for chronic periodontitis: a systematic review and meta-analysis. Clin Oral Investig. 2018;22:2413–30. doi:10.1007/s00784-018-2507-x.

Reason: SR including different types of subjects

117. Mickenautsch S, Yengopal V, Banerjee A. Retention of orthodontic brackets bonded with resin-modified GIC versus composite resin adhesives--a quantitative systematic review of clinical trials. Clin Oral Investig. 2012;16:1–14. doi:10.1007/s00784-011-0626-8.

Reason: SR including different types of subjects

118. Miron RJ, Guillemette V, Zhang Y, Chandad F, Sculean A. Enamel matrix derivative in combination with bone grafts: A review of the literature. Quintessence Int. 2014;45:475–87. doi:10.3290/j.qi.a31541.

Reason: SR including different types of subjects

119. Monsarrat P, Garnier S, Vergnes J-N, Nasr K, Grosgogeat B, Joniot S. Survival of directly placed ormocer-based restorative materials: A systematic review and meta-analysis of clinical trials. Dent Mater. 2017;33:e212-e220. doi:10.1016/j.dental.2017.01.019.

Reason: SR including different types of subjects

120. Morillo CMR, Sloniak MC, Gonçalves F, Villar CC. Efficacy of stem cells on bone consolidation of distraction osteogenesis in animal models: a systematic review. Braz Oral Res. 2018;32:e83. doi:10.1590/1807-3107bor-2018.vol32.0083.

Reason: SR including different types of subjects

121. Najeeb S, Bds ZK, Bds SZ, Bds MSZ. Bioactivity and Osseointegration of PEEK Are Inferior to Those of Titanium: A Systematic Review. The Journal of oral implantology. 2016;42:512–6. doi:10.1563/aaid-joi-D-16-00072.

Reason: SR including different types of subjects

122. Najeeb S, Siddiqui F, Khurshid Z, Zohaib S, Zafar MS, Ansari SA. Effect of bisphosphonates on root resorption after tooth replantation - a systematic review. Dent Traumatol. 2017;33:77–83. doi:10.1111/edt.12316.

Reason: SR including different types of subjects

123. Nakamura K, Kanno T, Milleding P, Ortengren U. Zirconia as a dental implant abutment material: a systematic review. Int J Prosthodont. 2010;23:299–309.

Reason: SR including different types of subjects

124. Nienkemper M, Handschel J, Drescher D. Systematic review of mini-implant displacement under orthodontic loading. Int J Oral Sci. 2014;6:1–6. doi:10.1038/ijos.2013.92.

Reason: SR including different types of subjects

125. Norlund A, Axelsson S, Dahlen G, Espelid I, Mejare I, Tranaeus S, Twetman S. Economic aspects of the detection of occlusal dentine caries. Acta Odontol Scand. 2009;67:38–43. doi:10.1080/00016350802549106.

Reason: SR including different types of subjects

126. Omar E. Current concepts and future of noninvasive procedures for diagnosing oral squamous cell carcinoma--a systematic review. Head Face Med. 2015;11:6. doi:10.1186/s13005-015-0063-z.

Reason: SR including different types of subjects

127. Palacios-Garzón N, Mauri-Obradors E, Roselló-LLabrés X, Estrugo-Devesa A, Jané-Salas E, López-López J. Comparison of Marginal Bone Loss Between Implants with Internal

and External Connections: A Systematic Review. Int J Oral Maxillofac Implants. 2018;33:580–9. doi:10.11607/jomi.6190.

Reason: SR including different types of subjects

128. Palmer RM, Cortellini P. Periodontal tissue engineering and regeneration: Consensus Report of the Sixth European Workshop on Periodontology. J Clin Periodontol. 2008;35:83–6. doi:10.1111/j.1600-051X.2008.01262.x.

Reason: SR including different types of subjects

129. Papadiochou S, Polyzois G. Hygiene practices in removable prosthodontics: A systematic review. International journal of dental hygiene. 2018;16:179–201. doi:10.1111/idh.12323.

Reason: SR including different types of subjects

130. Papaspyridakos P, Chen C-J, Gallucci GO, Doukoudakis A, Weber H-P, Chronopoulos V. Accuracy of implant impressions for partially and completely edentulous patients: a systematic review. Int J Oral Maxillofac Implants. 2014;29:836–45. doi:10.11607/jomi.3625.

Reason: SR including different types of subjects

131. Pattanaik B, Pawar S, Pattanaik S. Biocompatible implant surface treatments. Indian J Dent Res. 2012;23:398–406. doi:10.4103/0970-9290.102240.

Reason: SR including different types of subjects

132. Pelekos G, Acharya A, Tonetti MS, Bornstein MM. Diagnostic performance of cone beam computed tomography in assessing peri-implant bone loss: A systematic review. Clin Oral Implants Res. 2018;29:443–64. doi:10.1111/clr.13143.

Reason: SR including different types of subjects

133. Pithon MM, Baião FS, Sant'Anna LID, Tanaka OM, Cople-Maia L. Effectiveness of casein phosphopeptide-amorphous calcium phosphate-containing products in the prevention and treatment of white spot lesions in orthodontic patients: A systematic review. J Investig Clin Dent. 2019;10:e12391. doi:10.1111/jicd.12391.

Reason: SR including different types of subjects

134. Pjetursson BE, Zarauz C, Strasding M, Sailer I, Zwahlen M, Zembic A. A systematic review of the influence of the implant-abutment connection on the clinical outcomes of ceramic and metal implant abutments supporting fixed implant reconstructions. Clin Oral Implants Res. 2018;29 Suppl 18:160–83. doi:10.1111/clr.13362.

Reason: SR including different types of subjects

135. Poorni S, Srinivasan MR, Nivedhitha MS. Probiotic Streptococcus strains in caries prevention: A systematic review. J Conserv Dent. 2019;22:123–8. doi:10.4103/JCD.JCD_505_18.

Reason: SR including different types of subjects

136. Putchala MC, Ramani P, Sherlin HJ, Premkumar P, Natesan A. Ascorbic acid and its pro-oxidant activity as a therapy for tumours of oral cavity -- a systematic review. Arch Oral Biol. 2013;58:563–74. doi:10.1016/j.archoralbio.2013.01.016.

Reason: SR including different types of subjects

137. Raghis TR, Mahmoud G, Hamadah O. Effectiveness of laser irradiation in preventing enamel demineralization during orthodontic treatment: A systematic review. Dent Med Probl. 2018;55:321–32. doi:10.17219/dmp/92636.

Reason: SR including different types of subjects

138. Rêgo DF, Pavan LMC, Elias ST, Luca Canto G de, Guerra ENS. Effects of metformin on head and neck cancer: a systematic review. Oral oncology. 2015;51:416–22. doi:10.1016/j.oraloncology.2015.01.007.

Reason: SR including different types of subjects

139. Romanos GE, Gupta B, Eckert SE. Distal cantilevers and implant dentistry. Int J Oral Maxillofac Implants. 2012;27:1131–6.

Reason: SR including different types of subjects

140. Rosenblatt A, Stamford TCM, Niederman R. Silver diamine fluoride: a caries "silver-fluoride bullet". J Dent Res. 2009;88:116–25. doi:10.1177/0022034508329406.

Reason: SR including different types of subjects

141. Rosso CB, Pereira KFS, Boretti VH, Arashiro FN, Guerisoli DMZ, Yoshinari GH. Postoperative pain in infected teeth after single - versus multiple-visit endodontic treatment - a systematic review. Brazilian Research in Pediatric Dentistry and Integrated Clinic. 2012;12:143–8.

Reason: SR including different types of subjects

142. Rutkūnas V, Gečiauskaitė A, Jegelevičius D, Vaitiekūnas M. Accuracy of digital implant impressions with intraoral scanners. A systematic review. Eur J Oral Implantol. 2017;10 Suppl 1:101–20.

Reason: SR including different types of subjects

143. SAATCHI M, SHOKRANEH A, NAVAEI H, MARACY MR, SHOJAEI H. Antibacterial effect of calcium hydroxide combined with chlorhexidine on Enterococcus faecalis: a systematic review and meta-analysis. J Appl Oral Sci. 2014;22:356–65. doi:10.1590/1678-775720140032.

Reason: SR including different types of subjects

144. Saha A, Shah S, Waknis P, Bhujbal P, Aher S, Vaswani V. Comparison of minimally invasive versus conventional open harvesting technique for iliac bone graft in secondary alveolar bone grafting in cleft palate patients: a systematic review. J Korean Assoc Oral Maxillofac Surg. 2019;45:241–53. doi:10.5125/jkaoms.2019.45.5.241.

Reason: SR including different types of subjects

145. Scaminaci Russo D, Cinelli F, Sarti C, Giachetti L. Adhesion to Zirconia: A Systematic Review of Current Conditioning Methods and Bonding Materials. Dent J (Basel) 2019. doi:10.3390/dj7030074.

Reason: SR including different types of subjects

146. Schwendicke F, Tzschoppe M, Paris S. Radiographic caries detection: A systematic review and meta-analysis. J Dent. 2015;43:924–33. doi:10.1016/j.jdent.2015.02.009.

Reason: SR including different types of subjects

147. Schwindling FS, Rammelsberg P, Stober T. Effect of chemical disinfection on the surface roughness of hard denture base materials: a systematic literature review. Int J Prosthodont. 2014;27:215–25. doi:10.11607/ijp.3759.

Reason: SR including different types of subjects

148. Sedrez-Porto JA, Da Rosa WLdO, da Silva AF, Münchow EA, Pereira-Cenci T. Endocrown restorations: A systematic review and meta-analysis. J Dent. 2016;52:8–14. doi:10.1016/j.jdent.2016.07.005.

Reason: SR including different types of subjects

149. Sendyk DI, Deboni MCZ, Pannuti CM, Naclério-Homem MG, Wennerberg A. The influence of statins on osseointegration: a systematic review of animal model studies. J Oral Rehabil. 2016;43:873–82. doi:10.1111/joor.12438.

Reason: SR including different types of subjects

150. Skupien JA, Valentini F, Boscato N, Pereira-Cenci T. Prevention and treatment of Candida colonization on denture liners: a systematic review. J Prosthet Dent. 2013;110:356–62. doi:10.1016/j.prosdent.2013.07.003.

Reason: SR including different types of subjects

151. Slot DE, Koster TJG, Paraskevas S, van der Weijden GA. The effect of the Vector scaler system on human teeth: a systematic review. International journal of dental hygiene. 2008;6:154–65. doi:10.1111/j.1601-5037.2008.00319.x.

Reason: SR including different types of subjects

152. Stocchero M, Toia M, Cecchinato D, Becktor JP, Coelho PG, Jimbo R. Biomechanical, Biologic, and Clinical Outcomes of Undersized Implant Surgical Preparation: A Systematic Review. Int J Oral Maxillofac Implants. 2016;31:1247–63. doi:10.11607/jomi.5340.

Reason: SR including different types of subjects

153. Susila A, Minu J. Activated Irrigation vs. Conventional non-activated Irrigation in Endodontics - A Systematic Review. Eur Endod J. 2019;4:96–110. doi:10.14744/eej.2019.80774.

Reason: SR including different types of subjects

154. Taha AA, Patel MP, Hill RG, Fleming PS. The effect of bioactive glasses on enamel remineralization: A systematic review. J Dent. 2017;67:9–17. doi:10.1016/j.jdent.2017.09.007.

Reason: SR including different types of subjects

155. Tee BC, Sun Z. Mandibular distraction osteogenesis assisted by cell-based tissue engineering: a systematic review. Orthod Craniofac Res. 2015;18 Suppl 1:39–49. doi:10.1111/ocr.12087.

Reason: SR including different types of subjects

156. Teughels W, Loozen G, Quirynen M. Do probiotics offer opportunities to manipulate the periodontal oral microbiota? J Clin Periodontol. 2011;38 Suppl 11:159–77. doi:10.1111/j.1600-051X.2010.01665.x.

Reason: SR including different types of subjects

157. The Editor recommends this issue's article to the reader: Adhesive systems for restoring primary teeth: a systematic review and meta-analysis of in vitro studies. Int J Paediatr Dent. 2016;26:321. doi:10.1111/ipd.12256.

Reason: Other types of study design

158. Tsirogiannis P, Reissmann DR, Heydecke G. Evaluation of the marginal fit of single-unit, complete-coverage ceramic restorations fabricated after digital and conventional impressions: A systematic review and meta-analysis. J Prosthet Dent. 2016;116:328-335.e2. doi:10.1016/j.prosdent.2016.01.028.

Reason: SR including different types of subjects

159. Tuin AJ, Domerchie PN, Schepers RH, Willemsen JCN, Dijkstra PU, Spijkervet FKL, et al. What is the current optimal fat grafting processing technique? A systematic review. Journal of cranio-maxillo-facial surgery : official publication of the European Association for Cranio-Maxillo-Facial Surgery. 2016;44:45–55. doi:10.1016/j.jcms.2015.10.021.

Reason: SR including different types of subjects

160. Tunkel J, Heinecke A, Flemmig TF. A systematic review of efficacy of machine-driven and manual subgingival debridement in the treatment of chronic periodontitis. J Clin Periodontol. 2002;29 Suppl 3:72-81; discussion 90-1. doi:10.1034/j.1600-051x.29.s3.4.x.

Reason: SR including different types of subjects

161. Tzanakakis E-GC, Tzoutzas IG, Koidis PT. Is there a potential for durable adhesion to zirconia restorations? A systematic review. J Prosthet Dent. 2016;115:9–19. doi:10.1016/j.prosdent.2015.09.008.

Reason: SR including different types of subjects

162. van der Weijden FA, Campbell SL, Dörfer CE, González-Cabezas C, Slot DE. Safety of oscillating-rotating powered brushes compared to manual toothbrushes: a systematic review. J Periodontol. 2011;82:5–24. doi:10.1902/jop.2010.100393.

Reason: SR including different types of subjects

163. van Hout WMMT, van der Mink Molen AB, Breugem CC, Koole R, van Cann EM. Reconstruction of the alveolar cleft: can growth factor-aided tissue engineering replace autologous bone grafting? A literature review and systematic review of results obtained with bone morphogenetic protein-2. Clin Oral Investig. 2011;15:297–303. doi:10.1007/s00784-011-0547-6.

Reason: SR including different types of subjects

164. Vansant L, Cadenas De Llano-Pérula M, Verdonck A, Willems G. Expression of biological mediators during orthodontic tooth movement: A systematic review. Arch Oral Biol. 2018;95:170–86. doi:10.1016/j.archoralbio.2018.08.003.

Reason: SR including different types of subjects

165. Varshney S, Dwivedi A, Pandey V. Antimicrobial effects of various platelet rich concentrates-vibes from in-vitro studies-a systematic review. J Oral Biol Craniofac Res. 2019;9:299–305. doi:10.1016/j.jobcr.2019.06.013.

Reason: SR including different types of subjects

166. Veitz-Keenan A, Keenan JR. To cord or not to cord? That is still a question. Evidence-based dentistry. 2017;18:21–2. doi:10.1038/sj.ebd.6401222.

Reason: SR including different types of subjects

167. Verweij JP, Jongkees FA, Anssari Moin D, Wismeijer D, van Merkesteyn JPR. Autotransplantation of teeth using computer-aided rapid prototyping of a three-dimensional replica of the donor tooth: a systematic literature review. Int J Oral Maxillofac Surg. 2017;46:1466–74. doi:10.1016/j.ijom.2017.04.008.

Reason: SR including different types of subjects

168. Wenz HJ, Bartsch J, Wolfart S, Kern M. Osseointegration and clinical success of zirconia dental implants: a systematic review. Int J Prosthodont. 2008;21:27–36.

Reason: SR including different types of subjects

169. Wenzel A, Møystad A. Work flow with digital intraoral radiography: a systematic review. Acta Odontol Scand. 2010;68:106–14. doi:10.3109/00016350903514426.

Reason: SR including different types of subjects

170. Wulfman C, Naveau A, Rignon-Bret C. Digital scanning for complete-arch implant-supported restorations: A systematic review. J Prosthet Dent. 2020;124:161–7. doi:10.1016/j.prosdent.2019.06.014.

Reason: SR including different types of subjects

171. Yaqoob A, Al Shehrani I, Alfarsi M, Baba S, Kanji MA, Hussain MW. PANORAMA OF IMPRESSION TECHNIQUES IN FIXED PARTIAL DENTURES. A SYSTEMATIC REVIEW. International Journal of MEDICAL DENTISTRY. 2018;22:70–84.

Reason: SR including different types of subjects

172. Zandoná AF, Zero DT. Diagnostic tools for early caries detection. Journal of the American Dental Association (1939). 2006;137:1675-84; quiz 1730. doi:10.14219/jada.archive.2006.0113.

Reason: SR including different types of subjects

173. Al-Dabbagh RA. Survival and success of endocrowns: A systematic review and meta-analysis. J Prosthet Dent. 2021;125:415.e1-415.e9. doi:10.1016/j.prosdent.2020.01.011.

Reason: SR including different types of subjects

174. Alenezi A, Chrcanovic B. Effects of the local administration of antibiotics on bone formation on implant surface in animal models: A systematic review and meta-analysis. Jpn Dent Sci Rev. 2020;56:177–83. doi:10.1016/j.jdsr.2020.09.003.

Reason: SR including different types of subjects

175. Alfayez AA, Alduhaymi AA, Almulhim EA, Assiri MY, Ansari SH. Effect of various antibacterial materials in dental composites: a systematic review.

Reason: SR including different types of subjects

176. Alzahrani KM. Implant Bio-mechanics for Successful Implant Therapy: A Systematic Review. J Int Soc Prev Community Dent. 2020;10:700–14. doi:10.4103/jispcd.JISPCD_138_20.

Reason: SR including different types of subjects

177. Andhare P, Datana S, Agarwal SS, Chopra SS. Comparison of in vivo and in vitro force decay of elastomeric chains/modules: a systematic review and meta analysis. J World Fed Orthod. 2021;10:155–62. doi:10.1016/j.ejwf.2021.07.003.

Reason: SR including different types of subjects

178. Assiri H, Dawasaz AA, Alahmari A, Asiri Z. Cone beam computed tomography (CBCT) in periodontal diseases: a Systematic review based on the efficacy model. BMC Oral Health. 2020;20:191. doi:10.1186/s12903-020-01106-6.

Reason: SR including different types of subjects

179. Attavar SH, Hegde MN. Effect of Irrigants and Irrigating Devices on Disinfection of Root Canal System: A Systematic Review. Journal of Advanced Oral Research. 2021;12:181–6. doi:10.1177/23202068211015878.

Reason: SR including different types of subjects

180. Behera, K., Nasim, I. Effect of nanoparticles based root canal disinfectants on enterococcus faecalis-a systematic review. International Journal of Dentistry and Oral Science. 2021:pp. 2898-2904.

Reason: SR including different types of subjects

181. Bernabeu-Mira JC, Soto-Peñaloza D, Peñarrocha-Diago M, Camacho-Alonso F, Rivas-Ballester R, Peñarrocha-Oltra D. Low-speed drilling without irrigation versus conventional drilling for dental implant osteotomy preparation: a systematic review. Clin Oral Investig. 2021;25:4251–67. doi:10.1007/s00784-021-03939-z.

Reason: SR including different types of subjects

182. Bousnaki M, Chatziparaskeva M, Bakopoulou A, Pissiotis A, Koidis P. Variables affecting the fit of zirconia fixed partial dentures: A systematic review. J Prosthet Dent. 2020;123:686-692.e8. doi:10.1016/j.prosdent.2019.06.019.

Reason: SR including different types of subjects

183. Burgueño-Barris G, Camps-Font O, Figueiredo R, Valmaseda-Castellón E. The Influence of Implantoplasty on Surface Roughness, Biofilm Formation, and Biocompatibility of Titanium Implants: A Systematic Review. Int J Oral Maxillofac Implants. 2021;36:e111–e119. doi:10.11607/jomi.8785.

Reason: SR including different types of subjects

184. Carneiro Pereira AL, Bezerra de Medeiros AK, Sousa Santos K de, Oliveira de Almeida É, Seabra Barbosa GA, da Fonte Porto Carreiro A. Accuracy of CAD-CAM systems for removable partial denture framework fabrication: A systematic review. J Prosthet Dent. 2021;125:241–8. doi:10.1016/j.prosdent.2020.01.003.

Reason: SR including different types of subjects

185. Carneiro Pereira AL, Souza Curinga MR, Melo Segundo HV, da Fonte Porto Carreiro A. Factors that influence the accuracy of intraoral scanning of total edentulous arches rehabilitated with multiple implants: A systematic review. J Prosthet Dent 2021. doi:10.1016/j.prosdent.2021.09.001.

Reason: SR including different types of subjects

186. Carvalho PHA, Moura LB, Trento GS, Holzinger D, Gabrielli MAC, Gabrielli MFR, Pereira Filho VA. Surgically assisted rapid maxillary expansion: a systematic review of complications. Int J Oral Maxillofac Surg. 2020;49:325–32. doi:10.1016/j.ijom.2019.08.011.

Reason: primary in vitro studies were excluded

187. Cavalheiro CP, Scherer H, Imparato JCP, Collares FM, Lenzi TL. Use of flowable resin composite as an intermediate layer in class II restorations: a systematic review and meta-analysis. Clin Oral Investig. 2021;25:5629–39. doi:10.1007/s00784-021-04090-5.

Reason: SR including different types of subjects

188. Coll JA, Vargas K, Marghalani AA, Chen C-Y, AlShamali S, Dhar V, Crystal YO. A Systematic Review and Meta-Analysis of Nonvital Pulp Therapy for Primary Teeth. Pediatr Dent. 2020;42:256–461.

Reason: SR including different types of subjects

189. Cronshaw M, Parker S, Anagnostaki E, Mylona V, Lynch E, Grootveld M. Photobiomodulation Dose Parameters in Dentistry: A Systematic Review and Meta-Analysis. Dent J (Basel) 2020. doi:10.3390/dj8040114.

Reason: SR including different types of subjects

190. da Silva CL, Cavalheiro CP, Gimenez T, Imparato JCP, Bussadori SK, Lenzi TL. Bonding Performance of Universal and Contemporary Adhesives in Primary Teeth: A Systematic Review and Network Meta-Analysis of In Vitro Studies. Pediatr Dent. 2021;43:170–7.

Reason: SR including different types of subjects

191. Fakhruddin KS, Egusa H, Ngo HC, Panduwawala C, Pesee S, Samaranayake LP. Clinical efficacy and the antimicrobial potential of silver formulations in arresting dental caries: a systematic review. BMC Oral Health. 2020;20:160. doi:10.1186/s12903-020-01133-3.

Reason: SR including different types of subjects

192. Fernandez MDS, Guedes MIF, Langa GPJ, Rösing CK, Cavagni J, Muniz FWMG. Virucidal efficacy of chlorhexidine: a systematic review. Odontology 2021. doi:10.1007/s10266-021-00660-x.

Reason: SR including different types of subjects

193. Figueredo CA, Abdelhay N, Figueredo CM, Catunda R, Gibson MP. The impact of vaping on periodontitis: A systematic review. Clin Exp Dent Res. 2021;7:376–84. doi:10.1002/cre2.360.

Reason: SR including different types of subjects

194. Gao WM, Geng W, Yan YW, Wang Y. Antagonist Wear Of Zirconia Fixed Restorations In Vitro And In Vivo- A Systematic Review. Int J Prosthodont. 2021;34:492–504. doi:10.11607/ijp.6984.

Reason: SR including different types of subjects

195. Gareb B, van Bakelen NB, Dijkstra PU, Vissink A, Bos RRM, van Minnen B. Biodegradable versus titanium osteosynthesis in maxillofacial traumatology: a systematic review with meta-analysis and trial sequential analysis. Int J Oral Maxillofac Surg. 2020;49:914–31. doi:10.1016/j.ijom.2019.11.009.

Reason: SR including different types of subjects

196. Gaur S, Agnihotri R. Application of Adipose Tissue Stem Cells in Regenerative Dentistry: A Systematic Review. J Int Soc Prev Community Dent. 2021;11:266–71. doi:10.4103/jispcd.JISPCD_43_21.

Reason: SR including different types of subjects

197. Gizani S, Kloukos D, Papadimitriou A, Roumani T, Twetman S. Is Bleaching Effective in Managing Post-orthodontic White-spot Lesions? A Systematic Review. Oral Health Prev Dent. 2020;18:2–10. doi:10.3290/j.ohpd.a44113.

Reason: SR including different types of subjects

198. Gómez-Aguirre JN, Argueta-Figueroa L, Castro-Gutiérrez MEM, Torres-Rosas R. Effects of interproximal enamel reduction techniques used for orthodontics: A systematic review. Orthod Craniofac Res 2021. doi:10.1111/ocr.12555.

Reason: SR including different types of subjects

199. González FJ, Requena E, Miralles L, Sanz JL, Barberá J, Enciso JJ, et al. Adjuvant Effect of Titanium Brushes in Peri-Implant Surgical Treatment: A Systematic Review. Dent J (Basel) 2021. doi:10.3390/dj9080084.

Reason: primary in vitro studies were excluded

200. Govare N, Contrepois M. Endocrowns: A systematic review. J Prosthet Dent. 2020;123:411-418.e9. doi:10.1016/j.prosdent.2019.04.009.

Reason: SR including different types of subjects

201. Govindaraj A, Dinesh SS. Effect of Chlorhexidine Varnish and Fluoride Varnish on White Spot Lesions in Orthodontic Patients- a Systematic Review. TODENTJ. 2021;15:151–9. doi:10.2174/1874210602115010151.

Reason: SR including different types of subjects

202. Grymak A, Aarts JM, Ma S, Waddell JN, Choi JJE. Wear Behavior of Occlusal Splint Materials Manufactured By Various Methods: A Systematic Review. J Prosthodont 2021. doi:10.1111/jopr.13432.

Reason: SR including different types of subjects

203. Hasanzade, M., Aminikhah, M., Afrashtehfar, K.I., Alikhasi, M. Marginal and internal adaptation of single crowns and fixed dental prostheses by using digital and conventional workflows: A systematic review and meta-analysis. 2021:360–8.

Reason: SR including different types of subjects

204. Hong D-W, Lin X-J, Wiegand A, Yu H. Does delayed toothbrushing after the consumption of erosive foodstuffs or beverages decrease erosive tooth wear? A systematic review and meta-analysis. Clin Oral Investig. 2020;24:4169–83. doi:10.1007/s00784-020-03614-9.

Reason: SR including different types of subjects

205. Hynková K, Sarao SK, Voborná I, Levin L. Effect of assessor's sex on visual color matching in dentistry: A systematic review of the literature. J Esthet Restor Dent 2021. doi:10.1111/jerd.12839.

Reason: SR including different types of subjects

206. Janjic Rankovic M, Kapor S, Khazaei Y, Crispin A, Schüler I, Krause F, et al. Systematic review and meta-analysis of diagnostic studies of proximal surface caries. Clin Oral Investig. 2021;25:6069–79. doi:10.1007/s00784-021-04113-1.

Reason: SR including different types of subjects

207. Jayaraman J, Nagendrababu V, Pulikkotil SJ, Veettil SK, Dhar V. Effectiveness of formocresol and ferric sulfate as pulpotomy material in primary molars: a systematic review and meta-analysis with trial sequential analysis of randomized clinical trials. Quintessence Int. 2020;51:38–48. doi:10.3290/j.qi.a43617.

Reason: SR including different types of subjects

208. Jorba-García A, González-Barnadas A, Camps-Font O, Figueiredo R, Valmaseda-Castellón E. Accuracy assessment of dynamic computer-aided implant placement: a systematic review and meta-analysis. Clin Oral Investig. 2021;25:2479–94. doi:10.1007/s00784-021-03833-8.

Reason: SR including different types of subjects

209. Josic U, Sebold M, Lins RBE, Savovic J, Mazzitelli C, Maravic T, et al. Does immediate dentin sealing influence postoperative sensitivity in teeth restored with indirect restorations? A systematic review and meta-analysis. J Esthet Restor Dent 2021. doi:10.1111/jerd.12841.

Reason: SR including different types of subjects

210. Kachhara S, Nallaswamy D, Ganapathy DM, Sivaswamy V, Rajaraman V. Assessment of intraoral scanning technology for multiple implant impressions - A systematic review and meta-analysis. J Indian Prosthodont Soc. 2020;20:141–52. doi:10.4103/jips.jips_379_19.

Reason: SR including different types of subjects

211. Kapor S, Rankovic MJ, Khazaei Y, Crispin A, Schüler I, Krause F, et al. Systematic review and meta-analysis of diagnostic methods for occlusal surface caries. Clin Oral Investig. 2021;25:4801–15. doi:10.1007/s00784-021-04024-1.

Reason: SR including different types of subjects

212. Khan AR, Fida M, Gul M. Decalcification and bond failure rate in resin modified glass ionomer cement versus conventional composite for orthodontic bonding: A systematic review & meta-analysis. Int Orthod. 2020;18:32–40. doi:10.1016/j.ortho.2019.10.003.

Reason: SR including different types of subjects

213. Khouly I, Braun RS, Silvestre T, Musa W, Miron RJ, Demyati A. Efficacy of antibiotic prophylaxis in intraoral bone grafting procedures: a systematic review and meta-analysis. Int J Oral Maxillofac Surg. 2020;49:250–63. doi:10.1016/j.ijom.2019.07.003.

Reason: SR including different types of subjects

214. Limeback H, Enax J, Meyer F. Biomimetic hydroxyapatite and caries prevention: a systematic review and meta-analysis. Can J Dent Hyg. 2021;55:148–59.

Reason: SR including different types of subjects

215. Lin Z, Strauss FJ, Lang NP, Sculean A, Salvi GE, Stähli A. Efficacy of laser monotherapy or non-surgical mechanical instrumentation in the management of untreated periodontitis patients. A systematic review and meta-analysis. Clin Oral Investig. 2021;25:375–91. doi:10.1007/s00784-020-03584-y.

Reason: SR including different types of subjects

216. Maru V, Dixit U, Patil RSB, Parekh R. Cytotoxicity and Bioactivity of Mineral Trioxide Aggregate and Bioactive Endodontic Type Cements: A Systematic Review. Int J Clin Pediatr Dent. 2021;14:30–9. doi:10.5005/jp-journals-10005-1880.

Reason: SR including different types of subjects

217. Morsy N, El Kateb M, Azer A, Fathalla S. Fit of zirconia fixed partial dentures fabricated from conventional impressions and digital scans: A systematic review and meta-analysis. J Prosthet Dent 2021. doi:10.1016/j.prosdent.2021.08.025.

Reason: SR including different types of subjects

218. Nithyanandham, S., Sivaswamy, V., Thangavelu, L. Evaluation Of The Clinical Effectiveness Of The Conometric Concept - A Systematic Review. IJDOS. 2014:2918–23. doi:10.19070/2377-8075-21000572.

Reason: SR including different types of subjects

219. Nivedhitha S. Comparing The Effectiveness Of Various Irrigant Activation Techniques With Conventional Needle Irrigation - A Systematic Review. IJDOS. 2014:2626–31. doi:10.19070/2377-8075-21000514.

Reason: SR including different types of subjects

220. Ortiz MIG, Melo Alencar C de, Paula BLF de, Magno MB, Maia LC, Silva CM. Accuracy of near-infrared light transillumination (NILT) compared to bitewing radiograph for detection of interproximal caries in the permanent dentition: A systematic review and meta-analysis. J Dent. 2020;98:103351. doi:10.1016/j.jdent.2020.103351.

Reason: SR including different types of subjects

221. Pan Y, Tsoi JKH, Lam WYH, Pow EHN. Implant framework misfit: A systematic review on assessment methods and clinical complications. Clin Implant Dent Relat Res. 2021;23:244–58. doi:10.1111/cid.12968.

Reason: SR including different types of subjects

222. Paños-Crespo A, Sánchez-Torres A, Gay-Escoda C. Retrograde filling material in periapical surgery: a systematic review. Med Oral Patol Oral Cir Bucal. 2021;26:e422-e429. doi:10.4317/medoral.24262.

Reason: SR including different types of subjects

223. Papaspyridakos P, Vazouras K, Chen Y-W, Kotina E, Natto Z, Kang K, Chochlidakis K. Digital vs Conventional Implant Impressions: A Systematic Review and Meta-Analysis. J Prosthodont. 2020;29:660–78. doi:10.1111/jopr.13211.

Reason: SR including different types of subjects

224. Pordeus MD, Santiago Junior JF, Venante HS, Da Bringel Costa RM, Chappuis Chocano AP, Porto VC. Computer-aided technology for fabricating removable partial denture frameworks: A systematic review and meta-analysis. J Prosthet Dent 2021. doi:10.1016/j.prosdent.2020.06.006.

Reason: SR including different types of subjects

225. PradeepKumar AR, Shemesh H, Nivedhitha MS, Hashir MMJ, Arockiam S, Uma Maheswari TN, Natanasabapathy V. Diagnosis of Vertical Root Fractures by Cone-beam Computed Tomography in Root-filled Teeth with Confirmation by Direct Visualization: A Systematic Review and Meta-Analysis. J Endod. 2021;47:1198–214. doi:10.1016/j.joen.2021.04.022.

Reason: primary in vitro studies were excluded

226. Quigley NP, Loo DSS, Choy C, Ha WN. Clinical efficacy of methods for bonding to zirconia: A systematic review. J Prosthet Dent. 2021;125:231–40. doi:10.1016/j.prosdent.2019.12.017.

Reason: SR including different types of subjects

227. Rajwani AR, Hawes SND, To A, Quaranta A, Rincon Aguilar JC. Effectiveness of Manual Toothbrushing Techniques on Plaque and Gingivitis: A Systematic Review. Oral Health Prev Dent. 2020;18:843–54. doi:10.3290/j.ohpd.a45354.

Reason: SR including different types of subjects

228. Romo-Huerta MJ, Del Cervantes-Urenda AR, Velasco-Neri J, Torres-Bugarín O, Valdivia ADCM. Genotoxicity Associated with Residual Monomers in Restorative Dentistry: A Systematic Review. Oral Health Prev Dent. 2021;19:471–80. doi:10.3290/j.ohpd.b2081469.

Reason: SR including different types of subjects

229. Sabour A, El Helou M, Roger-Leroi V, Bauer C. Release and toxicity of bisphenol-A (BPA) contained in orthodontic adhesives: A systematic review. Int Orthod. 2021;19:1–14. doi:10.1016/j.ortho.2020.11.002.

Reason: SR including different types of subjects

230. Salvi GE, Stähli A, Schmidt JC, Ramseier CA, Sculean A, Walter C. Adjunctive laser or antimicrobial photodynamic therapy to non-surgical mechanical instrumentation in patients with untreated periodontitis: A systematic review and meta-analysis. J Clin Periodontol. 2020;47 Suppl 22:176–98. doi:10.1111/jcpe.13236.

Reason: SR including different types of subjects

231. Sanches IB, Metzker TC, Kappler R, Oliveira MV, Carvalho AO, Castor Xisto Lima EM. Marginal adaptation of CAD-CAM and heat-pressed lithium disilicate crowns: A systematic review and meta-analysis. J Prosthet Dent 2021. doi:10.1016/j.prosdent.2021.03.021.

Reason: SR including different types of subjects

232. Sarafidou K, Vasileiadi G, Louvrou MK, Moldovani E, Koidis P, Kokoti M, Bakopoulou A. Screwmentable implant-supported prostheses: A systematic review. J Prosthet Dent 2021. doi:10.1016/j.prosdent.2021.08.027.

Reason: SR including different types of subjects

233. Schmutzler A, Rauch A, Nitschke I, Lethaus B, Hahnel S. CLEANING OF REMOVABLE DENTAL PROSTHESES - A SYSTEMATIC REVIEW. J Evid Based Dent Pract. 2021;21:101644. doi:10.1016/j.jebdp.2021.101644.

Reason: SR including different types of subjects

234. Sousa CA de, Taborda MBB, Momesso GAC, Rocha EP, Dos Santos PH, Santiago-Júnior JF, Assunção WG. Materials Sealing Preventing Biofilm Formation in Implant/Abutment Joints: Which Is the Most Effective? A Systematic Review and Meta-Analysis. J Oral Implantol. 2020;46:163–71. doi:10.1563/aaid-joi-D-19-00121.

Reason: SR including different types of subjects

235. Srinivasan M, Kamnoedboon P, McKenna G, Angst L, Schimmel M, Özcan M, Müller F. CAD-CAM removable complete dentures: A systematic review and meta-analysis of

trueness of fit, biocompatibility, mechanical properties, surface characteristics, color stability, time-cost analysis, clinical and patient-reported outcomes. J Dent. 2021;113:103777. doi:10.1016/j.jdent.2021.103777.

Reason: SR including different types of subjects

236. Tieh MT, Waddell JN, Choi JJE. Optical Properties and Color Stability of Denture Teeth-A Systematic Review. J Prosthodont 2021. doi:10.1111/jopr.13429.

Reason: SR including different types of subjects

237. Tonprasong W, Inokoshi M, Shimizubata M, Yamamoto M, Hatano K, Minakuchi S. Impact of direct restorative dental materials on surface root caries treatment. Evidence based and current materials development: A systematic review. Jpn Dent Sci Rev. 2022;58:13–30. doi:10.1016/j.jdsr.2021.11.004.

Reason: SR including different types of subjects

238. van Acker JWG, Pauwels NS, Cauwels RGEC, Rajasekharan S. Outcomes of different radioprotective precautions in children undergoing dental radiography: a systematic review. Eur Arch Paediatr Dent. 2020;21:463–508. doi:10.1007/s40368-020-00544-8.

Reason: SR including different types of subjects

239. Verma SK, Dev Kumar B, Chaurasia A, Dubey D. Effectiveness of mouthwash against viruses: 2020 perspective. A systematic review. Minerva Dent Oral Sci. 2021;70:206–13. doi:10.23736/S2724-6329.21.04418-6.

Reason: SR including different types of subjects

240. Wang F, Li C, Zhang S, Liu H. Role of TiO2 Nanotubes on the Surface of Implants in Osseointegration in Animal Models: A Systematic Review and Meta-Analysis. J Prosthodont. 2020;29:501–10. doi:10.1111/jopr.13163.

Reason: SR including different types of subjects

241. Wiegand A, Lechte C, Kanzow P. Adhesion to eroded enamel and dentin: systematic review and meta-analysis. Dent Mater. 2021;37:1845–53. doi:10.1016/j.dental.2021.09.014.

Reason: SR including different types of subjects

242. Wulfman C, Naveau A, Rignon-Bret C. Digital scanning for complete-arch implant-supported restorations: A systematic review. J Prosthet Dent. 2020;124:161–7. doi:10.1016/j.prosdent.2019.06.014.

Reason: SR including different types of subjects

243. Yu X, Han Y, Wang J. Is an internal tapered connection more efficient than an internal nontapered connection? A systematic review and meta-analysis. J Prosthet Dent. 2020;124:431–8. doi:10.1016/j.prosdent.2019.07.018.

Reason: SR including different types of subjects

**Additional file 6.** List of excluded articles after full-text assessment

1. Abdullah N, Al-Marzooq F, Mohamad S, Abd Rahman N, Chi Ngo H, Perera Samaranayake L. Intraoral appliances for in situ oral biofilm growth: a systematic review. J Oral Microbiol. 2019;11:1647757. doi:10.1080/20002297.2019.1647757.

Reason: SR including different types of subjects

2. Abduo J, Yin L. Fits of Implant Zirconia Custom Abutments and Frameworks: A Systematic Review and Meta-Analyses. Int J Oral Maxillofac Implants. 2019;34:99–114. doi:10.11607/jomi.6657.

Reason: Unclear type of study design included

3. Atieh MA, Alsabeeha N, Duncan WJ. Stability of tapered and parallel-walled dental implants: A systematic review and meta-analysis. Clinical implant dentistry and related research. 2018;20:634–45. doi:10.1111/cid.12623.

Reason: SR including different types of subjects

4. Attin T, Hannig C, Wiegand A, Attin R. Effect of bleaching on restorative materials and restorations--a systematic review. Dent Mater. 2004;20:852–61. doi:10.1016/j.dental.2004.04.002.

Reason: Unclear type of study design included

5. Azarpazhooh A, Main PA. Is there a risk of harm or toxicity in the placement of pit and fissure sealant materials? A systematic review. J Can Dent Assoc. 2008;74:179–83.

Reason: SR including different types of subjects

6. Bader JD, Shugars DA. A systematic review of the performance of a laser fluorescence device for detecting caries. J Am Dent Assoc. 2004;135:1413–26. doi:10.14219/jada.archive.2004.0051.

Reason: SR including different types of subjects

7. Bader JD, Shugars DA, Bonito AJ. A systematic review of the performance of methods for identifying carious lesions. Journal of public health dentistry. 2002;62:201–13. doi:10.1111/j.1752-7325.2002.tb03446.x.

Reason: SR including different types of subjects

8. Batista VEdS, Santiago Junior JF, Almeida DAdF, Lopes LFdTP, Verri FR, Pellizzer EP. The effect of offset implant configuration on bone stress distribution: a systematic review. J Prosthodont. 2015;24:93–9. doi:10.1111/jopr.12221.

Reason: Unclear type of study design included

9. Behdin S, Monje A, Lin G-H, Edwards B, Othman A, Wang H-L. Effectiveness of Laser Application for Periodontal Surgical Therapy: Systematic Review and Meta-Analysis. Journal of periodontology. 2015;86:1352–63. doi:10.1902/jop.2015.150212.

Reason: SR including different types of subjects

10. Bellinaso MD, Soares FZM, Rocha RdO. Do bulk-fill resins decrease the restorative time in posterior teeth? A systematic review and meta-analysis of in vitro studies. J Investig Clin Dent. 2019;10:e12463. doi:10.1111/jicd.12463.

Reason: SR including different types of subjects

11. Berchier CE, Slot DE, van der Weijden GA. The efficacy of 0.12% chlorhexidine mouthrinse compared with 0.2% on plaque accumulation and periodontal parameters: a systematic review. J Clin Periodontol. 2010;37:829–39. doi:10.1111/j.1600-051X.2010.01575.x.

Reason: SR including different types of subjects

12. Bohner L, Gamba DD, Hanisch M, Marcio BS, Tortamano Neto P, Laganá DC, Sesma N. Accuracy of digital technologies for the scanning of facial, skeletal, and intraoral tissues: A systematic review. J Prosthet Dent. 2019;121:246–51. doi:10.1016/j.prosdent.2018.01.015.

Reason: SR including different types of subjects

13. Braga RR, Ballester RY, Ferracane JL. Factors involved in the development of polymerization shrinkage stress in resin-composites: a systematic review. Dent Mater. 2005;21:962–70. doi:10.1016/j.dental.2005.04.018.

Reason: Unclear type of study design included

14. Brian HC, Lam OL, Jagannathan N, Botelho MG. A Systematic Review of Amalgam Bonded Restorations: In vitro and Clinical Findings. J Contemp Dent Pract. 2018;19:1013–24.

Reason: SR including different types of subjects

15. Bühler J, Amato M, Weiger R, Walter C. A systematic review on the effects of air polishing devices on oral tissues. Int J Dent Hyg. 2016;14:15–28. doi:10.1111/idh.12120.

Reason: SR including different types of subjects

16. CERUSO FM, BARNABA P, MAZZOLENI S, OTTRIA L, GARGARI M, ZUCCON A, et al. Implant-abutment connections on single crowns: a systematic review. Oral Implantol (Rome). 2017;10:349–53. doi:10.11138/orl/2017.10.4.349.

Reason: SR including different types of subjects

17. Chisini LA, Conde MCM, Grazioli G, Martin ASS, Carvalho RV de, Nör JE, Demarco FF. Venous Blood Derivatives as FBS-Substitutes for Mesenchymal Stem Cells: A Systematic Scoping Review. Braz Dent J. 2017;28:657–68. doi:10.1590/0103-6440201701646.

Reason: SR including different types of subjects

18. Chochlidakis KM, Papaspyridakos P, Geminiani A, Chen C-J, Feng IJ, Ercoli C. Digital versus conventional impressions for fixed prosthodontics: A systematic review and meta-analysis. J Prosthet Dent. 2016;116:184-190.e12. doi:10.1016/j.prosdent.2015.12.017.

Reason: SR including different types of subjects

19. Choi IGG, Cortes ARG, Arita ES, Georgetti MAP. Comparison of conventional imaging techniques and CBCT for periodontal evaluation: A systematic review. Imaging Sci Dent. 2018;48:79–86. doi:10.5624/isd.2018.48.2.79.

Reason: SR including different types of subjects

20. Cocco AR, Da Rosa WLdO, Da Silva AF, Lund RG, Piva E. A systematic review about antibacterial monomers used in dental adhesive systems: Current status and further prospects. Dent Mater. 2015;31:1345–62. doi:10.1016/j.dental.2015.08.155.

Reason: SR including different types of subjects

21. Conde MCM, Chisini LA, Demarco FF, Nör JE, Casagrande L, Tarquinio SBC. Stem cell-based pulp tissue engineering: variables enrolled in translation from the bench to the bedside, a systematic review of literature. Int Endod J. 2016;49:543–50. doi:10.1111/iej.12489.

Reason: SR including different types of subjects

22. Conejo J, Kobayashi T, Anadioti E, Blatz MB. Performance of CAD/CAM monolithic ceramic Implant-supported restorations bonded to titanium inserts: A systematic review. Eur J Oral Implantol. 2017;10 Suppl 1:139–46.

Reason: SR including different types of subjects

23. Corega C, Vaida L, Festila DG, Rigoni G, Albanese M, D'Agostino A, et al. The benefits of Quercitin for dentistry and maxillofacial surgery: a systematic review. Minerva Stomatol. 2014.

Reason: SR including different types of subjects

24. Donnermeyer D, Bürklein S, Dammaschke T, Schäfer E. Endodontic sealers based on calcium silicates: a systematic review. Odontology. 2019;107:421–36. doi:10.1007/s10266-018-0400-3.

Reason: SR including different types of subjects

25. Duggal M, Tong HJ, Al-Ansary M, Twati W, Day PF, Nazzal H. Interventions for the endodontic management of non-vital traumatised immature permanent anterior teeth in children and adolescents: a systematic review of the evidence and guidelines of the European Academy of Paediatric Dentistry. Eur Arch Paediatr Dent. 2017;18:139–51. doi:10.1007/s40368-017-0289-5.

Reason: SR including different types of subjects

26. Ekstrand KR, Gimenez T, Ferreira FR, Mendes FM, Braga MM. The International Caries Detection and Assessment System - ICDAS: A Systematic Review. Caries research. 2018;52:406–19. doi:10.1159/000486429.

Reason: SR not related to interventions

27. Euler de Souza Lucena E, Guzen FP, Lopes de Paiva Cavalcanti JR, Galvão Barboza CA, Silva do Nascimento Júnior E, Cavalcante JdS. Experimental considerations concerning the use of stem cells and tissue engineering for facial nerve regeneration: a systematic review. J Oral Maxillofac Surg. 2014;72:1001–12. doi:10.1016/j.joms.2013.11.006.

Reason: SR including different types of subjects

28. Eweida AM, Horch RE, Marei MK, Elhammady HA, Etaby AN, Nabawi AS, Sakr MF. Axially vascularised mandibular constructs: Is it time for a clinical trial? J Craniomaxillofac Surg. 2015;43:1028–32. doi:10.1016/j.jcms.2014.10.018.

Reason: SR including different types of subjects

29. Ferrúa CP, Centeno EGZ, Da Rosa LC, Amaral CCd, Severo RF, Sarkis-Onofre R, et al. How has dental pulp stem cells isolation been conducted? A scoping review. Braz Oral Res. 2017;31:e87. doi:10.1590/1807-3107BOR-2017.vol31.0087.

Reason: SR not related to interventions

30. Fokkinga WA, Creugers NH, Kreulen CM. In vitro failure mode of fiber-reinforced post-core systems: A systematic review. 2003;82:B331.

Reason: Full-text not available

31. Fukushima KA, Marques MM, Tedesco TK, Carvalho GL, Gonçalves F, Caballero-Flores H, et al. Screening of hydrogel-based scaffolds for dental pulp regeneration-A systematic review. Arch Oral Biol. 2019;98:182–94. doi:10.1016/j.archoralbio.2018.11.023.

Reason: SR including different types of subjects

32. Ghazali N, Shaw RJ, Rogers SN, Risk JM. Genomic determinants of normal tissue toxicity after radiotherapy for head and neck malignancy: a systematic review. Oral Oncol. 2012;48:1090–100. doi:10.1016/j.oraloncology.2012.08.002.

Reason: SR including different types of subjects

33. Gou M, Chen H, Fu M, Wang H. Fracture of Zirconia Abutments in Implant Treatments: A Systematic Review. Implant Dent. 2019;28:378–87. doi:10.1097/ID.0000000000000900.

Reason: SR including different types of subjects

34. Graziani F, Cei S, Ivanovski S, La Ferla F, Gabriele M. A systematic review of the effectiveness of bone collectors. Int J Oral Maxillofac Implants. 2007;22:729–35.

Reason: SR including different types of subjects

35. Grischke J, Eberhard J, Stiesch M. Antimicrobial dental implant functionalization strategies -A systematic review. Dent Mater J. 2016;35:545–58. doi:10.4012/dmj.2015-314.

Reason: SR including different types of subjects

36. Haas LF, Zimmermann GS, Luca Canto G de, Flores-Mir C, Corrêa M. Precision of cone beam CT to assess periodontal bone defects: a systematic review and meta-analysis. Dentomaxillofac Radiol. 2018;47:20170084. doi:10.1259/dmfr.20170084.

Reason: SR including different types of subjects

37. Halimi A, Benyahia H, Bahije L, Adli H, Azeroual M-F, Zaoui F. A systematic study of the release of bisphenol A by orthodontic materials and its biological effects. Int Orthod. 2016;14:399–417. doi:10.1016/j.ortho.2016.10.005.

Reason: SR including different types of subjects

38. Heintze SD, Cavalleri A, Forjanic M, Zellweger G, Rousson V. Wear of ceramic and antagonist--a systematic evaluation of influencing factors in vitro. Dent Mater. 2008;24:433–49. doi:10.1016/j.dental.2007.06.016.

Reason: SR including different types of subjects

39. Iglesias-Linares A, Yáñez-Vico R-M, Solano-Reina E, Torres-Lagares D, González Moles MA. Influence of bisphosphonates in orthodontic therapy: Systematic review. J Dent. 2010;38:603–11. doi:10.1016/j.jdent.2010.05.012.

Reason: SR including different types of subjects

40. Iqbal Z, Zafar MS. Role of antifungal medicaments added to tissue conditioners: A systematic review. J Prosthodont Res. 2016;60:231–9. doi:10.1016/j.jpor.2016.03.006.

Reason: SR including different types of subjects

41. Janssen NG, Weijs WLJ, Koole R, Rosenberg AJWP, Meijer GJ. Tissue engineering strategies for alveolar cleft reconstruction: a systematic review of the literature. Clin Oral Investig. 2014;18:219–26. doi:10.1007/s00784-013-0947-x.

Reason: SR including different types of subjects

42. Jegoux F, Malard O, Goyenvalle E, Aguado E, Daculsi G. Radiation effects on bone healing and reconstruction: interpretation of the literature. Oral Surg Oral Med Oral Pathol Oral Radiol Endod. 2010;109:173–84. doi:10.1016/j.tripleo.2009.10.001.

Reason: SR including different types of subjects

43. Kamal M, Ziyab AH, Bartella A, Mitchell D, Al-Asfour A, Hölzle F, et al. Volumetric comparison of autogenous bone and tissue-engineered bone replacement materials in alveolar cleft repair: a systematic review and meta-analysis. Br J Oral Maxillofac Surg. 2018;56:453–62. doi:10.1016/j.bjoms.2018.05.007.

Reason: SR including different types of subjects

44. Kolahi J, Soolari A. Rinsing with chlorhexidine gluconate solution after brushing and flossing teeth: a systematic review of effectiveness. Quintessence Int. 2006;37:605–12.

Reason: SR including different types of subjects

45. Koretsi V, Chatzigianni A, Sidiropoulou S. Enamel roughness and incidence of caries after interproximal enamel reduction: a systematic review. Orthodontics & craniofacial research. 2014;17:1–13. doi:10.1111/ocr.12030.

Reason: SR including different types of subjects

46. Leonardi Dutra K, Haas L, Porporatti AL, Flores-Mir C, Nascimento Santos J, Mezzomo LA, et al. Diagnostic Accuracy of Cone-beam Computed Tomography and Conventional Radiography on Apical Periodontitis: A Systematic Review and Meta-analysis. J Endod. 2016;42:356–64. doi:10.1016/j.joen.2015.12.015.

Reason: SR including different types of subjects

47. Li S, Schmalz G, Schmidt J, Krause F, Haak R, Ziebolz D. Antimicrobial peptides as a possible interlink between periodontal diseases and its risk factors: A systematic review. J Periodontal Res. 2018;53:145–55. doi:10.1111/jre.12482.

Reason: SR including different types of subjects

48. Liedke GS, Spin-Neto R, da Silveira HED, Wenzel A. Radiographic diagnosis of dental restoration misfit: a systematic review. J Oral Rehabil. 2014;41:957–67. doi:10.1111/joor.12215.

Reason: SR including different types of subjects

49. Lizzi F, Villat C, Attik N, Jackson P, Grosgogeat B, Goutaudier C. Mechanical characteristic and biological behaviour of implanted and restorative bioglasses used in medicine and dentistry: A systematic review. Dent Mater. 2017;33:702–12. doi:10.1016/j.dental.2017.03.017.

Reason: SR including different types of subjects

50. Louropoulou A, Slot DE, van der Weijden FA. Titanium surface alterations following the use of different mechanical instruments: a systematic review. Clin Oral Implants Res. 2012;23:643–58. doi:10.1111/j.1600-0501.2011.02208.x.

Reason: SR including different types of subjects

51. Louropoulou A, Slot DE, van der Weijden F. The effects of mechanical instruments on contaminated titanium dental implant surfaces: a systematic review. Clin Oral Implants Res. 2014;25:1149–60. doi:10.1111/clr.12224.

Reason: SR including different types of subjects

52. Metsälä E, Henner A, Ekholm M. Quality assurance in digital dental imaging: a systematic review. Acta Odontol Scand. 2014;72:362–71. doi:10.3109/00016357.2013.840736.

Reason: SR including different types of subjects

53. Mishra S, Chowdhary R. PEEK materials as an alternative to titanium in dental implants: A systematic review. Clinical implant dentistry and related research. 2019;21:208–22. doi:10.1111/cid.12706.

Reason: SR including different types of subjects

54. Mizumoto RM, Yilmaz B. Intraoral scan bodies in implant dentistry: A systematic review. J Prosthet Dent. 2018;120:343–52. doi:10.1016/j.prosdent.2017.10.029.

Reason: Unclear type of study design included

55. Möhlhenrich SC, Modabber A, Steiner T, Mitchell DA, Hölzle F. Heat generation and drill wear during dental implant site preparation: systematic review. Br J Oral Maxillofac Surg. 2015;53:679–89. doi:10.1016/j.bjoms.2015.05.004.

Reason: SR including different types of subjects

56. Montagner AF, Sarkis-Onofre R, Pereira-Cenci T, Cenci MS. MMP Inhibitors on Dentin Stability: A Systematic Review and Meta-analysis. J Dent Res. 2014;93:733–43. doi:10.1177/0022034514538046.

Reason: Unclear type of study design included

57. Moreno-Rabié C, Torres A, Lambrechts P, Jacobs R. Clinical applications, accuracy and limitations of guided endodontics: a systematic review. Int Endod J. 2020;53:214–31. doi:10.1111/iej.13216.

Reason: SR including different types of subjects

58. Neldam CA, Pinholt EM. Synchrotron µCT imaging of bone, titanium implants and bone substitutes - a systematic review of the literature. J Craniomaxillofac Surg. 2014;42:801–5. doi:10.1016/j.jcms.2013.11.015.

Reason: SR including different types of subjects

59. Nikolic-Jakoba N, Spin-Neto R, Wenzel A. Cone-Beam Computed Tomography for Detection of Intrabony and Furcation Defects: A Systematic Review Based on a Hierarchical Model for Diagnostic Efficacy. Journal of periodontology. 2016;87:630–44. doi:10.1902/jop.2016.150636.

Reason: SR including different types of subjects

60. Nilsen BW, Örtengren U, Simon-Santamaria J, Sørensen KK, Michelsen VB. Methods and terminology used in cell-culture studies of low-dose effects of matrix constituents of polymer resin-based dental materials. Eur J Oral Sci. 2016;124:511–25. doi:10.1111/eos.12309.

Reason: SR not related to interventions

61. Nuvvula S, Bhumireddy JR, Kamatham R, Mallineni SK. Diagnostic accuracy of direct digital radiography and conventional radiography for proximal caries detection in primary teeth: A systematic review. J Indian Soc Pedod Prev Dent. 2016;34:300–5. doi:10.4103/0970-4388.191406.

Reason: SR including different types of subjects

62. Oliveira TT, Reis AC. Fabrication of dental implants by the additive manufacturing method: A systematic review. J Prosthet Dent. 2019;122:270–4. doi:10.1016/j.prosdent.2019.01.018.

Reason: SR including different types of subjects

63. Oliveira NG de, Souza Araújo PR de, da Silveira MT, Sobral APV, Carvalho MdV. Comparison of the biocompatibility of calcium silicate-based materials to mineral trioxide aggregate: Systematic review. Eur J Dent. 2018;12:317–26. doi:10.4103/ejd.ejd_347_17.

Reason: SR not related to interventions

64. Pablo OV de, Estevez R, Péix Sánchez M, Heilborn C, Cohenca N. Root anatomy and canal configuration of the permanent mandibular first molar: a systematic review. J Endod. 2010;36:1919–31. doi:10.1016/j.joen.2010.08.055.

Reason: SR including different types of subjects

65. Papageorgiou SN, Konstantinidis I, Papadopoulou K, Jäger A, Bourauel C. A systematic review and meta-analysis of experimental clinical evidence on initial aligning archwires and archwire sequences. Orthodontics & craniofacial research. 2014;17:197–215. doi:10.1111/ocr.12048.

Reason: SR including different types of subjects

66. Pauli Paglioni M de, Araújo ALD, Arboleda PA, Palmier NR, Fonsêca JM, Gomes-Silva W, et al. Tumor safety and side effects of photobiomodulation therapy used for prevention and management of cancer treatment toxicities. A systematic review. Oral Oncol. 2019;93:21–8. doi:10.1016/j.oraloncology.2019.04.004.

Reason: SR including different types of subjects

67. Pérez-López D, Varela-Centelles P, García-Pola M-J, Castelo-Baz P, García-Caballero L, Seoane-Romero J-M. Oral mucosal peeling related to dentifrices and mouthwashes: A systematic review. Med Oral Patol Oral Cir Bucal. 2019;24:e452-e460. doi:10.4317/medoral.22939.

Reason: SR including different types of subjects

68. Randall RC, Wilson NH. Glass-ionomer restoratives: a systematic review of a secondary caries treatment effect. J Dent Res. 1999;78:628–37. doi:10.1177/00220345990780020101.

Reason: SR including different types of subjects

69. Rathinam E, Rajasekharan S, Chitturi RT, Martens L, Coster P de. Gene Expression Profiling and Molecular Signaling of Dental Pulp Cells in Response to Tricalcium Silicate Cements: A Systematic Review. J Endod. 2015;41:1805–17. doi:10.1016/j.joen.2015.07.015.

Reason: SR including different types of subjects

70. Rathinam E, Rajasekharan S, Chitturi RT, Declercq H, Martens L, Coster P de. Gene Expression Profiling and Molecular Signaling of Various Cells in Response to Tricalcium Silicate Cements: A Systematic Review. J Endod. 2016;42:1713–25. doi:10.1016/j.joen.2016.08.027.

Reason: SR including different types of subjects

71. Razdan A, Benetti AR, Bjørndal L. Do in vitro solubility studies on endodontic sealers demonstrate a high level of evidence? A systematic review. Acta Odontol Scand. 2019;77:253–63. doi:10.1080/00016357.2018.1538535.

Reason: SR not related to interventions

72. Sanz-Martín I, Sanz-Sánchez I, Carrillo de Albornoz A, Figuero E, Sanz M. Effects of modified abutment characteristics on peri-implant soft tissue health: A systematic review and meta-analysis. Clin Oral Implants Res. 2018;29:118–29. doi:10.1111/clr.13097.

Reason: SR including different types of subjects

73. Sarkis-Onofre R, Pereira-Cenci T, Tricco AC, Demarco FF, Moher D, Cenci MS. Systematic reviews in restorative dentistry: discussing relevant aspects. Journal of esthetic and restorative dentistry : official publication of the American Academy of Esthetic Dentistry … [et al.]. 2019;31:222–32. doi:10.1111/jerd.12463.

Reason: SR including different types of subjects

74. Savoldi F, Papoutsi A, Dianiskova S, Dalessandri D, Bonetti S, Tsoi JKH, et al. Resistance to sliding in orthodontics: misconception or method error? A systematic review and a proposal of a test protocol. Korean J Orthod. 2018;48:268–80. doi:10.4041/kjod.2018.48.4.268.

Reason: SR not related to interventions

75. Schwarz F, Aoki A, Becker J, Sculean A. Laser application in non-surgical periodontal therapy: a systematic review. J Clin Periodontol. 2008;35:29–44. doi:10.1111/j.1600-051X.2008.01259.x.

Reason: SR including different types of subjects

76. Shamszadeh S, Asgary S, Nosrat A. Regenerative Endodontics: A Scientometric and Bibliometric Analysis. J Endod. 2019;45:272–80. doi:10.1016/j.joen.2018.11.010.

Reason: SR including different types of subjects

77. Shanbhag S, Shanbhag V. Clinical applications of cell-based approaches in alveolar bone augmentation: a systematic review. Clinical implant dentistry and related research. 2015;17 Suppl 1:e17-34. doi:10.1111/cid.12103.

Reason: SR including different types of subjects

78. Sharma G, Ahmed HMA, Zilm PS, Rossi-Fedele G. Antimicrobial properties of calcium hydroxide dressing when used for long-term application: A systematic review. Australian endodontic journal : the journal of the Australian Society of Endodontology Inc. 2018;44:60–5. doi:10.1111/aej.12216.

Reason: SR including different types of subjects

79. Sherif S, Susarla HK, Kapos T, Munoz D, Chang BM, Wright RF. A systematic review of screw- versus cement-retained implant-supported fixed restorations. J Prosthodont. 2014;23:1–9. doi:10.1111/jopr.12128.

Reason: SR including different types of subjects

80. Silva Almeida LH, Moraes RR, Morgental RD, Pappen FG. Are Premixed Calcium Silicate-based Endodontic Sealers Comparable to Conventional Materials? A Systematic Review of In Vitro Studies. J Endod. 2017;43:527–35. doi:10.1016/j.joen.2016.11.019.

Reason: SR including different types of subjects

81. Smektała T, Jędrzejewski M, Szyndel J, Sporniak-Tutak K, Olszewski R. Experimental and clinical assessment of three-dimensional cephalometry: a systematic review. J Craniomaxillofac Surg. 2014;42:1795–801. doi:10.1016/j.jcms.2014.06.017.

Reason: SR including different types of subjects

82. Sousa CA de, Taborda MBB, Momesso GAC, Rocha EP, Dos Santos PH, Santiago-Júnior JF, Assunção WG. Materials Sealing Preventing Biofilm Formation in Implant/Abutment Joints: Which Is the Most Effective? A Systematic Review and Meta-Analysis. J Oral Implantol. 2020;46:163–71. doi:10.1563/aaid-joi-D-19-00121.

Reason: SR including different types of subjects

83. Tahmaseb A, Wismeijer D, Coucke W, Derksen W. Computer technology applications in surgical implant dentistry: a systematic review. Int J Oral Maxillofac Implants. 2014;29 Suppl:25–42. doi:10.11607/jomi.2014suppl.g1.2.

Reason: SR including different types of subjects

84. Talwar S, Utneja S, Nawal RR, Kaushik A, Srivastava D, Oberoy SS. Role of Cone-beam Computed Tomography in Diagnosis of Vertical Root Fractures: A Systematic Review and Meta-analysis. J Endod. 2016;42:12–24. doi:10.1016/j.joen.2015.09.012.

Reason: SR including different types of subjects

85. Twetman S, Axelsson S, Dahlén G, Espelid I, Mejàre I, Norlund A, Tranæus S. Adjunct methods for caries detection: a systematic review of literature. Acta Odontol Scand. 2013;71:388–97. doi:10.3109/00016357.2012.690448.

Reason: SR including different types of subjects

86. Vaillant-Corroy A-S, Corne P, March P de, Fleutot S, Cleymand F. Influence of recasting on the quality of dental alloys: A systematic review. J Prosthet Dent. 2015;114:205-11.e3. doi:10.1016/j.prosdent.2015.02.004.

Reason: SR including different types of subjects

87. Vechiato-Filho AJ, Pesqueira AA, Souza GM de, dos Santos DM, Pellizzer EP, Goiato MC. Are Zirconia Implant Abutments Safe and Predictable in Posterior Regions? A Systematic Review and Meta-Analysis. Int J Prosthodont. 2016;29:233–44. doi:10.11607/ijp.4349.

Reason: SR including different types of subjects

88. Verma UP, Yadav RK, Dixit M, Gupta A. Platelet-rich Fibrin: A Paradigm in Periodontal Therapy - A Systematic Review. J Int Soc Prev Community Dent. 2017;7:227–33. doi:10.4103/jispcd.JISPCD_429_16.

Reason: SR including different types of subjects

89. Xuereb M, Camilleri J, Attard NJ. Systematic review of current dental implant coating materials and novel coating techniques. Int J Prosthodont. 2015;28:51–9. doi:10.11607/ijp.4124.

Reason: SR including different types of subjects

90. Bianchini LD, Bruna Muhlinberg Vetromilla, Jovito Adiel Skupien, Carlos José Soares, Tatiana Pereira-Cenci. Post removal techniques: a systematic review and meta-analysis. G Ital Endodon 2020. doi:10.32067/GIE.2020.34.01.09.

Reason: SR including different types of subjects

91. Bidra AS, Kejriwal S, Bhuse K. Should Healing Abutments and Cover Screws for Dental Implants be Reused? A Systematic Review. Journal of Prosthodontics. 2020;29:42–8. doi:10.1111/jopr.13106.

Reason: SR including different types of subjects

92. Etemadi A, Hamidain M, Parker S, Chiniforush N. Blue Light Photodynamic Therapy With Curcumin and Riboflavin in the Management of Periodontitis: A Systematic Review. J Lasers Med Sci. 2021;12:e15. doi:10.34172/jlms.2021.15.

Reason: SR including different types of subjects

93. Fonseca J-M, Troconis C-C, Palmier N-R, Gomes-Silva W, Paglioni M-D, Araújo A-L, et al. The impact of head and neck radiotherapy on the dentine-enamel junction: a systematic review. Med Oral Patol Oral Cir Bucal. 2020;25:e96-e105. doi:10.4317/medoral.23212.

Reason: SR including different types of subjects

94. Kiuru O, Sinervo J, Vähänikkilä H, Anttonen V, Tjäderhane L. MMP Inhibitors and Dentin Bonding: Systematic Review and Meta-Analysis. Int J Dent. 2021;2021:9949699. doi:10.1155/2021/9949699.

Reason: SR including different types of subjects

95. Makandar S, Noorani T. Triple antibiotic paste—Challenging intracanal medicament: A systematic review. J Int Oral Health. 2020;12:189. doi:10.4103/JIOH.JIOH_213_19.

Reason: SR including different types of subjects

96. Massé L, Etienne O, Noirrit-Esclassan E, Bailleul-Forestier I, Garot E. Dentine disorders and adhesive treatments: A systematic review. J Dent. 2021;109:103654. doi:10.1016/j.jdent.2021.103654.

Reason: SR including different types of subjects

97. Ranjan, S., Ranjan, M. Antibacterial effectiveness of rotary and reciprocating systems on microbial load reduction in retreatment cases-a systematic review. International Journal of Dentistry and Oral Science. 2021:pp. 3710-3717.

Reason: Full-text not available

98. Rocha FR, Regis WFM, Duarte S, Muniz FWMG, Rodrigues LKA. Effect of bioactive compounds on the regulation of quorum sensing network-associated genes and virulence in Streptococcus mutans-A systematic review. Arch Oral Biol. 2020;119:104893. doi:10.1016/j.archoralbio.2020.104893.

Reason: SR including different types of subjects

99. Santos RBD, Collares K, Brandeburski SBN, Pecho OE, Della Bona A. Experimental methodologies to evaluate the masking ability of dental materials: A systematic review. Journal of esthetic and restorative dentistry : official publication of the American Academy of Esthetic Dentistry … [et al.]. 2021;33:1118–31. doi:10.1111/jerd.12791.

Reason: SR not related to interventions

100. Shah N, Badwaik P, Sheth VH, Bhatnagar V, Bhanushali N, Patil P. Effect of different finish line preparations on the marginal and internal adaptation of cobalt-chromium metal alloy copings fabricated by using CAD-CAM technology: A systematic review and meta-analysis. J Prosthet Dent 2021. doi:10.1016/j.prosdent.2021.06.030.

Reason: Full-text not available

101. Sivakumar N. Antibacterial Effect Of Nanocoating Of Orthodontic Archwires - A Systematic Review. IJDOS. 2014:2244–50. doi:10.19070/2377-8075-21000444.

Reason: SR including different types of subjects

102. Wang T, Matinlinna JP, Burrow MF, Ahmed KE. The biocompatibility of glass-fibre reinforced composites (GFRCs) - a systematic review. J Prosthodont Res. 2021;65:273–83. doi:10.2186/jpr.JPR_D_20_00031.

Reason: Unclear type of study design included

**Additional file 7**: Assessment of AMSTAR-2 items in 185 systematic reviews of in-vitro studies published in dentistry

| Systematic review | AMSTAR-2 items | | | | | | | | | | | | | | | | Total n (%) |
|---|---|---|---|---|---|---|---|---|---|---|---|---|---|---|---|---|---|
| | 1 | 2 | 3 | 4 | 5 | 6 | 7 | 8 | 9 | 10 | 11 | 12 | 13 | 14 | 15 | 16 | |
| Abduo et al. 2011(1) | n | n | n | py | n | n | n | py | n | n | NMA | NMA | n | n | NMA | n | y: 0(0)<br>py: 2(15,4)<br>n: 11(84,6) |
| Adnan et al. 2018(2) | y | y | n | py | y | y | n | y | y | n | NMA | NMA | y | y | NMA | y | y: 9(69,2)<br>py: 1(7,7)<br>n: 3(23,1) |
| Ahn et al. 2016(3) | n | n | n | py | n | n | n | py | n | n | NMA | NMA | n | n | NMA | y | y: 1(7,7)<br>py: 2(15,4)<br>n: 10(76,9) |
| de Almeida et al. 2018(4) | y | y | n | py | y | n | n | y | py | n | n | y | y | n | n | y | y: 7(43,75)<br>py: 2(12,5)<br>n: 7(43,75) |
| Almoudi et al. 2018(5) | n | n | n | py | y | y | n | y | n | n | NMA | NMA | n | n | NMA | y | y: 4(30,8)<br>py: 1(7,7)<br>n: 8(61,5) |
| AlShwaimi et al. 2016(6) | y | py | n | py | y | n | y | y | n | n | NMA | NMA | y | y | NMA | y | y: 7(53,8)<br>py: 2(15,4)<br>n: 4(30,8) |
| Altmann et al. 2016(7) | n | py | n | py | y | y | y | py | n | n | y | y | n | y | y | n | y: 7(43,75)<br>py: 3(18,75)<br>n: 6(37,5) |
| Altmann et al. 2015(8) | y | y | n | py | y | n | y | y | n | n | y | y | n | y | n | y | y: 9(56,25)<br>py: 1(6,25)<br>n: 6(37,5) |
| de Andrade Lima Chaves et al. 2012(9) | n | py | n | py | n | n | y | py | n | n | y | n | n | y | n | n | y: 3(18,75)<br>py: 3(18,75)<br>n: 10(62,5) |
| Archambault et al. 2010(10) | n | n | n | py | n | n | y | py | n | n | NMA | NMA | n | n | NMA | n | y: 1(7,7)<br>py: 2(15,4)<br>n: 10(76,9) |
| Aurélio et al. 2016(11) | y | y | n | py | y | n | n | y | py | n | y | y | y | y | n | n | y: 8(50)<br>py: 2(12,5)<br>n: 6(37,5) |
| Bächle M et al. 2004(12) | y | n | n | py | n | n | n | y | n | n | NMA | NMA | n | y | NMA | n | y: 3(23,1)<br>py: 1(7,7)<br>n: 9(69,2) |
| Baumgartner et al. 2017(13) | y | y | n | y | y | y | n | y | py | n | y | y | y | y | n | n | y: 10(62,5)<br>py: 1(6,25)<br>n: 5(31,25) |
| Behring et al. 2008(14) | y | n | n | n | n | n | n | py | n | n | NMA | NMA | n | n | NMA | n | y: 1(7,7)<br>py: 1(7,7)<br>n: 11(84,6) |
| Bernades Kde et al. 2014(15) | y | n | n | py | n | n | n | y | n | n | NMA | NMA | n | n | NMA | y | y: 3(23,1)<br>py: 1(7,7)<br>n: 9(69,2) |
| Bohrer et al. 2018(16) | y | py | n | py | y | y | n | y | py | n | y | y | y | y | n | n | y: 8(50)<br>py: 3(18,75)<br>n: 5(31,25) |
| Caldas et al. 2019(17) | y | py | n | py | y | y | n | y | n | n | NMA | NMA | n | y | NMA | y | y: 6(46,2)<br>py: 2(15,4)<br>n: 5(38,5) |
| de Carvalho et al. | y | y | n | py | y | n | y | y | y | n | y | y | y | y | n | n | y: 10(62,5) |

| | | | | | | | | | | | | | | | | | |
|---|---|---|---|---|---|---|---|---|---|---|---|---|---|---|---|---|---|
| 2018(18) | | | | | | | | | | | | | | | | | py: 1(6,25)<br>n: 5(31,25) |
| Collares et al. 2016(19) | n | py | n | py | n | y | n | py | n | n | y | n | n | y | n | y | y: 4(25)<br>py: 3(18,75)<br>n: 9(56,25) |
| Collares et al. 2013(20) | n | py | n | py | n | y | n | py | n | n | y | n | n | y | n | n | y: 3(18,75)<br>py: 3(18,75)<br>n: 10(62,5) |
| Coray et al. 2016(21) | y | py | n | py | y | n | y | y | n | n | n | n | n | n | n | n | y: 4(25)<br>py: 2(12,5)<br>n: 10(62,5) |
| Corrêa et al. 2019(22) | y | py | n | py | y | y | n | y | py | n | y | y | y | y | n | n | y: 8(50)<br>py: 3(18,75)<br>n: 5(31,25) |
| Cuevas-Suárez et al. 2019(23) | n | y | n | py | y | n | y | py | py | n | y | y | n | y | n | n | y: 6(37,5)<br>py: 3(18,75)<br>n: 7(43,75) |
| Cury et al. 2019(24) | y | py | n | y | y | y | n | y | y | n | NMA | NMA | y | y | NMA | y | y: 9(69,2)<br>py: 1(7,7)<br>n: 3(23,1) |
| Davoudi et al. 2019(25) | y | py | n | py | y | y | n | py | y | n | NMA | NMA | n | y | NMA | n | y: 5(31,25)<br>py: 3(18,75)<br>n: 5(31,25) |
| Dumbryte et al. 2018(26) | y | y | n | py | y | y | y | y | y | n | y | y | y | y | n | y | y: 12(75)<br>py: 1(6,25)<br>n: 3(18,75) |
| Ehsani et al. 2009(27) | n | py | n | py | n | n | y | py | n | n | NMA | NMA | n | n | NMA | n | y: 1(7,7)<br>py: 3(23,1)<br>n: 9(69,2) |
| Ekambaram et al. 2015(28) | y | py | n | py | n | n | y | y | n | n | NMA | NMA | n | n | NMA | n | y: 3(23,1)<br>py: 2(15,4)<br>n: 8(61,5) |
| Ferreira et al. 2017(29) | n | n | n | py | n | n | n | py | n | n | NMA | NMA | n | n | NMA | y | y: 1(7,7)<br>py: 2(15,4)<br>n: 10(76,9) |
| Finnema et al. 2010(30) | n | py | n | py | n | y | n | py | n | n | y | n | n | y | n | y | y: 4(25)<br>py: 3(18,75)<br>n: 9(56,25) |
| García-Sanz et al. 2018(31) | n | y | n | y | y | n | n | py | n | n | y | y | y | y | y | y | y: 9(56,25)<br>py: 1(6,25)<br>n: 6(37,5) |
| Gorman et al. 2016(32) | y | n | n | y | n | y | n | py | y | y | NMA | NMA | n | n | NMA | y | y: 6(46,2)<br>py: 1(7,7)<br>n: 6(46,2) |
| Goujat et al. 2019(33) | y | py | n | py | y | n | n | y | py | n | NMA | NMA | Y | Y | NMA | y | y: 6(46,2)<br>py: 3(23,1)<br>n: 4(30,8) |
| Grewal Bach et al. 2014(34) | y | n | n | py | y | n | y | n | n | n | NMA | NMA | n | n | NMA | n | y: 3(23,1)<br>py: 1(7,7)<br>n: 9(69,2) |
| Holliday et al. 2019(35) | y | py | n | y | y | n | y | py | y | y | NMA | NMA | y | y | NMA | y | y: 9(69,2)<br>py: 2(15,4)<br>n: 2(15,4) |
| Iliadi et al. 2019(36) | y | y | n | y | y | n | n | y | py | n | y | y | y | y | n | y | y: 10(62,5)<br>py: 1(6,25)<br>n: 5(31,25) |
| Isolan et al. 2015(37) | y | y | n | py | y | n | y | y | py | n | y | y | y | y | n | n | y: 9(56,25)<br>py: 2(12,5)<br>n: 5(31,25) |
| Kaczor et al. 2018(38) | y | y | n | py | y | n | n | y | py | n | y | y | y | n | n | y | y: 8(50)<br>py: 2(12,5) |

| | | | | | | | | | | | | | | | | | |
|---|---|---|---|---|---|---|---|---|---|---|---|---|---|---|---|---|---|
| | | | | | | | | | | | | | | | | | n: 6(37,5) |
| Kaizer et al. 2014(39) | y | py | n | py | y | y | y | y | n | n | NMA | NMA | n | y | NMA | n | y: 6(46,2)<br>py: 2(15,4)<br>n: 5(38,5) |
| Kaufmann et al. 2018(40) | y | py | n | py | y | n | y | y | n | n | NMA | NMA | n | n | NMA | y | y: 5(38,5)<br>py: 2(15,4)<br>n: 6(46,2) |
| Kim et al. 2015(41) | y | n | n | py | y | y | y | py | n | n | NMA | NMA | n | n | NMA | n | y: 4(30,8)<br>py: 2(15,4)<br>n: 7(53,8) |
| Kuik et al. 2019(42) | y | py | n | py | n | n | py | y | n | n | NMA | NMA | n | y | NMA | y | y: 4(30,8)<br>py: 3(23,1)<br>n: 6(46,2) |
| Laaksonen et al. 2010(43) | n | n | n | py | n | n | n | py | n | n | NMA | NMA | n | n | NMA | y | y: 1(7,7)<br>py: 2(15,4)<br>n: 10(76,9) |
| Lee et al. 2008(44) | n | n | n | py | n | n | n | py | n | n | NMA | NMA | n | n | NMA | n | y: 0(0)<br>py: 2(15,4)<br>n: 11(84,6) |
| Lenzi et al. 2016(45) | n | y | n | py | y | n | n | py | py | n | y | y | y | y | n | y | y: 7(43,75)<br>py: 3(18,75)<br>n: 6(37,5) |
| Lo Russo et al. 2019(46) | y | py | n | py | y | y | n | y | n | n | y | n | n | y | n | n | y: 6(37,5)<br>py: 2(12,5)<br>n: 8(50) |
| Lombardo et al. 2019(47) | n | py | n | py | y | n | n | py | n | n | n | n | n | n | n | n | y: 1(6,25)<br>py: 3(18,75)<br>n: 12(75) |
| Louropoulou et al. 2015(48) | y | y | n | py | y | y | y | y | py | n | NMA | NMA | y | y | NMA | y | y: 9(69,2)<br>py: 2(15,4)<br>n: 2(15,4) |
| Marchionatti et al. 2018(49) | y | py | n | py | y | y | n | y | y | n | y | y | y | y | n | n | y: 9(56,25)<br>py: 2(12,5)<br>n: 5(31,25) |
| Martins et al. 2019(50) | y | y | n | py | y | n | n | y | py | n | y | y | n | y | n | y | y: 8(50)<br>py: 2(12,5)<br>n: 6(37,5) |
| Martins et al. 2019(51) | y | y | n | py | y | n | n | y | py | n | y | y | n | y | n | y | y: 8(50)<br>py: 2(12,5)<br>n: 6(37,5) |
| Masarwa et al. 2016(52) | y | y | n | n | n | n | n | py | py | n | y | y | y | y | y | n | y: 7(43,75)<br>py: 2(12,5)<br>n: 7(43,75) |
| de Mello et al. 2018(53) | y | py | n | py | y | n | y | y | n | n | n | n | y | n | n | y | y: 6(37,5)<br>py: 2(12,5)<br>n: 8(50) |
| Mello et al. 2019(54) | y | py | n | py | y | y | y | y | y | y | y | n | n | y | n | y | y: 10(62,5)<br>py: 2(12,5)<br>n: 4(25) |
| Menezes-Silva et al. 2019(55) | y | py | n | py | y | n | n | py | py | n | NMA | NMA | n | n | NMA | n | y: 2(15,4)<br>py: 4(30,8)<br>n: 7(53,8) |
| Moharrami et al. 2019(56) | y | n | n | py | y | n | y | y | n | n | NMA | NMA | n | n | NMA | y | y: 5(38,5)<br>py: 1(7,7)<br>n: 7 (53,8) |
| Moraes et al. 2015(57) | n | y | n | py | y | n | y | py | py | n | y | y | y | y | n | y | y: 8(50)<br>py: 3(18,75)<br>n: 5(31,25) |
| Moreira et al. 2015(58) | n | n | n | py | n | y | n | y | n | n | NMA | NMA | n | n | NMA | n | y: 2(15,4)<br>py: 1(7,7) |

| | | | | | | | | | | | | | | | | | |
|---|---|---|---|---|---|---|---|---|---|---|---|---|---|---|---|---|---|
| | | | | | | | | | | | | | | | | | n: 10(76,9) |
| Możyńska et al. 2017(59) | n | py | n | py | y | n | n | py | py | n | NMA | NMA | y | y | NMA | y | y: 4(30,8)<br>py: 4(30,8)<br>n: 5(38,5) |
| Nagendrababu et al. 2018(60) | y | py | n | py | y | y | y | y | n | n | NMA | NMA | n | y | NMA | y | y: 7(53,8)<br>py: 2 (15,4)<br>n: 4(30,8) |
| Nassar et al. 2011(61) | y | n | n | py | y | n | n | y | n | n | NMA | NMA | n | n | NMA | n | y: 3(23,1)<br>py: 1(7,7)<br>n: 9(69,2) |
| Nawafleh et al. 2016(62) | n | n | n | py | y | n | y | py | n | n | NMA | NMA | n | y | NMA | y | y: 4(30,8)<br>py: 2(15,4)<br>n: 7(53,8) |
| Neelakantan et al. 2018(63) | y | py | n | py | y | y | y | y | py | n | NMA | NMA | y | y | NMA | y | y: 8(61,5)<br>py: 3(23,1)<br>n: 2(15,4) |
| Ntrouka et al. 2011(64) | n | y | n | py | y | y | y | py | py | n | NMA | NMA | y | y | NMA | y | y: 7(53,8)<br>py: 3(23,1)<br>n: 3(23,1) |
| Oliveira et al. 2018(65) | y | py | n | py | y | n | y | y | py | n | NMA | NMA | y | y | NMA | y | y: 7(53,8)<br>py: 3(23,1)<br>n: 3(23,1) |
| Oliveira et al. 2020(66) | y | y | n | py | y | y | n | y | py | n | y | y | y | y | n | n | y: 9(56,25)<br>py: 2(12,5)<br>n: 5(31,25) |
| Osmanovic et al. 2018(67) | y | py | n | py | n | n | n | y | py | n | NMA | NMA | n | n | NMA | y | y: 3(23,1)<br>py: 3(23,1)<br>n: 7(53,8) |
| Özcan et al. 2015(68) | n | py | n | py | y | y | n | n | n | n | n | n | n | n | n | n | y: 2(12,5)<br>py: 2(12,5)<br>n: 12(75) |
| Özcan et al. 2018(69) | y | py | n | py | y | y | n | y | n | n | NMA | NMA | n | n | NMA | y | y: 5(38,5)<br>py: 2(15,4)<br>n: 6(46,2) |
| Özcan et al. 2018(70) | y | n | n | n | n | n | n | y | n | n | NMA | NMA | n | n | NMA | y | y: 3(23,1)<br>py: 0(0)<br>n: 10(76,9) |
| Papia et al. 2014(71) | n | n | n | py | n | n | n | py | n | n | NMA | NMA | n | n | NMA | n | y: 0(0)<br>py: 2(15,4)<br>n: 11(84,6) |
| Pardal-Peláez et al. 2017(72) | y | n | n | py | n | n | n | y | n | n | NMA | NMA | n | n | NMA | y | y: 3(23,1)<br>py: 1(7,7)<br>n: 9(69,2) |
| Parikh et al. 2019(73) | y | py | n | py | n | y | n | y | y | n | NMA | NMA | y | y | NMA | y | y: 7(53,8)<br>py: 2(15,4)<br>n: 4(30,8) |
| Passos et al. 2015(74) | n | py | n | py | y | n | n | py | n | n | NMA | NMA | n | y | NMA | y | y: 3(23,1)<br>py: 3(23,1)<br>n: 7(53,8) |
| Passos et al. 2014(75) | n | n | n | py | y | n | n | py | n | n | NMA | NMA | n | n | NMA | y | y: 2(15,4)<br>py: 2(15,4)<br>n: 9(69,2) |
| Perroni et al. 2018(76) | y | py | n | py | y | n | y | y | y | y | NMA | NMA | n | n | NMA | y | y: 7(53,8)<br>py: 2(15,4)<br>n: 4(30,8) |
| Pinho MM et al. 2017(77) | y | n | n | py | y | y | n | y | n | n | NMA | NMA | n | n | NMA | y | y: 5(38,5)<br>py: 1(7,7)<br>n: 7(53,8) |
| Pires et al. 2018(78) | y | y | n | py | y | y | n | y | y | n | y | y | y | y | n | n | y: 10(62,5)<br>py: 1(6,25) |

| | | | | | | | | | | | | | | | | | |
|---|---|---|---|---|---|---|---|---|---|---|---|---|---|---|---|---|---|
| | | | | | | | | | | | | | | | | | n: 5(31,25) |
| Reis et al. 2017(79) | y | n | n | py | y | y | n | y | n | n | NMA | NMA | n | n | NMA | y | y: 5 (38,5)<br>py: 1(7,7)<br>n: 7(53,8) |
| Resende et al. 2020(80) | y | py | n | py | n | n | n | y | y | n | NMA | NMA | y | y | NMA | y | y: 6(46,2)<br>py: 2(15,4)<br>n: 5(38,5) |
| Rodrigues et al. 2019(81) | y | y | n | py | y | y | n | y | y | y | y | y | y | y | n | n | y: 11(68,75)<br>py: 1(6,25)<br>n: 4(25) |
| Rosa et al. 2015(82) | n | y | n | py | y | n | y | py | py | n | n | y | n | n | n | n | y: 4(25)<br>py: 3(18,75)<br>n: 9(56,25) |
| Samiei et al. 2019(83) | n | py | n | py | y | y | n | py | py | n | NMA | NMA | n | n | NMA | y | y: 3(23,1)<br>py: 4(30,8)<br>n: 6(46,2) |
| Sarkis-Onofre et al. 2014(84) | y | y | n | py | y | n | n | y | py | n | y | y | y | y | n | y | y: 9(56,25)<br>py: 2(12,5)<br>n: 5(31,25) |
| Shahmiri et al. 2018(85) | y | n | n | py | n | n | y | y | n | n | NMA | NMA | n | n | NMA | n | y: 3(23,1)<br>py: 1(7,7)<br>n: 9(69,2) |
| Shahravan et al. 2007(86) | y | py | n | n | n | n | py | y | n | n | y | n | n | y | y | n | y: 5(31,25)<br>py: 2(12,5)<br>n: 9(56,25) |
| Silva et al. 2019(87) | y | y | n | y | y | y | y | y | n | n | y | y | y | y | n | n | y: 11(68,75)<br>py: 0(0)<br>n: 5(31,25) |
| Silva et al. 2018(88) | y | py | n | y | y | y | y | y | n | n | NMA | NMA | y | n | NMA | n | y: 7(53,8)<br>py: 1(7,7)<br>n: 5(38,5) |
| Skupien et al. 2015(89) | y | n | n | py | y | y | n | py | n | n | NMA | NMA | n | y | NMA | y | y: 5(38,5)<br>py: 2(15,4)<br>n: 6(46,2) |
| Soares et al. 2016(90) | y | y | n | py | y | n | y | y | py | n | y | y | y | y | n | n | y: 9(56,25)<br>py: 2(12,5)<br>n: 5(31,25) |
| Solanki et al. 2018(91) | y | py | n | py | y | y | n | n | n | y | NMA | NMA | n | n | NMA | y | y: 5(38,5)<br>py: 2(15,4)<br>n: 6(46,2) |
| de Sousa et al. 2018(92) | y | py | n | py | y | n | n | y | n | n | NMA | NMA | n | n | NMA | y | y: 4(30,8)<br>py: 2(15,4)<br>n: 7(53,8) |
| Strauss et al. 2020(93) | y | n | n | py | y | y | n | y | n | n | NMA | NMA | n | n | NMA | y | y: 5(38,5)<br>py: 1(7,7)<br>n: 7(53,8) |
| Tageldin et al. 2016(94) | n | py | n | py | n | n | n | n | n | n | NMA | NMA | n | n | NMA | n | y: 0(0)<br>py: 2(15,4)<br>n: 11(84,6) |
| Taha et al. 2017(95) | y | y | n | y | n | y | n | y | py | n | NMA | NMA | n | y | NMA | n | y: 6(46,2)<br>py: 1(7,7)<br>n: 6(46,2) |
| Tavares et al. 2019(96) | y | py | n | y | y | n | n | y | y | n | NMA | NMA | n | n | NMA | y | y: 6(46,2)<br>py: 1(7,7)<br>n: 6(46,2) |
| Theodosopoulou et al. 2005(97) | y | py | n | n | y | n | y | y | py | n | NMA | NMA | n | y | NMA | n | y: 5 (38,5)<br>py: 2(15,4)<br>n: 6(46,2) |
| Ting et al. 2016(98) | n | n | n | py | n | y | n | py | n | n | NMA | NMA | n | n | NMA | y | y: 2(15,4)<br>py: 2(15,4) |

| | | | | | | | | | | | | | | | | | |
|---|---|---|---|---|---|---|---|---|---|---|---|---|---|---|---|---|---|
| | | | | | | | | | | | | | | | | | n: 9(69,2) |
| Tsesis et al. 2015(99) | y | py | n | py | y | y | n | n | y | n | n | y | n | n | n | y | y: 6(37,5)<br>py: 2(12,5)<br>n: 8(50) |
| Tzanakakis et al. 2016(100) | n | n | n | py | n | n | n | py | n | n | NMA | NMA | n | n | NMA | n | y: 0(0)<br>py: 2(15,4)<br>n: 11(84,6) |
| Uzunoglu-Özyürek et al. 2018(101) | y | py | n | py | y | n | y | y | py | n | NMA | NMA | y | y | NMA | y | y: 7(53,8)<br>py: 3(23,1)<br>n: 3(23,1) |
| Valente et al. 2016(102) | y | y | n | py | y | y | py | y | py | n | y | y | y | y | n | n | y: 9(56,25)<br>py: 3(18,75)<br>n: 4(25) |
| Virdee et al. 2018(103) | y | y | n | py | y | n | y | y | y | n | y | y | y | y | y | y | y: 12(75)<br>py: 1(6,25)<br>n: 3(18,75) |
| Western et al. 2016(104) | n | n | n | py | n | n | y | y | n | n | NMA | NMA | n | n | NMA | y | y: 3(23,1)<br>py: 1(7,7)<br>n: 9(69,2) |
| Yassen et al. 2013(105) | n | n | n | py | n | n | y | py | n | n | NMA | NMA | n | n | NMA | n | y: 1(7,7)<br>py: 2(15,4)<br>n: 10(76,9) |
| Yaylali et al. 2015(106) | y | py | n | py | y | y | y | y | py | n | NMA | NMA | y | y | NMA | y | y: 8(61,5)<br>py: 3(23,1)<br>n: 2(15,4) |
| Yu et al. 2019(107) | n | y | n | py | y | n | n | py | n | n | y | y | y | y | n | n | y: 6(37,5)<br>py: 2(12,5)<br>n: 8(50) |
| Yu et al. 2020(108) | y | y | n | py | y | y | n | y | py | n | y | y | y | y | n | n | y: 9(56,25)<br>py: 2(12,5)<br>n: 5(31,25) |
| Ahmed et al. 2020(109) | y | py | n | py | y | n | y | y | n | n | NMA | NMA | y | y | NMA | y | y: 7(53,8)<br>py: 2(15,4)<br>n: 4(30,8) |
| Alammar et al. 2021(110) | y | py | n | py | y | y | y | y | n | n | NMA | NMA | n | y | NMA | y | y: 7(53,8)<br>py: 2(15,4)<br>n: 4(30,8) |
| Alhajj et al. 2021(111) | y | y | n | py | n | n | n | y | y | n | y | n | y | y | y | y | y: 9(56,25)<br>py: 1(6,25)<br>n: 6(37,5) |
| Aljomard et al. 2021(112) | n | py | n | py | y | n | n | py | n | n | y | n | n | y | y | y | y: 5(31,25)<br>py: 3(18,75)<br>n: 8(50) |
| Almeida Bastos et al. 2020(113) | y | py | n | n | y | n | y | y | n | n | y | n | n | y | n | y | y: 7(43,75)<br>py: 1(6,25)<br>n: 8(50) |
| Al-Thobity et al. 2021(114) | y | y | n | y | n | n | n | y | y | y | y | n | n | y | n | n | y: 8(50)<br>py: 0(0)<br>n: 8(50) |
| Anita et al. 2021(115) | y | y | n | py | y | y | y | y | py | n | y | y | y | y | y | y | y: 12(75)<br>py: 2(12,5)<br>n: 2(12,5) |
| Astudillo-Rubio et al. 2018(116) | y | y | n | y | y | y | n | y | py | n | y | n | y | y | y | y | y: 11(68,75)<br>py: 1(6,25)<br>n: 4(25) |
| Baldion et al. 2020(117) | y | py | n | y | y | y | y | y | py | n | NMA | NMA | y | y | NMA | y | y: 9(69,2)<br>py: 2(15,4)<br>n: 2(15,4) |
| Bangera et al. 2021(118) | y | y | n | py | y | n | n | y | y | y | y | y | y | y | n | y | y: 11(68,75)<br>py: 1(6,25) |

| | | | | | | | | | | | | | | | | | |
|---|---|---|---|---|---|---|---|---|---|---|---|---|---|---|---|---|---|
| | | | | | | | | | | | | | | | | | n: 4(25) |
| Bangera et al. 2020(119) | y | y | n | py | y | y | n | y | y | y | y | y | n | y | n | y | y: 11(68,75)<br>py: 1(6,25)<br>n: 4(25) |
| Bitencourt et al. 2021(120) | y | n | n | py | n | n | n | n | n | n | n | n | n | n | n | y | y: 2(12,5)<br>py: 1(6,25)<br>n: 13(81,25) |
| Bohrer et al. 2021(121) | y | y | n | py | y | y | n | y | py | n | y | n | y | y | n | y | y: 9(56,25)<br>py: 2(12,5)<br>n: 5(31,25) |
| Bonzanini et al. 2021(122) | y | y | n | py | y | y | y | y | py | n | y | y | y | y | n | y | y: 11(68,75)<br>py: 2(12,5)<br>n: 3(18,75) |
| Carneiro Pereira et al. 2021(123) | n | py | n | py | n | n | n | py | py | n | NMA | NMA | n | y | NMA | n | y: 1(7,7)<br>py: 4(30,8)<br>n: 8(61,5) |
| Cavalcante-Leão et al. 2021(124) | y | y | n | y | y | y | y | y | y | y | NMA | NMA | n | n | NMA | y | y: 10(76,9)<br>py: 0(0)<br>n: 3(23,1) |
| Corvino et al. 2020(125) | y | py | n | py | y | y | y | y | py | n | NMA | NMA | y | y | NMA | y | y: 8(61,5)<br>py: 3(23,1)<br>n: 2(15,4) |
| Costa et al. 2021(126) | y | y | n | n | y | y | n | y | y | n | y | y | y | n | n | y | y: 10(62,5)<br>py: 0(0)<br>n: 6(37,5) |
| da Costa et al. 2021(127) | y | py | n | py | n | y | n | y | y | n | NMA | NMA | n | y | NMA | y | y: 6(46,2)<br>py: 2(15,4)<br>n: 5(38,5) |
| David et al. 2021(128) | y | y | n | py | y | n | n | y | py | n | y | y | n | y | n | n | y: 7(43,75)<br>py: 2(12,5)<br>n: 7(43,75) |
| Eswaramurthy et al. 2021(129) | y | py | n | y | y | y | y | y | y | n | NMA | NMA | n | n | NMA | y | y: 8(61,5)<br>py: 1(7,7)<br>n: 4(30,8) |
| Fathy et al. 2021(130) | n | n | n | py | y | n | n | py | n | n | NMA | NMA | n | n | NMA | n | y: 1(7,7)<br>py: 2(15,4)<br>n: 10(76,9) |
| Fernández-Barrera et al. 2021(131) | y | y | n | py | y | n | y | y | py | n | y | n | n | n | y | n | y: 7(43,75)<br>py: 2(12,5)<br>n: 7(43,75) |
| Ferreira et al. 2021(132) | y | py | n | py | y | y | y | py | py | n | NMA | NMA | y | y | NMA | y | y: 7(53,8)<br>py: 4(30,8)<br>n: 2(15,4) |
| Fröhlich et al. 2021(133) | y | y | n | py | y | n | y | y | py | n | y | y | y | y | n | y | y: 10(62,5)<br>py: 2(12,5)<br>n: 4(25) |
| Fröhlich et al. 2019(134) | y | y | n | py | y | n | y | y | py | n | y | n | y | y | n | y | y: 9(56,25)<br>py: 2(12,5)<br>n: 5(31,25) |
| Gerula-Szymanska et al. 2020(135) | y | y | n | py | n | n | n | y | py | n | y | n | n | y | n | y | y: 6(37,5)<br>py: 2(12,5)<br>n: 8(50) |
| Golshah et al. 2020(136) | y | py | n | py | y | n | n | y | y | n | y | y | n | n | y | y | y: 8(50)<br>py: 2(12,5)<br>n: 6(37,5) |
| Iaculli et al. 2021(137) | y | y | n | py | y | y | y | y | py | n | y | y | y | y | y | y | y: 12(75)<br>py: 2(12,5)<br>n: 2(12,5) |
| Jacob et al. 2021(138) | n | y | n | py | n | n | n | py | py | n | y | y | y | y | n | y | y: 6(37,5)<br>py: 3(18,75) |

| | | | | | | | | | | | | | | | | |
|---|---|---|---|---|---|---|---|---|---|---|---|---|---|---|---|---|
| | | | | | | | | | | | | | | | | n: 7(43,75) |
| Janjic et al. 2018(139) | n | n | n | py | y | n | y | py | n | n | NMA | NMA | n | y | NMA | y | y: 4(30,8)<br>py: 2(15,4)<br>n: 7(53,8) |
| Jurema et al. 2021(140) | y | y | n | y | n | y | n | y | py | n | y | y | y | y | n | n | y: 9(56,25)<br>py: 1(6,25)<br>n: 6(37,5) |
| Kreve et al. 2021(141) | n | py | n | py | y | n | n | n | n | n | NMA | NMA | n | y | NMA | y | y: 3(23,1)<br>py: 2(15,4)<br>n: 8(61,5) |
| Kwon et al. 2020(142) | y | py | n | n | y | n | y | y | py | y | n | n | n | y | n | y | y: 7(43,75)<br>py: 2(12,5)<br>n: 7(43,75) |
| Li et al. 2021(143) | y | py | n | y | y | y | n | y | py | n | | y | y | y | n | n | y: 9(56,25)<br>py: 2(12,5)<br>n: 5(31,25) |
| Li et al. 2019(144) | n | py | n | py | n | n | n | py | py | n | NMA | NMA | y | n | NMA | n | y: 1(7,7)<br>py: 4(30,8)<br>n: 8(61,5) |
| Lima et al. 2020(145) | y | y | n | py | y | n | y | y | py | n | y | n | n | y | n | y | y: 8(50)<br>py: 2(12,5)<br>n: 6(37,5) |
| Lin et al. 2021(146) | n | n | n | py | y | n | n | py | n | n | NMA | NMA | n | n | NMA | y | y: 2(15,4)<br>py: 2(15,4)<br>n: 9(69,2) |
| Mai et al. 2020(147) | y | y | n | py | y | y | n | y | y | n | y | y | y | y | y | n | y: 11(68,75)<br>py: 1(6,25)<br>n: 4(25) |
| Mendes et al. 2020(148) | y | y | n | py | y | y | n | y | py | n | y | n | y | y | n | n | y: 8(50)<br>py: 2(12,5)<br>n: 6(37,5) |
| Mishra et al. 2020(149) | n | py | n | py | y | n | n | py | py | n | NMA | NMA | y | y | NMA | y | y: 4(30,8)<br>py: 4(30,8)<br>n: 5(38,5) |
| Mohamed et al. 2021(150) | y | py | n | py | n | y | n | y | y | n | NMA | NMA | n | y | NMA | y | y: 6(46,2)<br>py: 2(15,4)<br>n: 5(38,5) |
| Moura et al. 2021(151) | y | y | n | py | y | n | n | y | py | n | y | n | y | y | n | n | y: 7(43,75)<br>py: 2(12,5)<br>n: 7(43,75) |
| Mustafa et al. 2020(152) | n | n | n | py | n | y | n | py | n | n | NMA | NMA | n | y | NMA | y | y: 3(23,1)<br>py: 2(15,4)<br>n: 8(61,5) |
| Nimbeni et al. 2021(153) | n | n | n | py | n | n | n | py | n | n | NMA | NMA | n | n | NMA | n | y: 0(0)<br>py: 2(15,4)<br>n: 11(84,6) |
| Nogueira et al. 2021(154) | y | y | n | py | y | n | n | y | py | n | y | y | y | y | n | n | y: 8(50)<br>py: 2(12,5)<br>n: 6(37,5) |
| de Oliveira et al. 2021(155) | y | y | n | py | y | y | y | py | y | y | y | n | n | y | n | y | y: 10(62,5)<br>py: 2(12,5)<br>n: 4(25) |
| de Oliveira Limírio et al. 2021(156) | y | y | n | py | y | y | n | y | y | n | y | y | y | y | n | n | y: 10(62,5)<br>py: 1(6,25)<br>n: 5(31,25) |
| Özcan et.al. 2014(157) | n | py | n | n | y | y | n | n | n | n | n | n | n | n | n | n | y: 2(12,5)<br>py: 1(6,25)<br>n: 13(81,25) |
| Parize et al. 2021(158) | y | y | n | py | y | y | y | y | n | n | NMA | NMA | y | y | NMA | y | y: 9(69,2)<br>py: 1(7,7) |

| | | | | | | | | | | | | | | | | | |
|---|---|---|---|---|---|---|---|---|---|---|---|---|---|---|---|---|---|
| | | | | | | | | | | | | | | | | | n: 3(23,1) |
| Parolia et al. 2020(159) | y | py | n | py | y | y | y | y | py | n | NMA | NMA | y | y | NMA | y | y: 8(61,5)<br>py: 3(23,1)<br>n: 2(15,4) |
| Paul et al. 2021(160) | y | py | n | py | n | y | y | y | py | n | NMA | NMA | n | y | NMA | y | y: 6(46,2)<br>py: 3(23,1)<br>n: 4(30,8) |
| Pereira et. al. 2016(161) | y | y | n | py | y | y | n | y | py | n | y | n | y | y | n | n | y: 8(50)<br>py: 2(12,5)<br>n: 6(37,5) |
| Pires Lopes et al. 2020(162) | n | py | n | py | y | n | y | py | py | n | NMA | NMA | n | n | NMA | n | y: 2(15,4)<br>py: 4(30,8)<br>n: 7(53,8) |
| Portela et al. 2021(163) | y | py | n | y | y | n | y | y | y | y | NMA | NMA | y | n | NMA | y | y: 9(69,2)<br>py: 1(7,7)<br>n: 3(23,1) |
| Pourhajibagher et al. 2020(164) | y | y | n | py | n | n | n | py | py | n | y | y | n | y | y | y | y: 7(43,75)<br>py: 3(18,75)<br>n: 6(37,5) |
| Purger et al. 2021(165) | y | py | n | py | y | n | n | y | py | n | NMA | NMA | y | n | NMA | y | y: 5(38,5)<br>py: 3(23,1)<br>n: 5(38,5) |
| dos Reis-Prado et al. 2021(166) | y | y | n | py | y | n | y | y | y | n | y | y | y | y | n | y | y: 11(68,75)<br>py: 1(6,25)<br>n: 4(25) |
| Revilla-León et al. 2021(167) | y | y | n | py | n | y | n | y | y | n | y | y | y | y | y | y | y: 11(68,75)<br>py: 1(6,25)<br>n: 4(25) |
| Rodríguez-Barragué et al. 2021(168) | y | py | n | py | y | n | y | py | py | n | y | n | y | y | n | y | y: 7(43,75)<br>py: 4(25)<br>n: 5(31,25) |
| Saeed et al. 2021(169) | y | py | n | py | y | y | n | y | py | n | NMA | NMA | y | n | NMA | y | y: 6(46,2)<br>py: 3(23,1)<br>n: 4(30,8) |
| Sanz et al. 2020(170) | y | py | n | py | y | y | y | y | y | y | NMA | NMA | n | y | NMA | y | y: 9(69,2)<br>py: 2(15,4)<br>n: 2(15,4) |
| Sanz et al. 2020(171) | y | py | n | py | y | y | y | y | y | y | NMA | NMA | n | y | NMA | y | y: 9(69,2)<br>py: 2(15,4)<br>n: 2(15,4) |
| Sanz et al. 2021(172) | y | n | n | py | y | y | n | y | y | y | NMA | NMA | y | n | NMA | y | y: 8(61,5)<br>py: 1(7,7)<br>n: 4(30,8) |
| Sanz et al. 2021(173) | y | n | n | py | y | y | y | y | y | y | NMA | NMA | y | y | NMA | n | y: 9(69,2)<br>py: 1(7,7)<br>n: 3(23,1) |
| Savian et al. 2021(174) | y | y | n | py | y | n | n | y | py | n | y | n | y | y | n | y | y: 8(50)<br>py: 2(12,5)<br>n: 6(37,5) |
| Silva et al. 2021(175) | n | py | n | y | y | y | y | py | py | n | y | y | y | n | n | y | y: 8(50)<br>py: 3(18,75)<br>n: 5(31,25) |
| Spitz et al. 2020(176) | y | y | n | y | y | n | y | py | y | y | NMA | NMA | y | y | NMA | y | y: 10(76,9)<br>py: 1(7,7)<br>n: 2(15,4) |
| Stasic et al. 2021(177) | y | y | n | py | n | y | n | y | py | n | y | y | y | y | y | y | y: 10(62,5)<br>py: 2(12,5)<br>n: 4(25) |
| Tan et al. 2021(178) | y | py | n | py | y | y | n | y | y | y | NMA | NMA | n | y | NMA | y | y: 8(61,5)<br>py: 2(15,4) |

| | | | | | | | | | | | | | | | | | |
|---|---|---|---|---|---|---|---|---|---|---|---|---|---|---|---|---|---|
| | | | | | | | | | | | | | | | | | n: 3(23,1) |
| Teja et al. 2021(179) | y | py | n | py | n | n | n | y | py | n | NMA | NMA | n | n | NMA | y | y: 3(23,1)<br>py: 3(23,1)<br>n: 7(53,8) |
| Thakur et al. 2021(180) | y | y | n | py | y | n | n | y | py | n | y | y | y | y | y | y | y: 10(62,5)<br>py: 2(12,5)<br>n: 4(25) |
| Troconis et al. 2021(181) | y | y | n | py | n | n | n | y | py | n | NMA | NMA | y | y | NMA | n | y: 5(38,5)<br>py: 2(15,4)<br>n: 6(46,2) |
| Uzunoglu-Özyürek et al. 2021(182) | y | y | n | py | y | n | y | y | py | n | y | n | y | y | n | y | y: 9(56,25)<br>py: 2(12,5)<br>n: 5(31,25) |
| Wang et al. 2020(183) | y | y | n | py | n | n | n | y | py | n | NMA | NMA | n | y | NMA | n | y: 4(30,8)<br>py: 2(15,4)<br>n: 7(53,8) |
| Wehner et al. 2020(184) | y | py | n | py | y | y | n | y | n | n | y | n | n | y | y | y | y: 8(50)<br>py: 2(12,5)<br>n: 6(37,5) |
| Willis et al. 2021(185) | y | n | n | py | n | n | n | y | n | y | NMA | NMA | n | n | NMA | y | y: 4(30,8)<br>py: 1(7,7)<br>n: 8(61,5) |

y:yes; py:partial yes; n=no; u:unclear; NMA: no meta-analysis
